# Promotion of B(C_6_F_5_)_3_ as Ligand for Titanium (or Vanadium) Catalysts in the Copolymerization of Ethylene and 1-Hexene: A Computational Study

**DOI:** 10.3390/polym15112435

**Published:** 2023-05-24

**Authors:** Shuyuan Yu, Chenggen Zhang, Fei Wang, Xinru Liang, Mengyao Yang, Mengyu An

**Affiliations:** College of Chemistry and Material Science, Langfang Normal University, Langfang 065000, China; yushuyuan05@mails.ucas.ac.cn (S.Y.); wangfei20220726@163.com (F.W.); liangxinru2023@163.com (X.L.); yangmengyao2023@163.com (M.Y.); anmengyu1466850030@163.com (M.A.)

**Keywords:** ethylene, 1-hexene, copolymerization, titanium (or vanadium) catalyst, DFT calculations

## Abstract

Density functional theory (DFT) is employed to investigate the promotion of B(C_6_F_5_)_3_ as a ligand for titanium (or vanadium) catalysts in ethylene/1-hexene copolymerization reactions. The results reveal that (I) Ethylene insertion into **Ti_B_** (with B(C_6_F_5_)_3_ as a ligand ) is preferred over **Ti_H_**_,_ both thermodynamically and kinetically. (II) In **Ti_H_** and **Ti_B_** catalysts, the 2,1 insertion reaction (**Ti_H21_** and **Ti_B21_**) is the primary pathway for 1-hexene insertion. Furthermore, the 1-hexene insertion reaction for **Ti_B21_** is favored over **Ti_H21_** and is easier to perform. Consequently, the entire ethylene and 1-hexene insertion reaction proceeds smoothly using the **Ti_B_** catalyst to yield the final product. (III) Analogous to the **Ti** catalyst case, **V_B_** (with B(C_6_F_5_)_3_ as a ligand) is preferred over **V_H_** for the entire ethylene/1-hexene copolymerization reaction. Moreover, **V_B_** exhibits higher reaction activity than **Ti_B_**, thus agreeing with experimental results. Additionally, the electron localization function and global reactivity index analysis indicate that titanium (or vanadium) catalysts with B(C_6_F_5_)_3_ as a ligand exhibit higher reactivity. Investigating the promotion of B(C_6_F_5_)_3_ as a ligand for titanium (or vanadium) catalysts in ethylene/1-hexene copolymerization reactions will aid in designing novel catalysts and lead to more cost-effective polymerization production methods.

## 1. Introduction

Polyolefins possess excellent properties and are widely used across various applications. The development of innovative and efficient olefin polymerization catalysts has consistently been a focal point for academic and industrial research. Progress in olefin polymerization catalytic systems has accelerated the development of functionalized polyolefin materials [1,2]. Titanium and vanadium complexes demonstrate efficient and controllable performance in polymerization catalysis. Additionally, the ligands within these complexes are crucial for enhancing catalyst activity [3,4].

In 1995, Linden et al., synthesized titanium complexes utilizing binaphthoxy and bisphenoxy as ligands for olefin polymerization. The stereomodification of ligands influences the degree of polymerization and significantly impacts the stereoregularity of polyhexene [5]. In 2004, Fujita et al., synthesized a series of titanium complexes using 2-pyrimidine derivatives as ligands. They discovered that various ligands exhibit different catalytic activities in ethylene polymerization. Titanium catalysts can also be used to prepare block copolymers for ethylene/propylene copolymerization and monodisperse polyethylene with high molecular weight [6,7]. Li et al., reported titanium complexes using β-enaminoketonato as chelate ligands. These catalysts exhibit high activity after activation by the cocatalyst methylaluminoxane (MAO). The resulting ethylene polymers display narrow molecular weight distribution and high molecular weight characteristics. Additionally, they demonstrated that the spatial and electronic effects of the substituents influenced the polymerization yield, catalytic activity, and molecular weight [8]. Hu et al., reported a class of titanium complexes featuring tridentate ligands [O–N–P], which exhibited high activity and stability in ethylene polymerization after activation by MAO. The polymer produced was highly linear polyethylene with excellent copolymerization ability in ethylene/1-hexene and ethylene/norbornene copolymerizations [9]. Nomura et al., synthesized phenoxy monotitanocene complexes with various structures capable of copolymerizing ethylene and 1-butene (or 1-hexene). The study revealed that different substituents on cyclopentadienyl affect the catalytic polymerization activity. Minor changes in the ligand structure can cause significant alterations in the polymerization activity [10]. In 2016, Dong et al., demonstrated that bulky dibenzhydryl-substituted aryloxide semititanocene complexes can copolymerize ethylene with 1-hexene. When the ligands near Ti are relatively constrained, the catalytic activity decreases, particularly affecting larger comonomers that lack sufficient space for coordination to achieve polymerization [11]. Matthias et al., designed titanium catalysts based on phenol oxides, which regulate the coordination environment around the central metal by altering the substitutions at the phenol oxide coordination sites. Additionally, they introduced the ligand structure of imidazoline-2-imide to coordinate with titanium complexes, thereby resulting in improved stabilization of the central metal titanium [12]. In 2020, Zhao et al., synthesized titanium complexes with different-size substituents and investigated their propylene polymerization performances. The results indicated that the spatial and electronic effects of substituents considerably regulate the polymerization behavior, including activity and polymer molecular weight [13].

Li et al., developed trivalent vanadium complexes using pyridinedimide, pyridinamine imine, and pyridinediamine as chelating ligands. In the presence of cocatalysts, these catalysts exhibit high activity and high-temperature stability, thereby resulting in high relative molecular weight and single-peak distribution polyethylene [14,15,16]. Wu et al., synthesized novel trivalent vanadium complexes with single (double) salicylaldehyde imines as ligands. Under normal temperature and pressure, these vanadium complexes can efficiently catalyze ethylene polymerization, thereby producing linear polyethylene. Moreover, adjusting the ligand structure can effectively regulate the molecular weight of polyethylene [15,16]. Nomura et al., discovered that vanadium complexes with imines as ligands exhibit polymerization activity during ethylene polymerization, resulting in linear polyethylene with high molecular weight. Their studies also demonstrated that the activity of the catalyst is affected by the substituents on the imino aromatic ring of the ligand or the substituents on the phenoxy group [17,18]. Czaja et al., reported a series of vanadium chloride (IV) complexes chelated with bidentate and tetradentate salicylaldehyde imines. The results revealed that changing the substituents on salicylaldehyde imines impacted the ethylene polymerization activity [19,20]. Gambarotta et al., investigated the influence of ligand structure on ethylene polymerization catalyzed by vanadium complexes. By introducing different ligands to adjust the catalyst, ethylene/propylene copolymerization can be highly active in cyclohexane solvent [21].

In 2015, Trofymchuk et al., analyzed the ability of nickel catalysts with boron adducts for ethylene polymerization using density functional theory (DFT). The results revealed that adding boron adducts transformed the nickel catalysts into Lewis acids, activating them for ethylene polymerization [22]. In 2017, Johnson et al., prepared a palladium catalyst using B(C_6_F_5_)_3_ as a ligand. The results indicated that the remote combination of B(C_6_F_5_)_3_ enhanced the catalyst activity for ethylene polymerization [23].

**Scheme 1:**
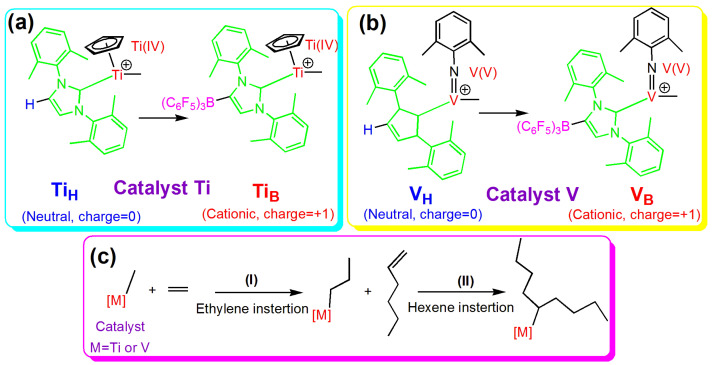
Schematic of the structure diagram of titanium (or vanadium) catalysts and the reaction pathway of ethylene/1-hexene copolymerization. (**a**) Structural diagram of titanium catalysts (**Ti_H_** and **Ti_B_**). (**b**) Structural diagram of vanadium catalysts (**V_H_** and **V_B_**). (**c**) Reaction pathway of ethylene (I) and 1-hexene (II) copolymerization.

Recently, Nomura et al., explored titanium (or vanadium) complexes with B(C_6_F_5_)_3_ as ligands. They discovered that titanium (or vanadium) catalysts exhibit high activity for ethylene/1-hexene copolymerization [24,25,26]; the ethylene polymerization catalytic activity reached 4590 kg-PE/mol-Ti·h [24] and 11,000 kg-PE/mol-V·h [25], respectively. However, the promotion mechanism of B(C_6_F_5_)_3_ as a ligand for titanium (or vanadium) catalysts in ethylene/1-hexene copolymerization remains unclear. Therefore, this study employed DFT calculations to investigate the promotion of B(C_6_F_5_)_3_ as a ligand for titanium (or vanadium) catalyst in ethylene/1-hexene copolymerization. A detailed mechanistic study can help understand the promotion mechanism of B(C_6_F_5_)_3_ as a ligand for olefin polymerization catalysts and design a new catalyst. Scheme 1 summarizes the structure diagram of titanium (or vanadium) catalysts and the reaction pathway of ethylene/1-hexene copolymerization [24,25].

## 2. Computational Methods

DFT calculations were performed using the Gaussian 16 software [27]. All structures were optimized and determined by frequency analysis to be a minimum (no imaginary frequency) or transition state (TS, with an imaginary frequency) at the *B3LYP*-*D3* (BJ) [28,29,30,31]/BSI level. BSI represents a basis set combining the SDD [32] for Ti, V, and 6–31 G (d) for nonmetal atoms. The pseudopotential basis set was employed for Ti and V atoms. The intrinsic reaction coordinate (IRC) [33] method was used to confirm transition states connecting reactants and products. The energetic results were refined at the M06-2X [34,35]/def2-TZVP level using the SMD [36] solvent effects model (with toluene as the solvent) through single-point energy calculations. The gas phase B3LYP/BSI harmonic frequency was employed to adjust the free energy using heat and entropy at 298.15 K (experimental temperature). Free energies derived at the M06-2X (SMD, solvent = toluene)/BSII level are discussed in the main text. The natural bond orbital (NBO) charges [37] and Wiberg bond indices (WBIs) were obtained at the B3LYP/BSI level. The reliability of the M06-2X/B3LYP combination in solving various transition metal catalytic reactions has been established [38,39,40,41,42,43,44,45,46,47].

The cubic files for the interaction region indicator (IRI) [48], electron localization function (ELF) [49,50], and global reactivity index (GRI) [51,52,53,54] analyses were implemented using the Multiwfn program 3.8 [55]. The results were visualized by the VMD program 1.9.3 [56].

## 3. Results and Discussion

### 3.1. Ethylene Insertion into Titanium (**Ti_H_** or **Ti_B_**) Catalysts

**Figure 1:**
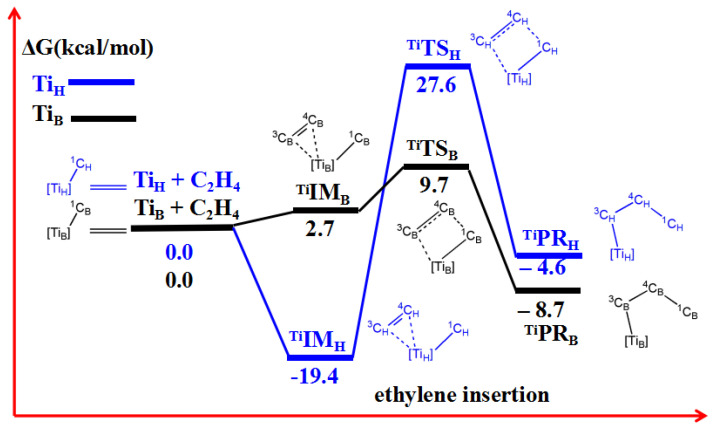
Reaction pathway of ethylene insertion into **Ti_H_** (blue) or **Ti_B_** (black) catalysts.

**Figure 2:**
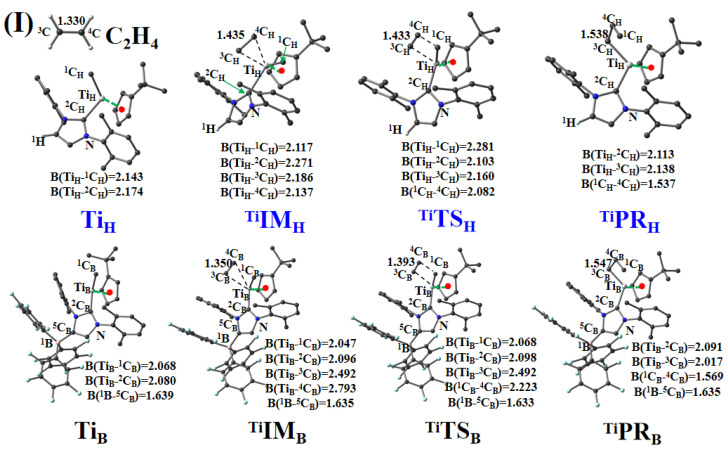

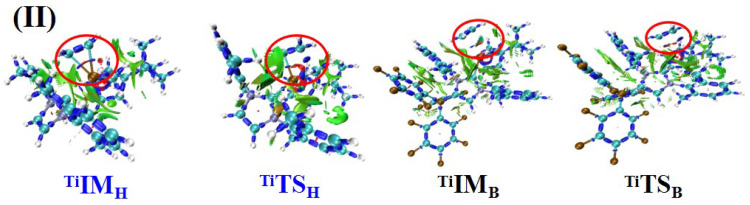
(**I**) Optimized structures of the reaction pathway for ethylene insertion into Ti catalysts (shown in Figure 1) with key bond lengths in angstroms. Most hydrogen atoms are omitted for clarity. (**II**) Interaction region indicator (IRI) analysis of **^Ti^IM_H_**_,_ **^Ti^TS_H_**, **^Ti^IM_B_**, and **^Ti^TS_B_**, with key interactions highlighted by red circles.

Figure 1 depicts the pathway of ethylene insertion to titanium (**Ti_H_** or **Ti_B_**) catalysts. The optimized structures and IRI analysis of key structures are shown in Figure 2. Table 1 presents the WBIs and natural charges (Q_NBO_) for certain key bonds and atoms. Evidently, the ethylene insertion to titanium (**Ti_H_** or **Ti_B_**) catalysts initially formed an intermediate (**^Ti^IM_H_** or **^Ti^IM_B_**), followed by a four-member ring transition state (**^Ti^TS_H_** or **^Ti^TS_B_**), ultimately generating the final product (**^Ti^PR_H_** or **^Ti^PR_B_**). **Ti_H_** and **Ti_B_** are half-titanocene catalysts containing N-heterocyclic carbene as a ligand, with the difference that in **Ti_B_**, one hydrogen of the N-heterocyclic carbene is substituted by B(C_6_F_5_)_3_ as the remote coordinating ligand. 

In **Ti_H_** catalysts, ethylene initially bonded to Ti, thereby forming intermediate **^Ti^IM_H_**. Owing to the coordination effect, the bond distance between Ti and the N-heterocyclic carbene (NHC) carbon atom (Ti_H_–^2^C_H_, 2.271 Å) in the **^Ti^IM_H_** structure was longer than that of **Ti_H_** (2.174 Å) by 0.097 Å. Additionally, the ^3^C_H_–^4^C_H_ (1.435 Å) bond was longer than that in the **C_2_H_4_** molecule (1.330 Å) by 0.105 Å. The Ti_H_–^3^C_H_ and Ti_H_–^4^C_H_ bond lengths were 2.186 and 2.137 Å, respectively, with large WBIs of Ti_H_–^3^C_H_ (0.646) and Ti_H_–^4^C_H_ (0.689). Compared with reactants (**Ti_H_ + C_2_H_4_**), the energy of **^Ti^IM_H_** was lower by 19.4 kcal/mol, thus indicating that **^Ti^IM_H_** is highly stable. After **^Ti^IM_H_**, the reaction reached a four-member ring transition state, **^Ti^TS_H_**, with the Ti_H_–^3^C_H_ bond (2.160 Å) being shorter than that in **^Ti^IM_H_**(2.186 Å) by 0.026 Å. The WBIs of Ti_H_–^3^C_H_ (0.572) and Ti_H_–^4^C_H_ (0.275) in **^Ti^TS_H_** were lower than those in **^Ti^IM_H_**. Following **^Ti^TS_H_**, the product of ethylene insertion, **^Ti^PR_H_** formed. The ^1^C_H_–^4^C_H_ (1.537 Å) and Ti_H_–^3^C_H_ (2.138 Å) bonds in **^Ti^PR_H_** were shorter than those in **^Ti^TS_H_** by 0.545 and 0.022 Å, respectively. Conversely, the ^3^C_H_–^4^C_H_ bond (1.538 Å) in **^Ti^PR_H_** was longer than that in **^Ti^TS_H_** by 0.105 Å.

During the reaction process, the WBIs of Ti_H_–^1^C_H_ (from 0.634 to 0.094) and ^3^C_H_–^4^C_H_ (from 2.039 to 1.012) bonds diminished from the reactant (**Ti_H_ + C_2_H_4_**) to the product **^Ti^PR_H_**. This decrease indicates the breakage of the Ti_H_–^1^C_H_ bond and the transition of the ^3^C_H_–^4^C_H_ bond from a double to single bond. Meanwhile, the WBI gradually increased from 0.009 to 1.011 for the ^1^C_H_–^4^C_H_ bond, thus indicating the formation of the ^1^C_H_–^4^C_H_ bond. Compared with the reactant (**Ti_H_ + C_2_H_4_**), the energy of **^Ti^TS_H_** was higher by 27.6 kcal/mol, whereas that of **^Ti^PR_H_** was lower by 4.6 kcal/mol. The intermediate **^Ti^IM_H_** was highly stable, and its energy was lower than that of the product **^Ti^PR_H_** by 14.8 kcal/mol. Consequently, the reaction was prone to halt at the intermediate stage, thus rendering difficulty in yielding the product.

In **Ti_B_** catalysts, one hydrogen of the N-heterocyclic carbene was substituted with B(C_6_F_5_)_3_ as the remote coordinating ligand. The Q_NBO_ value of the **Ti_B_** atom (1.487 e) in the **Ti_B_** catalyst was higher than that of the **Ti_H_** atom (1.206 e) in the **Ti_H_** catalyst. The process of ethylene insertion into **Ti_B_** catalysts was similar to that of the **Ti_H_** catalyst. The free energies demonstrate that ethylene insertion into the **Ti_B_** catalyst is thermodynamically and kinetically preferred over the **Ti_H_** catalyst. Compared with the reactant (**Ti_B_ + C_2_H_4_**), the energy barrier for **^Ti^TS_B_** was 9.7 kcal/mol, which is significantly lower than that for the **Ti_H_** catalyst (27.6 kcal/mol). Additionally, the reaction was exothermic by 8.7 kcal/mol, which is larger than the 4.6 kcal/mol for the **Ti_H_** catalyst. Unlike the highly stable **^Ti^IM_H_**, the energy of intermediate **^Ti^IM_B_** was slightly higher than that of the reactant by 2.7 kcal/mol. Further, the WBIs of Ti_B_–^3^C_B_ (0.270) and Ti_B_–^4^C_B_ (0.156) in **^Ti^IM_B_** were significantly lower than those (0.646 and 0.689) in **^Ti^IM_H_**, thus indicating that the reaction proceeded smoothly to yield the product.

**Scheme 2:**
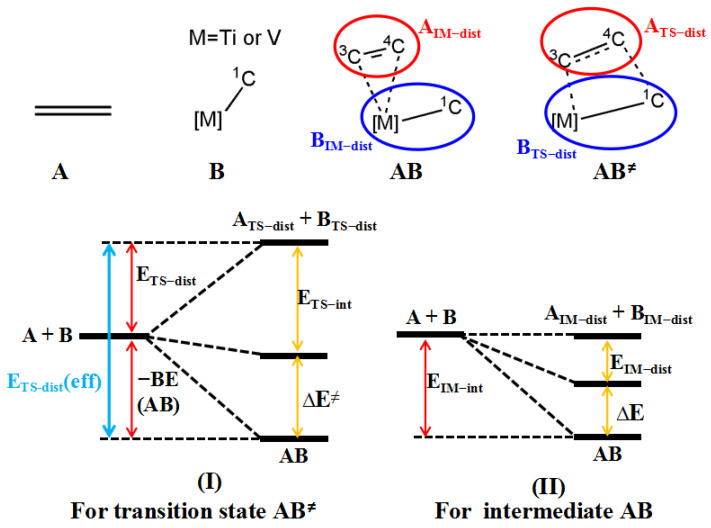
The scheme used to calculate the distortion and interaction energies between the **C_2_H_4_** (**A**) and M catalyst (**B**), (M = Ti or V).

Bickelhaupt’s activation strain analysis is frequently employed to gain insight into the origin of the various contributions [57,58,59]. This analysis decomposes the electronic activation energy ΔE^≠^ of the transition state into the distortion energy (E_TS−dist_) and interaction energy (E_TS−int_) between two reaction fragments (**A**: **C_2_H_4_** and **B**: **Ti** catalyst). Herein, this method was applied to analyze two transition states (**^Ti^TS_H_** and **^Ti^TS_B_**). As shown in Scheme 2I, the distortion energies were calculated as E_TS-dist_(A) = E_TS−A_ − E_A_ and E_TS−dist_(B) = E_TS−B_ − E_B_, where E_A_ (or E_B_) and E_TS−A_ (or E_TS−B_) are the energies of A (or B) in reactant ground state and transition state geometries, respectively. The interaction energies between A and B in the transition state were calculated by E_TS−int_ = ΔE^≠^ − E_TS−dist_ + BE(AB), where E_TS−dist_ = E_TS−dist_(A) + E_TS−dist_(B), BE(AB) is the binding energy between A and B in the intermediate complex AB, and ΔE^≠^ is the electronic activation energy for ethylene insertion reaction. When considering the intermediate complex AB as the reference, the electronic activation energy ΔE^≠^ = E_TS−dist_(eff) + E_TS−int_, so E_TS−dist_(eff)= E_TS−dist_ − BE(AB) represent the effective distortion energy. 

We also applied the same approach to decompose the two coordination energies of intermediates (**^Ti^IM_H_** and **^Ti^IM_B_**) into distortion energy (E_IM−dist_) and interaction energy (E_IM−int_) between two reaction fragments (A: **C_2_H_4_** and B: **Ti** catalyst). As shown in Scheme 2II, the distortion energies were calculated as E_IM−dist_(A) = E_IM−A_ − E_A_ and E_IM−dist_(B) = E_IM-B_ − E_B_, where E_A_ (or E_B_) and E_IM−A_ (or E_IM−B_) are the energies of A (or B) in reactant ground state and intermediate geometries, respectively. The interaction energies between A and B in the intermediates were calculated by E_IM−int_ = ΔE − E_IM−dist_, where E_IM−dist_ = E_IM−dist_(A) + E_IM−dist_(B), and ΔE is the binding energy between A and B in the intermediate complex AB. The results are presented in Table 2.

The E_IM−dist_ and E_TS−dist_(eff) values of **^Ti^IM_H_** and **^Ti^TS_H_** were 30.2 and 75.8 kcal/mol, respectively, which are much higher than those of **^Ti^IM_B_** (9.6 kcal/mol) and **^Ti^TS_B_** (36.1 kcal/mol), respectively. This indicates that **^Ti^IM_H_** and **^Ti^TS_H_** undergo a more significant configuration change compared to reactants. This is consistent with the IRI analysis. Figure 2II shows the IRI analysis of **^Ti^IM_H,_ ^Ti^TS_H_**, **^Ti^IM_B,_** and **^Ti^TS_B_** [48]. Evidently, the interactions between the two reaction fragments (**C_2_H_4_** and **Ti** catalyst) in **^Ti^IM_H_** and **^Ti^TS_H_** were stronger than those in **^Ti^IM_B_** and **^Ti^TS_B_**.

The *E*_IM−int_ of **^Ti^IM_H_** (−65.3 kcal/mol) was much lower than that of **^Ti^IM_B_** (−20.6 kcal/mol). This indicates that **^Ti^IM_H_** is more stable, and the reaction for the **Ti_H_** catalyst is prone to halt at the intermediate stage, whereas that for the **Ti_B_** catalyst proceeds smoothly to yield the product. The *E*_TS-int_ of **^Ti^TS_H_** (−64.2 kcal/mol) was lower than that of **^Ti^TS_B_** (−41.7 kcal/mol) by 22.5 kcal/mol. However, the E_TS−dist_(eff) (75.8 kcal/mol) of **^Ti^TS_H_** was much higher than that (36.1 kcal/mol) of **^Ti^TS_B_** by 39.7 kcal/mol. This explains why the energy barrier of **^Ti^TS_B_** is lower than **^Ti^TS_H_**, thus rendering the **Ti_B_** catalyst more efficient in catalyzing ethylene polymerization.

### 3.2. 1-Hexene Insertion into Titanium (**Ti_H_** or **Ti_B_**) Catalysts

**Figure 3:**
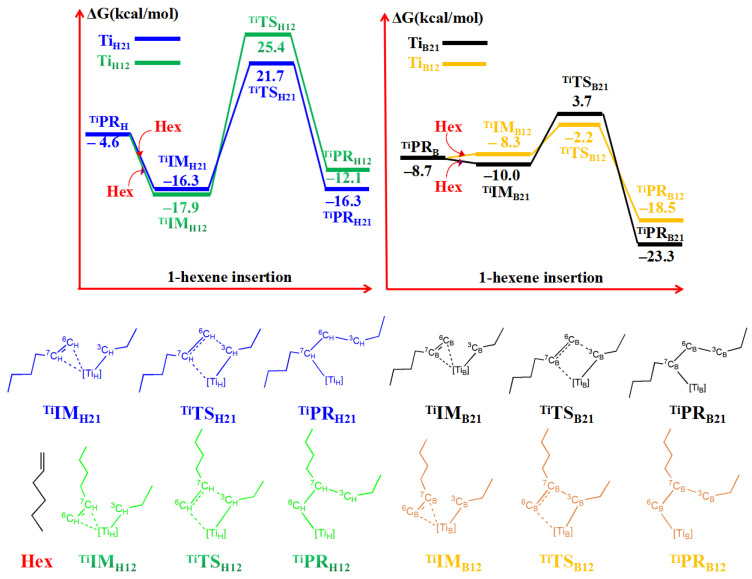
Reaction pathways for 1-hexene insertion into **Ti** catalysts: **Ti_H21_** (blue), **Ti_H12_** (green), **Ti_B21_** (black), and **Ti_B12_** (yellow). Depending on which carbon (^6^C or ^7^C) in 1-hexene forms a C–C bond with ^3^C carbon of [Ti]–alkyl, there are two insertion modes: the 1,2-insertion forming Ti-^6^C and ^7^C–^3^C bonds and the 2,1-insertion forming Ti-^7^C and ^6^C–^3^C bonds.

**Figure 4:**
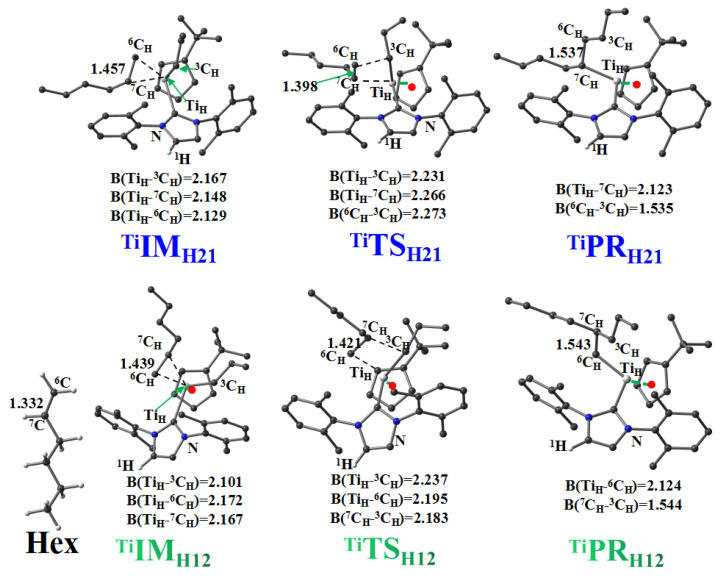

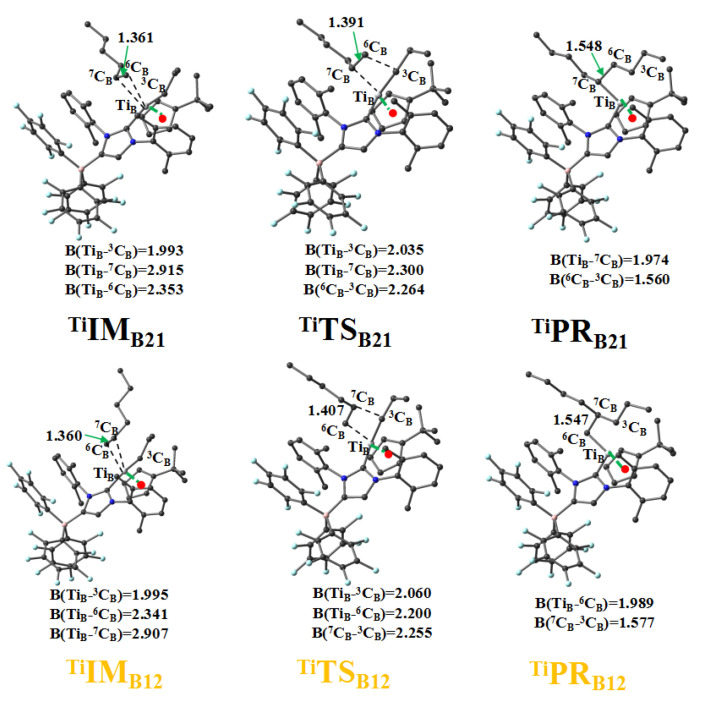
Optimized structures of the reaction pathway for 1-hexene insertion into **Ti** catalysts shown in Figure 3, with key bond lengths in angstroms. Most hydrogen atoms are omitted for clarity.

Following ethylene insertion into the **Ti_H_** or **Ti_B_** catalysts, the 1-hexene insertion reaction can be accomplished through 1,2 insertion or 2,1 insertion reactions. The 1-hexene 1,2 insertion and 2,1 insertion into the **Ti_H_** or **Ti_B_** catalysts involve an intermediate, followed by a four-member ring transition state, which yields the final product. Figure 3 illustrates the reaction pathways of 1-hexene insertion into Ti catalysts, namely **Ti_H21_** (blue), **Ti_H12_** (green), **Ti_B21_** (black), and **Ti_B12_** (yellow). Figure 4 shows the optimized structures of the 1-hexene insertion into Ti catalysts. The WBIs and Q_NBO_ involved in 1-hexene insertion into the **Ti_H_** and **Ti_B_** catalysts are presented in Table 3.

For **Ti_H_** catalysts, two pathways exist for 1-hexene insertion reactions: 1,2 insertion (**Ti_H12_**) or 2,1 insertion (**Ti_H21_**). The free energies indicated that the **Ti_H21_** pathway is thermodynamically and kinetically preferred over the **Ti_H12_** pathway. Compared with the reactant (**^Ti^PR_H_ + Hex**), the free energy barrier of **^Ti^TS_H21_** was 26.3 kcal/mol, which is lower than the 30.0 kcal/mol barrier for the **^Ti^TS_H12_** catalyst. Furthermore, the reaction for the **Ti_H21_** pathway was exothermic by 11.7 kcal/mol, which is higher than the 7.5 kcal/mol for the **Ti_H12_** pathway.

In the 1-hexene insertion **Ti_H21_** pathway, 1-hexene initially bonded to Ti, thereby forming the intermediate **^Ti^IM_H21_**. Owing to the coordination effect, the ^6^C_H_–^7^C_H_ bond (1.457 Å) was significantly longer than that in the 1-hexene (**Hex**) molecule (1.332 Å) by 0.125 Å. The Ti_H_–^6^C_H_ and Ti_H_–^7^C_H_ bond were 2.129 and 2.148 Å in length, respectively, with large WBIs of Ti_H_–^6^C_H_ (0.693) and Ti_H_–^7^C_H_ (0.677). Compared with reactants (**^Ti^PR_H_ + Hex**), the free energy of **^Ti^IM_H21_** was lower by 16.3 kcal/mol, thus indicating its stability. After **^Ti^IM_H21_**, the reaction reached a four-member ring transition state, **^Ti^TS_H21_**, with the Ti_H_–^7^C_H_ (2.266 Å) being longer than that in **^Ti^IM_H21_** (2.148 Å) by 0.118 Å. The WBIs of Ti_H_–^6^C_H_ (0.192) and Ti_H_–^7^C_H_ (0.393) in **^Ti^TS_H21_** were lower than those in **^Ti^IM_H21_**. Following **^Ti^TS_H21_**, the product of 1-hexene insertion, **^Ti^PR_H21_**, formed. Compared with the **^Ti^TS_H21_** configuration, the ^3^C_H_–^6^C_H_ (1.535 Å) and Ti_H_–^7^C_H_ (2.123 Å) bonds in **^Ti^PR_H21_** were shorter, whereas the ^6^C_H_–^7^C_H_ bond (1.537 Å) in **^Ti^PR_H21_** was longer.

During the reaction process, the WBIs of the Ti_H_–^3^C_H_ (from 0.627 to 0.100) and ^6^C_H_–^7^C_H_ bonds (from 1.980 to 0.999) decreased from the reactant (**^Ti^PR_H_ + Hex**) to the product **^Ti^PR_H21_**. This decrease indicates the breakage of the Ti_H_–^3^C_H_ bond and the transition of the ^6^C_H_–^7^C_H_ bond from a double to single bond. Meanwhile, the WBI for the ^3^C_H_–^6^C_H_ bond gradually increased from 0.012 to 1.000, thus indicating the formation of the ^3^C_H_–^6^C_H_ bond. Similar to the ethylene insertion reaction, the 1-hexene insertion intermediate **^Ti^IM_H21_** was stable, with its energy being equal to that of the product **^Ti^PR_H21_**.

For **Ti_B_** catalysts, 1,2 insertion (**Ti_B12_**) or 2,1 insertion (**Ti_B21_**) pathways exist for 1-hexene insertion reactions. The free energy results revealed that the **Ti_B21_** pathway is thermodynamically preferred over the **Ti_B12_** pathway. Compared with the reactant (**^Ti^PR_B_ + Hex**), the free energy barrier of **^Ti^TS_B21_** was 12.4 kcal/mol, which is higher than the 6.5 kcal/mol for the **^Ti^TS_B12_** catalyst. Additionally, the reaction for the **Ti_B21_** pathway was exothermic by 14.6 kcal/mol, significantly larger than the 9.8 kcal/mol for the **Ti_B12_** pathway. The energies of both transition states (**^Ti^TS_B21_** and **^Ti^TS_B12_**) were not high, thus allowing them to be easily crossed during the reaction. Consequently, the product (**^Ti^PR_B21_**) with lower product energy **^Ti^PR_B21_** became the main reaction product.

Comparison between **Ti_H_** and **Ti_B_** catalysts in the 1-hexene insertion reaction revealed that both catalysts followed the dominant 2,1 insertion pathway (**Ti_H21_** and **Ti_B21_**). The free energy barrier for **Ti_B21_** was 12.4 kcal/mol, significantly lower than 26.3 kcal/mol for **Ti_H21_**. Unlike the highly stable **^Ti^IM_H21_**, the energy of the intermediate **^Ti^IM_B21_** was 13.3 kcal/mol higher than that of the product **^Ti^PR_B21_**. The WBIs of the Ti_B_–^6^C_B_ (0.341) and Ti_B_–^7^C_B_ (0.120) in **^Ti^IM_B21_** were considerably lower than those in **^Ti^IM_H21_** (0.693 and 0.677). Therefore, the 1-hexene insertion reaction for **Ti_B21_** was easier to perform.

**Figure 5:**
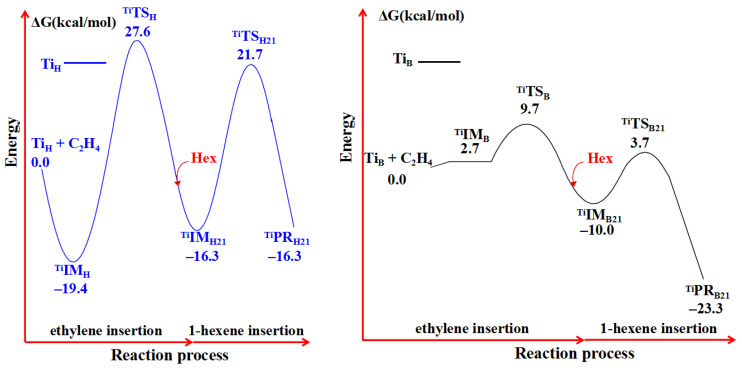
Comparison of the entire ethylene and 1-hexene insertion reaction process for **Ti_H_** (blue) and **Ti_B_** (black) catalysts.

The comparison of the entire ethylene and 1-hexene insertion reaction process for **Ti_H_** and **Ti_B_** catalysts is shown in Figure 5. In the ethylene and 1-hexene insertion into **Ti_H_** catalysts, the energy of the transition state **^Ti^TS_H_** was the highest at 27.6 kcal/mol, whereas that of the final product **^Ti^PR_H21_** was −16.3 kcal/mol. However, the energy of the intermediate **^Ti^IM_H_** was −19.4 kcal/mol, lower than that of the final product **^Ti^PR_H21_** (−16.3 kcal/mol)**_,_** thus indicating that the intermediate is highly stable. Therefore, the reaction was prone to halt at the intermediate rather than proceed to yield the product. Conversely, for the entire ethylene and 1-hexene insertion into **Ti_B_** catalysts, compared with the reactant, the energy of the transition state **^Ti^TS_B_** was 9.7 kcal/mol, considerably lower than that of **^Ti^TS_H_** (27.6 kcal/mol). The energy of the final product **^Ti^PR_B21_** was −23.3 kcal/mol, significantly lower than that of **^Ti^PR_H21_** (−16.3 kcal/mol). Consequently, the entire ethylene and 1-hexene insertion reaction proceeded smoothly to reach the final product for the **Ti_B_** catalyst.

### 3.3. Ethylene and 1-Hexene Insertion into V Catalysts

**Figure 6:**
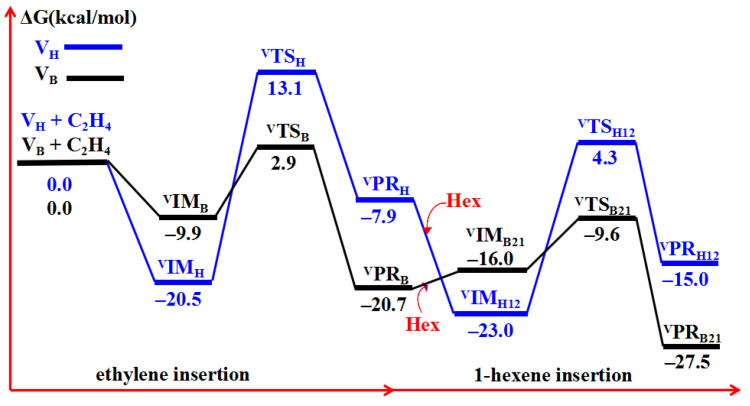

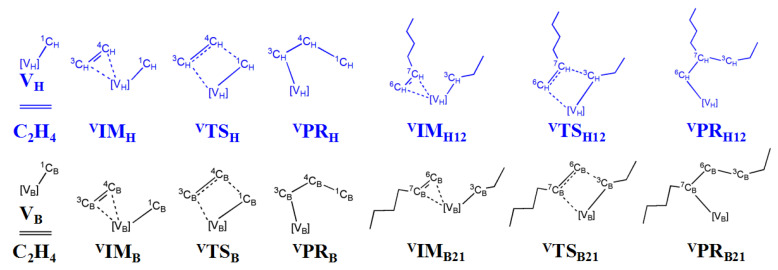
Reaction pathways for ethylene and 1-hexene insertion into **V_H_** (blue) or **V_B_** (black) catalysts. Depending on which carbon (^6^C or ^7^C) in 1-hexene forms a C–C bond with ^3^C carbon of [V]–alkyl, there are two insertion modes for 1-hexene insertion: the 1,2-insertion forming V–^6^C and ^7^C–^3^C bonds and the 2,1-insertion forming V–^7^C and ^6^C–^3^C bonds.

**Figure 7:**
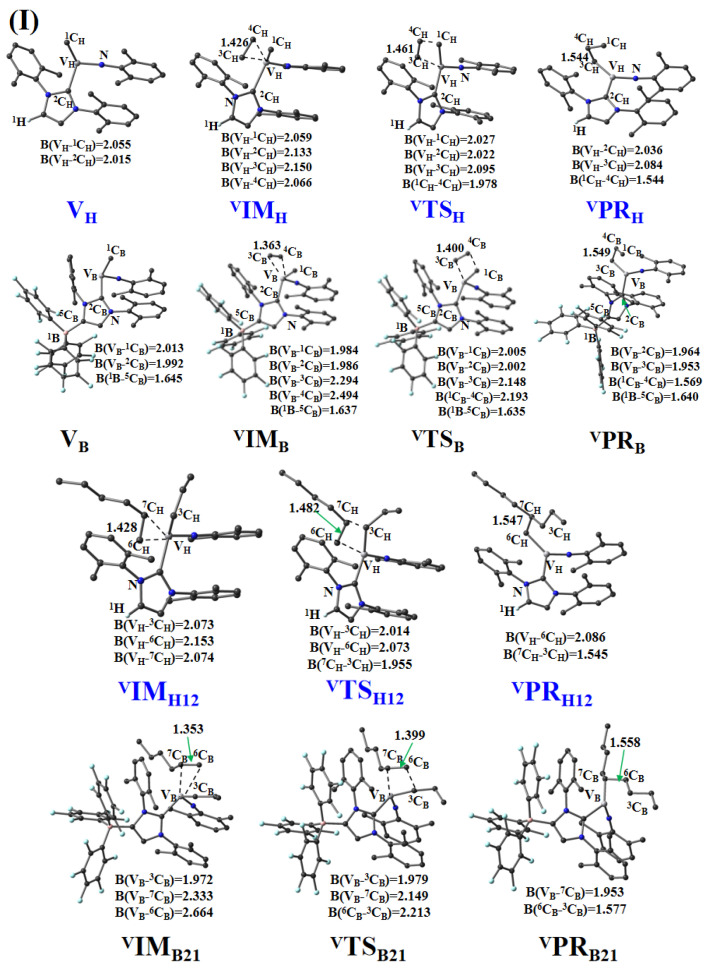

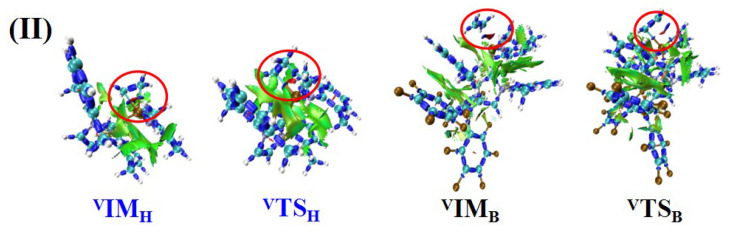
(**I**) Optimized structures of ethylene and 1-hexene insertion into **V** catalysts reaction pathway shown in Figure 6, with key bond lengths in angstroms. Most hydrogen atoms are omitted for clarity. (**II**) Interaction region indicator (IRI) analysis of **^V^IM_H_, ^V^TS_H_**, **^V^IM_B_**, and **^V^TS_B_**, with key interactions highlighted by red circles.

Figure 6 shows the reaction pathways for ethylene and 1-hexene insertion into vanadium (**V_H_** or **V_B_**) catalysts. The key optimized structures and IRI analysis of important structures are shown in Figure 7. The WBIs and Q_NBO_ involved in the ethylene and 1-hexene insertion into **V_H_** or **V_B_** catalysts are presented in Table 4. Similar to the reaction pathway catalyzed by the Ti catalyst, the ethylene and 1-hexene insertion into **V_H_** or **V_B_** catalysts involved the formation of an intermediate, followed by a four-member ring transition state, thereby resulting in the final product. Both the **V_H_** and **V_B_** catalysts contain N-heterocyclic carbene as a ligand, with the difference that in the **V_B_** catalyst, one hydrogen of the N-heterocyclic carbene is substituted with B(C_6_F_5_)_3_ as the remote coordinating ligand. The Q_NBO_ value for the V_B_ atom (0.767 e) in the **V_B_** catalyst was larger than that for the V_H_ atom (0.435 e) in the **V_H_** catalyst.

For ethylene insertion into vanadium (**V_H_** and **V_B_**) catalysts, the energy results indicate that ethylene insertion into the **V_B_** catalyst is thermodynamically and kinetically preferred over the **V_H_** catalyst. Compared with the reactant, the energy barrier for **V_B_** ethylene insertion was 2.9 kcal/mol, significantly lower than the 13.1 kcal/mol for **V_H_** ethylene insertion. The reaction was exothermic by 20.7 kcal/mol for the **V_B_** catalyst, larger than the 7.9 kcal/mol for the **V_H_** catalyst. The intermediate **^V^IM_H_** was highly stable, with its energy being lower than that of the product **^V^PR_H_** by 12.6 kcal/mol. Therefore, the reaction was prone to halt at the intermediate stage, thus rendering difficulty in yielding the product. However, the energy of the intermediate **^V^IM_B_** was higher than that of the product **^V^PR_B_** by 10.8 kcal/mol. Additionally, the WBIs of V_B_–^3^C_B_ (0.384) and V_B_–^4^C_B_ (0.200) in **^V^IM_B_** were significantly lower than those in **^V^IM_H_** (0.621 and 0.712). Consequently, the reaction proceeded smoothly to yield the product for the **V_B_** ethylene insertion.

The results for the decomposition of activation energies of the transition states (**^V^TS_H_** and **^V^TS_B_**) and coordination energies of intermediates (**^V^IM_H_** and **^V^IM_B_**) into the distort energies and the interaction energies are presented in Table 5.

The E_IM−dist_ and E_TS−dist_(eff) values of **^V^IM_H_** and **^V^TS_H_** were 15.4 and 72.7 kcal/mol, respectively, higher than those of **^V^IM_B_** (5.4 kcal/mol) and **^V^TS_B_** (48.5 kcal/mol), respectively. This indicates that **^V^IM_H_** and **^V^TS_H_** underwent a more significant configuration change compared with the reactants, which is consistent with the IRI analysis. Figure 7II shows the IRI analysis of **^V^IM_H,_ ^V^TS_H_**, **^V^IM_B,_** and **^V^TS_B_** [48]. The interactions between the two reaction fragments (**C_2_H_4_** and **V** catalyst) in **^V^IM_H_** and **^V^TS_H_** were stronger than those in **^V^IM_B_** and **^V^TS_B_**.

The E_IM−int_ of **^V^IM_H_** (−49.5 kcal/mol) was considerably lower than that of **^V^IM_B_** (−28.0 kcal/mol). This suggests that the intermediate **^V^IM_H_** was more stable, and the ethylene insertion reaction for the **V_H_** catalyst was prone to halt at the intermediate. Conversely, for the **V_B_** catalyst, the ethylene insertion reaction proceeded smoothly to yield the product. The E_TS−int_ of **^V^TS_H_** (−73.7 kcal/mol) was lower than that of **^v^TS_B_** (−59.5 kcal/mol) by 14.2 kcal/mol. However, the E_TS−dist_(eff) (72.7 kcal/mol) of **^v^TS_H_** was significantly larger than that of **^v^TS_B_** (48.5 kcal/mol) by 24.2 kcal/mol. This explains why the energy barrier of **^V^TS_B_** was lower than that of **^V^TS_H_**, thus rendering the **V_B_** catalyst more efficient in catalyzing ethylene polymerization. After ethylene insertion into **V_H_** and **V_B_** catalysts, the subsequent 1-hexene insertion reaction occurred through 1,2 insertion or 2,1 insertion reactions. The 1-hexene 1,2 insertion and 2,1 insertion into **V_H_** and **V_B_** catalysts involve an intermediate, followed by a four-member ring transition state, leading to the final product. For clarity, this study focused on the optimal reaction pathways (**V_H12_** and **V_B21_**), and the detailed reaction pathways are shown in Figures S1 and S2. **V_H12_** and **V_B21_** are the dominant 1-hexene insertion reaction pathways for **V_H_** and **V_B_** catalysts, respectively.

The energy results indicate that the **V_B21_** pathway is preferred over the **V_H12_** pathway. As shown in Figure 6, the energy of the transition state **^V^TS_B21_** was lower than that of **^V^TS_H12_** by 13.9 kcal/mol. Moreover, the energy of product **^V^PR_B21_** was lower than that of **^V^PR_H12_** by 12.5 kcal/mol. Furthermore, the energy of **^V^IM_H12_** was lower than that of the corresponding product **^V^PR_H12_** by 8.0 kcal/mol, thus indicating its high stability. Therefore, the reaction was prone to halt at the intermediate rather than proceed to yield the product. However, the energy of intermediate **^V^IM_B21_** was higher than that of the product **^V^PR_B21_** by 11.5 kcal/mol. The WBIs of V_B_–^6^C_B_ (0.136) and V_B_–^7^C_B_ (0.257) in **^V^IM_B21_** were significantly lower than those in **^V^IM_H12_** (0.660 and 0.601). Thus, the reaction proceeded smoothly to yield the product for **V_B_** 1-hexene insertion.

In the insertion of ethylene and 1-hexene into **V_H_** catalysts, the transition state energy (**^V^TS_H_**) was 13.1 kcal/mol, whereas that of the final product energy **^V^PR_H12_** was −15.0 kcal/mol. However, the energies of intermediates **^V^IM_H_** and **^V^IM_H12_** were −20.5 and −23.0 kcal/mol, respectively, lower than that of the final product **^V^PR_H12_** (−15.0 kcal/mol). This indicates that the intermediate stages were highly stable, thus rendering the reaction prone to stopping at the intermediate stages and difficult to yield the final product. Conversely, for the ethylene and 1-hexene insertion into **V_B_** catalysts, the transition state energy **^V^TS_B_** was significantly lower at 2.9 kcal/mol compared with **^V^TS_H_** (13.1 kcal/mol). The energy of the final product **^V^PR_B21_** was −27.5 kcal/mol, substantially lower than that of **^V^PR_H12_** (−15.0 kcal/mol). Consequently, the ethylene and 1-hexene insertion reaction proceeded smoothly and yielded the final product when using the **V_B_** catalyst.

Further comparison between **Ti_B_** and **V_B_** catalysts revealed that the energy of transition state **^V^TS_B_** of the ethylene and 1-hexene insertion into **V_B_** catalysts was 2.9 kcal/mol, which is lower than that of **^Ti^TS_B_** (9.7 kcal/mol). Additionally, the energy of the final product **^V^PR_B21_** was −27.5 kcal/mol, which is lower than that of **^Ti^PR_B21_** (−23.3 kcal/mol). These results indicate that the **V_B_** catalyst has higher reaction activity than the **Ti_B_** catalyst, which is in agreement with the experimental results. Further, the catalytic activity reached 4590 kg-PE/mol-Ti·h and 11,000 kg-PE/mol-V·h for ethylene polymerization [24,25].

**Figure 8:**
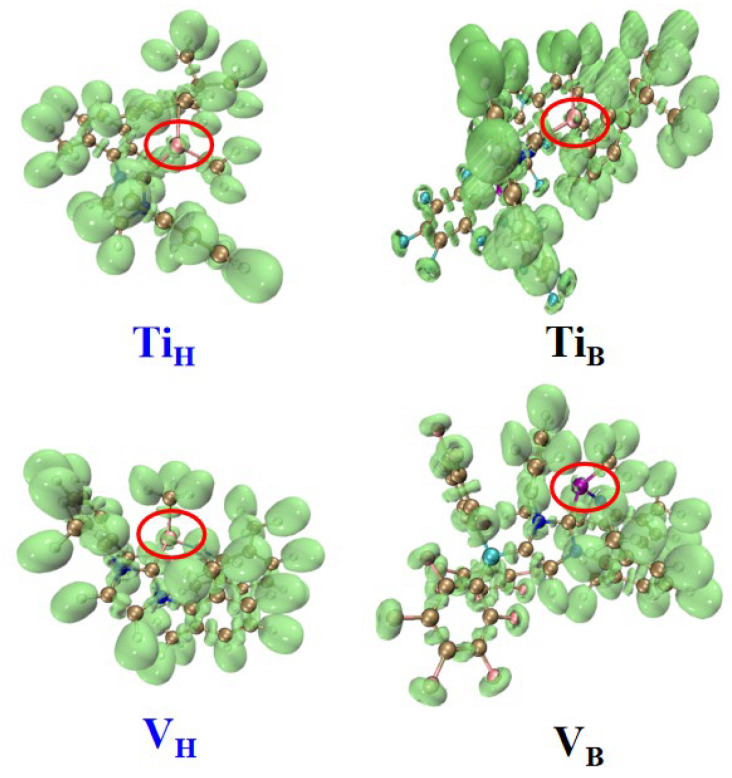
Isosurface maps of electron localization function for **Ti_H_**, **Ti_B_**, **V_H_**, and **V_B_**.

To thoroughly investigate the reaction, both the GRI and ELF analyses were conducted on key molecules (Table 6 and Figure 8). The **V_B_** and **Ti_B_** catalysts exhibited the largest electrophilicity (*ω*) values, 9.449 and 7.478, respectively, thus signifying their electrophilic nature. By contrast, **V_H_** and **Ti_H_** exhibited substantial global nucleophilicity (*N*_Nu_) values of 5.650 and 6.356, thus indicating that they are nucleophilic. The **C_2_H_4_** and **Hex** molecules displayed moderate *N*_Nu_ values of 1.863 and 2.361, thus suggesting a certain level of nucleophilicity. Thus, **V_B_** and **Ti_B_** catalysts can facilitate olefin polymerization reactions, whereas **V_H_** and **Ti_H_** do not. The *ω* value of **V_B_** (9.449) was slightly higher than that of **Ti_B_** (7.478); thus, **V_B_** exhibited greater electrophilicity and reactivity, which is in agreement with the experimental results [24,25].

Figure 8 presents the ELF analysis for key molecules, including **Ti_H_**, **Ti_B_**, **V_H_**, and **V_B_**. The ELF analysis, which is typically used to analyze nucleophilic attack sites, was employed herein to obtain the ELF isosurface maps for these molecules (Figure 8). A comparison of the ELF isosurface maps for **Ti_H_** and **V_H_** with those of **Ti_B_** and **V_B_** revealed that the ELF values in the atomic regions of **Ti_B_** and **V_B_** were lower. This suggests that **Ti_B_** and **V_B_** atoms are more susceptible to nucleophilic attacks. These observations align with the conclusions drawn from previous discussions.

## 4. Conclusions

DFT calculations were employed to study the promotion of B(C_6_F_5_)_3_ as a ligand for titanium (or vanadium) catalysts in the copolymerization of ethylene and 1-hexene. The results revealed that: (I) Ethylene insertion into **Ti_B_** was preferred over **Ti_H_**, both thermodynamically and kinetically. The intermediate **^Ti^IM_H_** was highly stable, with its energy even lower than that of the product **^Ti^PR_H_**, thus it was easy for the reaction to stall at the intermediate stage and difficult to reach the product. In contrast to the very stable **^Ti^IM_H_**, the energy of the intermediate **^Ti^IM_B_** was slightly higher than the reactant by 2.7 kcal/mol, thereby allowing the reaction to proceed smoothly to reach the product. (II) For both **Ti_H_** and **Ti_B_** catalysts, the 2,1 insertion reaction (**Ti_H21_** and **Ti_B21_**) was the dominant reaction pathway for 1-hexene insertion. Moreover, the 1-hexene insertion reaction for **Ti_B21_** was preferred over **Ti_H21_** and was easier to perform. The entire ethylene and 1-hexene insertion reaction proceeded smoothly to reach the final product for the **Ti_B_** catalyst. (III) Similar to the case of **Ti** catalysts, **V_B_** was preferred over **V_H_** for the entire ethylene and 1-hexene insertion reaction. Furthermore, the **V_B_** catalyst exhibited higher reaction activity than the **Ti_B_** catalyst, which agrees with the experimental results. The ELF and GRI analyses also indicated that titanium (or vanadium) catalysts with B(C_6_F_5_)_3_ as a ligand demonstrated higher reactivity. This study is expected to enhance the understanding of the promotion of B(C_6_F_5_)_3_ as a ligand for titanium (or vanadium) catalysts, aid in developing new strategies for polymerization catalyst design, and lead to more cost-effective polymerization production methods.

## Figures and Tables

**Table 1:** WBIs and Q_NBO_ for some important bonds and atoms for ethylene insertion into Ti catalysts.

	WBI					Q_NBO_ (e)	
	B (Ti_H_–^1^C_H_)	B (Ti_H_–^3^C_H_)	B (Ti_H_–^4^C_H_)	B (^3^C_H_–^4^C_H_)	B (^1^C_H_–^4^C_H_)	Ti_H_	^1^H
**Ti_H_ + C_2_H_4_**	0.634			2.039		1.206	0.251
**^Ti^** **IM_H_**	0.797	0.646	0.689	1.211	0.009	1.113	0.254
**^Ti^** **TS_H_**	0.464	0.572	0.275	1.263	0.473	1.056	0.248
**^Ti^** **PR_H_**	0.094	0.627	0.042	1.012	1.011	1.142	0.248
	B (Ti_B_–^1^C_B_)	B (Ti_B_–^3^C_B_)	B (Ti_B_–^4^C_B_)	B (^3^C_B_–^4^C_B_)	B (^1^C_B_–^4^C_B_)	Ti_B_	B(C_6_F_5_)_3_
**Ti_B_ + C_2_H_4_**	0.861			2.039		1.487	−0.497
**^Ti^** **IM_B_**	0.882	0.270	0.156	1.799	0.004	1.460	−0.518
**^Ti^** **TS_B_**	0.743	0.522	0.171	1.473	0.351	1.233	−0.524
**^Ti^** **PR_B_**	0.252	0.890	0.122	0.978	0.967	1.344	−0.518

**Table 2:** The decomposition of activation energies of the transition states (**^Ti^TS_H_** and **^Ti^TS_B_**) and coordination energies of intermediates (**^Ti^IM_H_** and **^Ti^IM_B_**) and into the distort energies and the interaction energies, given in kcal/mol.

Complex	Δ*G*^≠^	ΔE^≠^	BE(AB)	E_TS−dist_(A)	E_TS−dist_(B)	E_TS−dist_	E_TS−dist_(eff)	E_TS−int_
**^Ti^** **TS_H_**	27.6	11.6	−35.1	21.8	18.9	40.7	75.8	−64.2
**^Ti^** **TS_B_**	9.7	−5.6	−11.0	9.5	15.6	25.1	36.1	−41.7
Complex	Δ*G*	ΔE		E_IM−dist_(A)	E_IM−dist_(B)		E_IM−dist_	E_IM−int_
**^Ti^** **IM_H_**	−19.4	−35.1		18.4	11.8		30.2	−65.3
**^Ti^** **IM_B_**	2.7	−11.0		1.2	8.4		9.6	−20.6

**Table 3:** WBIs and Q_NBO_ for some important bonds and atoms for the 1-hexene insertion into Ti catalysts.

	WBI					Q_NBO_ (e)	
	B (Ti_H_−^3^C_H_)	B (Ti_H_−^7^C_H_)	B (Ti_H_−^6^C_H_)	B (^6^C_H_−^7^C_H_)	B (^3^C_H_−^6^C_H_)	Ti_H_	^1^H
**^Ti^** **PR_H_ + Hex**	0.627			1.980		1.142	0.248
**^Ti^** **IM_H21_**	0.654	0.677	0.693	1.132	0.012	1.325	0.256
**^Ti^** **TS_H21_**	0.528	0.393	0.192	1.431	0.368	0.844	0.248
**^Ti^** **PR_H21_**	0.100	0.588	0.048	0.999	1.000	1.154	0.248
	B (Ti_H_−^3^C_H_)	B (Ti_H_−^6^C_H_)	B (Ti_H_−^7^C_H_)	B (^6^C_H_−^7^C_H_)	B (^3^C_H_−^7^C_H_)	Ti_H_	^1^H
**^Ti^** **IM_H12_**	0.753	0.648	0.636	1.180	0.009	1.204	0.255
**^Ti^** **TS_H12_**	0.485	0.506	0.194	1.325	0.431	1.047	0.247
**^Ti^** **PR_H12_**	0.103	0.625	0.038	0.996	0.986	1.119	0.248
	B (Ti_B_−^3^C_B_)	B (Ti_B_−^7^C_B_)	B (Ti_B_−^6^C_B_)	B (^6^C_B_−^7^C_B_)	B (^3^C_B_−^6^C_B_)	Ti_B_	B(C_6_F_5_)_3_
**^Ti^** **PR_B_ + Hex**	0.890			1.980		1.344	−0.518
**^Ti^** **IM_B21_**	0.936	0.120	0.341	1.687	0.002	1.395	−0.533
**^Ti^** **TS_B21_**	0.764	0.475	0.193	1.463	0.332	1.273	−0.534
**^Ti^** **PR_B21_**	0.234	0.893	0.116	0.962	0.968	1.401	−0.528
	B (Ti_B_−^3^C_B_)	B (Ti_B_−^6^C_B_)	B (Ti_B_−^7^C_B_)	B (^6^C_B_−^7^C_B_)	B (^3^C_B_−^7^C_B_)	Ti_B_	B(C_6_F_5_)_3_
**^Ti^** **IM_B12_**	0.921	0.334	0.120	1.691	0.003	1.432	−0.533
**^Ti^** **TS_B12_**	0.723	0.567	0.152	1.383	0.361	1.296	−0.533
**^Ti^** **PR_B12_**	0.251	0.900	0.097	0.968	0.949	1.414	−0.525

**Table 4:** WBIs and Q_NBO_ for some important bonds and atoms for the ethylene and 1-hexene insertion into **V** catalysts.

	WBI					Q_NBO_ (e)	
	B (V_H_–^1^C_H_)	B (V_H_–^3^C_H_)	B (V_H_–^4^C_H_)	B (^3^C_H_–^4^C_H_)	B (^1^C_H_–^4^C_H_)	V_H_	^1^H
**V_H_ + C_2_H_4_**	0.830			2.039		0.435	0.250
**^V^** **IM_H_**	0.841	0.621	0.712	1.274	0.015	0.555	0.254
**^V^** **TS_H_**	0.660	0.686	0.260	1.189	0.559	0.179	0.247
**^V^** **PR_H_**	0.172	0.705	0.039	1.010	1.007	0.635	0.250
	B (V_H_–^3^C_H_)	B (V_H_–^7^C_H_)	B (V_H_–^6^C_H_)	B (^6^C_H_–^7^C_H_)	B (^3^C_H_–^6^C_H_)	V_H_	^1^H
**^V^** **PR_H_ + Hex**	0.705			1.980		0.635	0.250
**^V^** **IM_H12_**	0.757	0.601	0.660	1.242	0.014	0.807	0.254
**^V^** **TS_H12_**	0.639	0.692	0.256	1.119	0.581	0.214	0.248
**^V^** **PR_H12_**	0.138	0.671	0.029	0.998	0.990	0.734	0.250
	B (V_B_–^1^C_B_)	B (V_B_–^3^C_B_)	B (V_B_–^4^C_B_)	B (^3^C_B_–^4^C_B_)	B (^1^C_B_–^4^C_B_)	V_B_	B(C_6_F_5_)_3_
**V_B_ + C_2_H_4_**	0.982			2.039		0.767	−0.482
**^V^** **IM_B_**	1.018	0.384	0.200	1.692	0.004	0.911	−0.519
**^V^** **TS_B_**	0.861	0.596	0.162	1.440	0.355	0.596	−0.528
**^V^** **PR_B_**	0.266	0.974	0.132	0.966	0.960	0.692	−0.517
	B (V_B_–^3^C_B_)	B (V_B_–^7^C_B_)	B (V_B_–^6^C_B_)	B (^6^C_B_–^7^C_B_)	B (^3^C_B_–^6^C_B_)	V_B_	B(C_6_F_5_)_3_
**^V^** **PR_B_ + Hex**	0.974			1.980		0.692	−0.517
**^V^** **IM_B21_**	0.978	0.257	0.136	1.750	0.033	0.947	−0.529
**^V^** **TS_B21_**	0.868	0.545	0.167	1.427	0.351	0.679	−0.535
**^V^** **PR_B21_**	0.334	0.937	0.157	0.944	0.935	0.752	−0.526

**Table 5:** The decomposition of activation energies of the transition states (**^V^TS_H_** and **^V^TS_B_**) and coordination energies of intermediates (**^V^IM_H_** and **^V^IM_B_**) and into the distort energies and the interaction energies, given in kcal/mol.

Complex	Δ*G*^≠^	ΔE^≠^	BE(AB)	E_TS−dist_(A)	E_TS−dist_(B)	E_TS−dist_	E_TS−dist_(eff)	*E*_TS−int_
**^V^** **TS_H_**	13.1	−1.0	−34.1	28.1	10.5	38.6	72.7	−73.7
**^V^** **TS_B_**	2.9	−11.0	−22.6	9.8	16.1	25.9	48.5	−59.5
Complex	Δ*G*	ΔE		E_IM−dist_(A)	E_IM−dist_(B)		E_IM−dist_	E_IM−int_
**^V^** **IM_H_**	−20.5	−34.1		14.4	1.0		15.4	−49.5
**^V^** **IM_B_**	−9.9	−22.6		2.0	3.4		5.4	−28.0

**Table 6:** The global reactivity index for some key molecules.

	η ^a^	μ ^b^	ω ^c^	N_Nu_ ^d^
**Ti_H_**	3.930	−1.855	0.302	6.356
**Ti_B_**	3.823	−7.794	7.478	0.857
**V_H_**	4.318	−2.083	0.421	5.650
**V_B_**	3.334	−8.066	9.449	0.811
**C_2_H_4_**	13.714	−3.508	0.592	1.863
**Hex**	12.307	−3.053	0.619	2.361

^a^ Chemical hardness (η, in eV). ^b^ Electronic chemical potential (μ, in eV). ^c^ Global electrophilicity (ω, in eV). ^d^ Global nucleophilicity (N_Nu_, in eV).

## Data Availability

The data presented in this study are available in the Supplementary Materials.

## Supplementary Materials

The following supporting information can be downloaded at: https://www.mdpi.com/article/10.3390/polym15112435/s1. Figure S1. The details pathway of 1-hexene insertion into V catalysts, VH12 (blue), VH21 (green), VB21 (black), and VB12 (yellow). Figure S2. Optimized structures of 1-hexene insertion into V catalysts reaction pathway shown in Figure S1. Table S1: Additional computational details. Table S2: Energies and Cartesian coordinates of all the optimized structures.
