# Supplementary material for: Promotion of B(C6F5)3 as Ligand for Titanium (or Vanadium) Catalysts in the Copolymerization of Ethylene and 1-Hexene: A Computational Study
Source: Polymers (Basel). 2023 May 24;15(11):2435. doi: 10.3390/polym15112435 (PMC10255729; doi:10.3390/polym15112435)
Supplement: Supplementary file 1 [file polymers-15-02435-s001.zip › polymers-2367563-supplementary.pdf]

Supplementary Materials

The Promotion of $B(C_6F_5)_3$ as a Ligand for Titanium (or Vanadium) Catalyst in the Copolymerization of Ethylene and 1-Hexene: A Computational Study

Shuyuan Yu [1], Chenggen Zhang [1,*], Fei Wang [1], Xinru Liang [1], Mengyao Yang[1], Mengyu An[1]

[1] College of Chemistry and Material Science, Langfang Normal University, Langfang 065000, China; yushuyuan05@mails.ucas.ac.cn (S.Y.); wangfei20220726@163.com (F.W.); liangxinru2023@163.com (X.L.); yangmengyao2023@163.com (M.Y.); anmengyu1466850030@163.com (M.A.)

* Correspondence: chgzhang04@mails.ucas.ac.cn (C.Z.)

Contents

Figure S1. The details pathway of 1-hexene insertion into **V** catalysts, $\mathbf{V_{H12}}$ (blue), $\mathbf{V_{H21}}$ (green), $\mathbf{V_{B21}}$ (black), and $\mathbf{V_{B12}}$ (yellow).

Figure S2. Optimized structures of 1-hexene insertion into **V** catalysts reaction pathway shown in Figure S1.

Table S1: Additional computational details.

Table S2: Energies and cartesian coordinates of all the optimized structures.

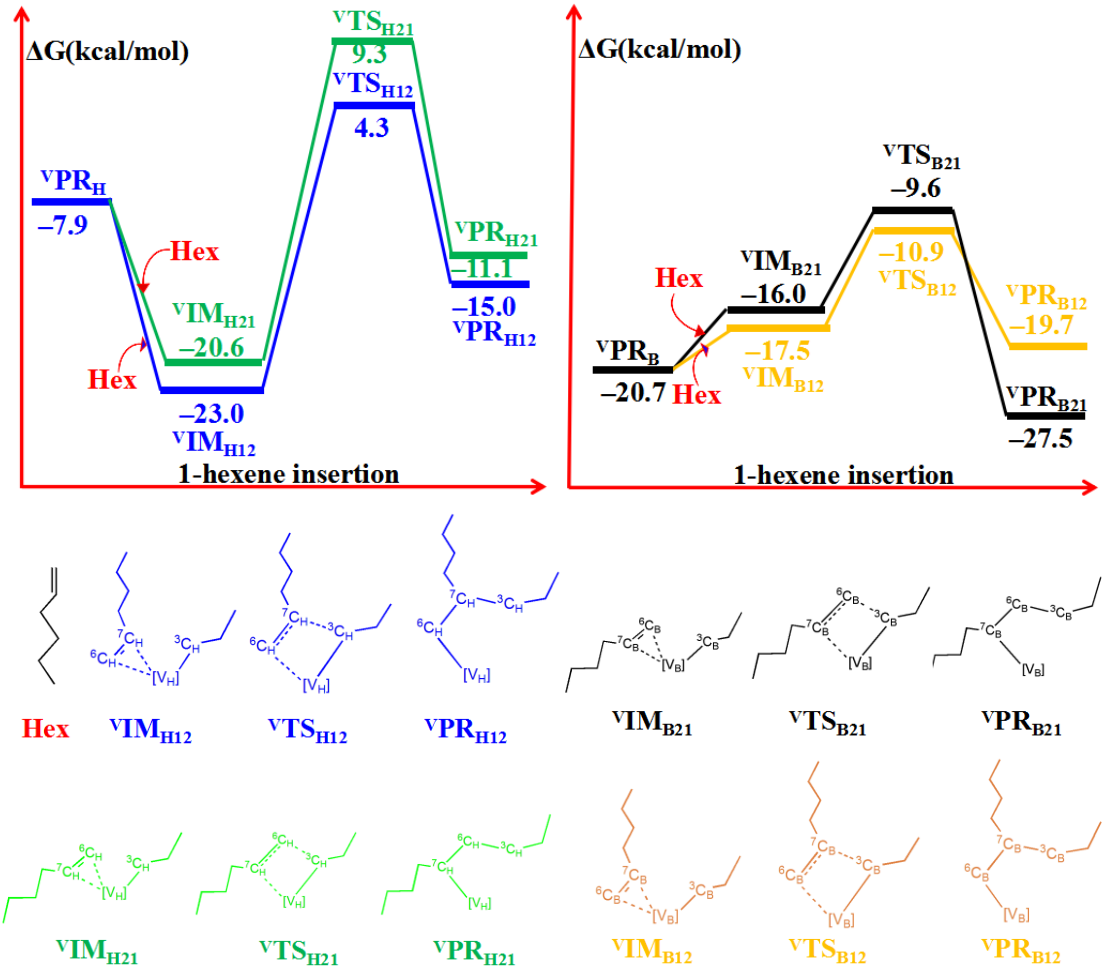

Figure S1. The reaction pathway of 1-hexene insertion into V catalysts, **V$_{H12}$** (**blue**), **V$_{H21}$** (**green**), **V$_{B21}$** (**black**), and **V$_{B12}$** (**yellow**); the relative free energy is given in kcal/mol. Depending on which carbon (^{6}C or ^{7}C) in 1-hexene forms a C–C bond with ^{3}C carbon of [V]–alkyl, there are two insertion modes. The 1,2-insertion forming V-^{6}C and ^{7}C–^{3}C bonds and the 2,1-insertion forming V-^{7}C and ^{6}C–^{3}C bonds.

For **V$_{H}$** catalysts, there are 1,2 insertion **V$_{H12}$** or 2,1 insertion **V$_{H21}$** pathway for 1-hexene insertion reactions. The free energies illustrate that **V$_{H12}$** pathway is comparatively preferred to **V$_{H21}$** pathway both thermodynamically and kinetically. Compared to the reactant (^{V}**PR$_{H}$** + **Hex**), the free energy barrier of ^{V}**TS$_{H12}$** is 12.2 kcal/mol, which is lower than 17.2 kcal/mol for ^{V}**TS$_{H21}$** catalyst; and the reaction for **V$_{H12}$** pathway is exothermic by 7.1 kcal/mol larger than 3.2 kcal/mol for **V$_{H21}$** pathway.

For **V$_{B}$** catalysts, there are also 1,2 insertion **V$_{B12}$** or 2,1 insertion **V$_{B21}$** pathway for 1-hexene insertion reactions. The free energy results illustrate that **V$_{B21}$** pathway is preferred to **V$_{B12}$** pathway thermodynamically. Compared to the reactant (^{V}**PR$_{B}$** + **Hex**), the free energy barrier of ^{V}**TS$_{B21}$** is 11.1 kcal/mol, which is slightly higher than 9.8 kcal/mol for ^{V}**TS$_{B12}$** catalyst; and the product ^{V}**PR$_{B21}$** is lower than the the reactant (^{V}**PR$_{B}$** + **Hex**) by 6.8 kcal/mol, however, the product

^{V}**PR**$_{B12}$ is higher than the the reactant (^{V}**PR**$_{B}$ **+ Hex**) by 1.0 kcal/mol. Because the energies of both transition states (^{V}**TS**$_{B21}$ and ^{V}**TS**$_{B12}$) are not high, they can easily be crossed in the reaction, so the product (^{V}**PR**$_{B21}$) with lower product energy ^{V}**PR**$_{B21}$ is the main reaction product.

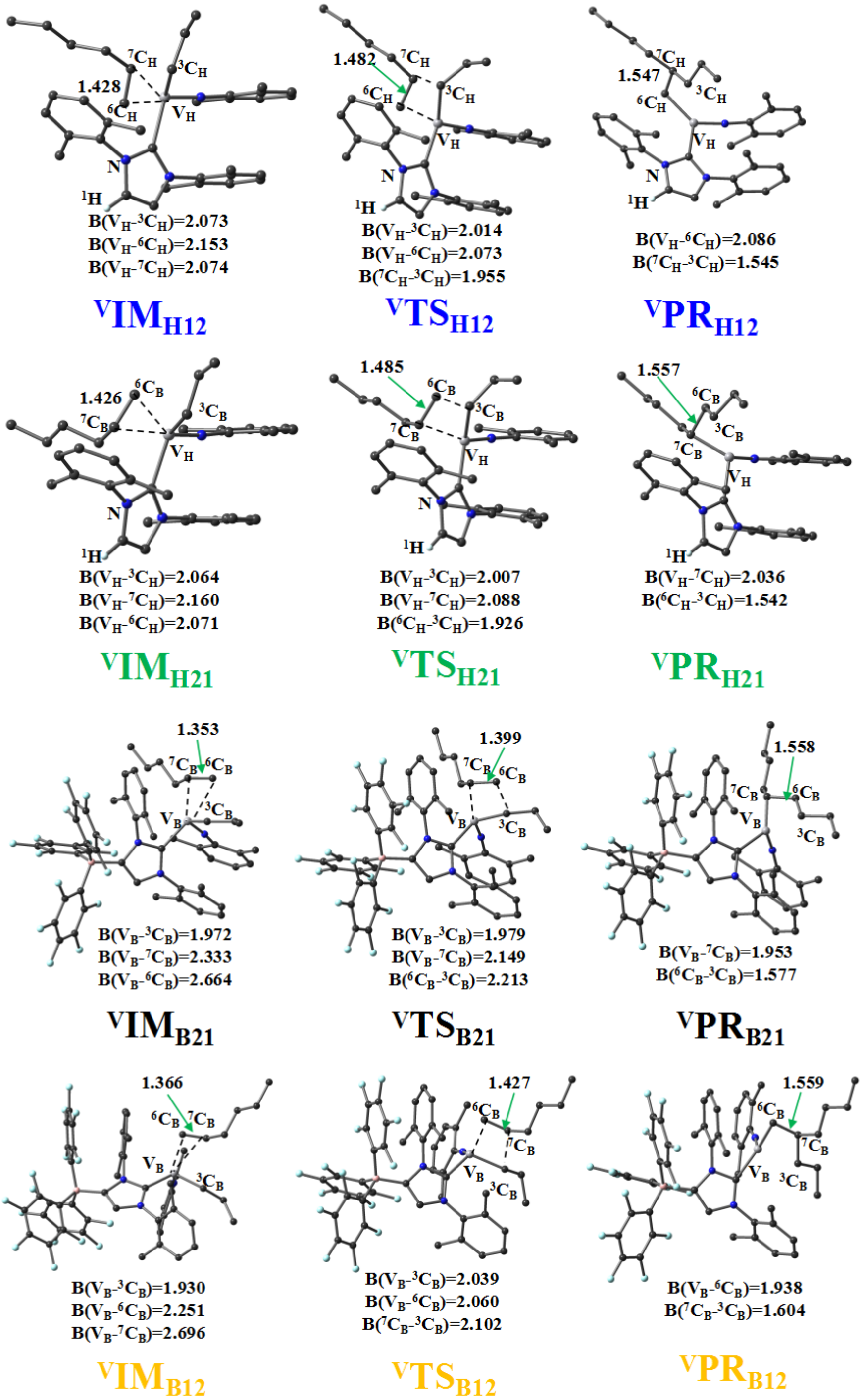

Figure S2. Optimized structures in 1-hexene insertion into Ti catalysts reaction pathway shown in Figure S1, the key length is in angstroms. For clarity, most hydrogen atoms are omitted.

Table S1: Additional computational details.

The global reactivity indexvalues were performed using the Multiwfn program [1,2]. The calculations also used a mixed basis set (SDD for Pd and 6-31G(d) for other non-metal atoms).

Vertical ionization potential: $\mathrm{VIP} = E_{N-1} - E_N$,

Vertical electron affinity: $\mathrm{VEA} = E_\mathrm{N} - E_{N-1}$,

where N denotes the number of electrons for a stable system.

Mulliken electronegativity: $\chi = \dfrac{\mathrm{VIP} + \mathrm{VEA}}{2}$,

Chemical potential: $\mu = -\chi$,

Chemical hardness: $\eta = \mathrm{VIP} - \mathrm{VEA}$ [3],

Electrophilicity index [4]: $\omega = \dfrac{\mu^2}{2\eta}$,

Nucleophilicity index: $N_{Nu} = E_{HOMO}(Nu) - E_{HOMO}(TCE)$, where Nu refers to nucleophile and TCE denotes tetracyanoethylene [5].

References

1. Lu, T.; Chen, F.-W. Multiwfn: A multifunctional wavefunction analyzer. *J. Comput. Chem.* **2012**, *33*, 580–592.
2. Lu, T.; Chen, Q.X. Realization of conceptual density functional theory and information-theoretic approach in Multiwfn program. In *Conceptual Density Functional Theory*; WILEY-VCH GmbH: Weinheim, Germany, 2022; pp. 631–647. https://doi.org/10.1002/9783527829941.ch31.
3. Parr, R.G. ; Pearson, R.G. Absolute hardness-companion parameter to absolute electronegativity. *J. Am. Chem. Soc.* **1983**, *105*, 7512–7516.
4. Parr, R.G.; Von Szentpaly, L.; Liu, S.B. Electrophilicity index. *J. Am. Chem. Soc.* **1999**, *121*, 1922–1924.
5. Domingo, L.R.; Chamorro, E.; Pérez, P. Understanding the reactivity of captodative ethylenes in polar cycloaddition reactions. a theoretical study. *J. Org. Chem.* **2008**, *73*, 4615–4624.

Table S2: Energies and Cartesian coordinates of all the structures for the main text.

C_2H_4	Ti_H	$^{Ti}IM_H$	$^{Ti}TS_H$	$^{Ti}PR_H$
-78.54301	-2085.10638	-2163.68025	-2163.60546	-2163.65671
	Ti_B	$^{Ti}IM_B$	$^{Ti}TS_B$	$^{Ti}PR_B$
	-4292.74348	-4371.28224	-4371.27096	-4371.30038
**Hex**	$^{Ti}IM_{H21}$	$^{Ti}TS_{H21}$	$^{Ti}PR_{H21}$	
-235.67701	-2399.35239	-2399.29187	-2399.35243	
	$^{Ti}IM_{H12}$	$^{Ti}TS_{H12}$	$^{Ti}PR_{H12}$	
	-2399.35488	-2399.28600	-2399.34569	
	$^{Ti}IM_{B21}$	$^{Ti}TS_{B21}$	$^{Ti}PR_{B21}$	
	-4606.97948	-4606.95760	-4607.00068	
	$^{Ti}IM_{B12}$	$^{Ti}TS_{B12}$	$^{Ti}PR_{B12}$	
	-4606.97670	-4606.96697	-4606.99302	
	V_H	$^{V}IM_H$	$^{V}TS_H$	$^{V}PR_H$
	-2193.90163	-2272.47733	-2272.42377	-2272.45720
	V_B	$^{V}IM_B$	$^{V}TS_B$	$^{V}PR_B$
	-4401.49162	-4480.05034	-4480.02994	-4480.06761
	$^{V}IM_{H12}$	$^{V}TS_{H12}$	$^{V}PR_{H12}$	
	-2508.15830	-2508.11488	-2508.14558	
	$^{V}IM_{H21}$	$^{V}TS_{H21}$	$^{V}PR_{H21}$	
	-2508.15447	-2508.10688	-2508.13938	
	$^{V}IM_{B21}$	$^{V}TS_{B21}$	$^{V}PR_{B21}$	
	-4715.73716	-4715.72701	-4715.75539	
	$^{V}IM_{B12}$	$^{V}TS_{B12}$	$^{V}PR_{B12}$	
	-4715.73946	-4715.72897	-4715.74304	

C_2H_4

C	0.00000000	0.00000000	0.66507000
H	0.00000000	0.92373600	1.23699100
H	0.00000000	-0.92373600	1.23699100
C	0.00000000	0.00000000	-0.66507000
H	0.00000000	-0.92373600	-1.23699100
H	0.00000000	0.92373600	-1.23699100

Ti_H

Ti	-0.03960800	1.13308900	-0.12287000

N	-2.07224200	-1.28336700	0.17682700
N	-0.09789100	-2.13373000	0.15418600
C	-0.74478500	-0.91603300	0.05181800
C	-2.23184700	-2.65596100	0.34393300
C	-0.98949700	-3.19322600	0.32070700
H	-0.65114200	-4.21195200	0.41755200
C	-3.15630500	-0.35593200	0.05667600
C	-3.53305900	0.07507700	-1.22517000
C	-4.57124400	1.00867000	-1.31782900
H	-4.87751600	1.36238200	-2.29799100
C	-5.21873100	1.47138500	-0.17339200
H	-6.02356000	2.19474800	-0.26297100
C	-4.84188000	1.00494700	1.08498600
H	-5.34689000	1.36929800	1.97460700
C	-3.79890400	0.08451800	1.22274300
C	-2.85394700	-0.47103800	-2.45510200
H	-3.30311400	-0.05783400	-3.36125700
H	-2.93713000	-1.56286800	-2.49701100
H	-1.78560600	-0.23243000	-2.44940100
C	-3.34317900	-0.39451300	2.57645400
H	-3.50078100	-1.47136800	2.70166400
H	-2.27415400	-0.20422000	2.70744900
H	-3.88446900	0.12249400	3.37177000
C	1.32080800	-2.28495800	0.25391100
C	2.01970300	-2.87754000	-0.80769800
C	3.41026500	-2.99369100	-0.69528400
H	3.97108700	-3.43328900	-1.51500200
C	4.07364800	-2.54777300	0.44415500
H	5.15328600	-2.63840900	0.51388400
C	3.35117300	-1.99362700	1.50175400
H	3.86665500	-1.66468100	2.39930100
C	1.96245300	-1.85843900	1.43016300
C	1.30237500	-3.38790900	-2.03249200
H	1.97769000	-3.41862400	-2.89123300
H	0.92271600	-4.40547300	-1.87745500
H	0.44257400	-2.76375600	-2.28655200
C	1.18795300	-1.26914500	2.57973000
H	1.79690600	-1.25912700	3.48704800
H	0.87674900	-0.24057600	2.36869100
H	0.27377200	-1.83645000	2.77853800
C	1.23508100	0.28979900	-2.06762500
H	1.13216500	-0.69693900	-2.49336000
C	2.05562800	0.63597000	-0.96631000
H	2.75664400	-0.02262400	-0.47674900

C	1.86053800	2.03828900	-0.66473800
C	0.87822400	2.51548100	-1.61859700
H	0.51953100	3.53234300	-1.70617600
C	0.50649300	1.42722700	-2.46429400
H	-0.23486800	1.46428200	-3.25295100
C	2.75437100	2.85896700	0.25229700
C	2.89957800	2.15068100	1.61421600
H	3.30075000	1.13895800	1.49627800
H	1.92762500	2.07788300	2.11384300
H	3.57889000	2.70571700	2.27145500
C	4.14820000	2.99093300	-0.39918000
H	4.82450200	3.57843700	0.23356600
H	4.59693000	2.00561600	-0.55879000
H	4.07220200	3.48547500	-1.37285200
C	2.17390300	4.26388400	0.47612900
H	2.82174100	4.83953500	1.14604300
H	2.09723000	4.81545500	-0.46629000
H	1.17839400	4.21495100	0.92605300
C	-0.96503000	2.10719500	1.54705400
H	-0.62744400	1.72076200	2.52228300
H	-0.66467300	3.16618900	1.50416800
H	-2.06247600	2.08821700	1.55240400
H	-3.20381400	-3.10981700	0.45140600

$^{Ti}IM_H$

N	2.21848400	0.86210700	-0.52269400
N	0.57824200	2.23912400	-0.46604000
C	0.89208400	0.93842600	-0.14609200
C	2.69288900	2.06563100	-1.04195700
C	1.66022900	2.93445000	-1.00551700
H	1.57610600	3.96720600	-1.30172800
C	3.11412600	-0.23462500	-0.27852500
C	3.38717200	-1.15763300	-1.29731900
C	4.25281300	-2.21955200	-1.00026600
H	4.46587000	-2.95524500	-1.77077600
C	4.84069500	-2.33413700	0.25492900
H	5.49987700	-3.16956500	0.47057800
C	4.61442200	-1.35746100	1.22495700
H	5.10948500	-1.42189000	2.18924300
C	3.76605600	-0.27871400	0.96758000
C	2.81606000	-1.03221000	-2.68816700
H	2.09363000	-1.82768000	-2.89287900
H	3.61626300	-1.11815000	-3.43016300
H	2.31373800	-0.07636400	-2.84058600

C	3.61519000	0.84271300	1.96334000
H	4.22308300	1.70401500	1.66033500
H	2.58178700	1.17900700	2.03873900
H	3.94956400	0.52868000	2.95445700
C	-0.68524000	2.89582000	-0.27862600
C	-1.59437900	2.92159300	-1.34380300
C	-2.79685300	3.60963000	-1.15686200
H	-3.52140700	3.63806200	-1.96531700
C	-3.06975100	4.25059800	0.05038500
H	-4.00867600	4.77996300	0.18103200
C	-2.14377900	4.21380700	1.09029400
H	-2.36160500	4.70912500	2.03177400
C	-0.93087000	3.53410600	0.94353500
C	-1.29267600	2.20078900	-2.63099400
H	-2.08643600	2.36268200	-3.36369700
H	-0.34637800	2.53346300	-3.07040100
H	-1.20235100	1.12643300	-2.45107500
C	0.06620800	3.45125700	2.06926000
H	-0.24589400	4.07249100	2.91161800
H	0.16108500	2.41830800	2.41968200
H	1.06338900	3.77409100	1.75262600
C	-0.38929800	-1.37114500	-1.66007500
H	0.09561300	-0.71765000	-2.36767400
C	-1.77882300	-1.42348400	-1.39554900
H	-2.52828700	-0.78467000	-1.84038000
C	-2.02804400	-2.43150800	-0.42717900
C	-0.77190100	-2.98814300	-0.08009500
H	-0.61073400	-3.78131300	0.63471300
C	0.24148500	-2.34553400	-0.83365300
H	1.30024400	-2.54788700	-0.76348800
C	-3.38003200	-2.94796500	0.03684200
C	-4.51295700	-1.98567400	-0.35721800
H	-4.57842400	-1.87608300	-1.44476500
H	-4.36761500	-0.99456000	0.07790000
H	-5.47395400	-2.37648400	-0.00641500
C	-3.62311900	-4.30512500	-0.66445200
H	-4.59962200	-4.71493100	-0.38129200
H	-3.60128300	-4.18953500	-1.75281300
H	-2.85388200	-5.03268700	-0.38798100
C	-3.40005600	-3.17021600	1.56047000
H	-4.35919600	-3.60362500	1.86504400
H	-2.60795700	-3.85664000	1.87330500
H	-3.25616100	-2.23226500	2.09987600
Ti	-0.53194200	-0.66835600	0.59644300

C	0.69600300	-0.60088200	2.40392000
C	-2.24706200	0.47501400	1.07859400
C	-0.39236400	-1.52479200	2.54901600
H	1.69996300	-0.99971200	2.29160000
H	0.66771700	0.35267000	2.92910600
H	-2.98599600	-0.19456400	1.53043000
H	-1.93438000	1.18895800	1.85288900
H	-2.73289500	1.02875400	0.27121800
H	-0.18516200	-2.59097200	2.59813500
H	-1.26510900	-1.22994400	3.13060300
H	3.71228000	2.17351800	-1.37472700

$^{Ti}TS_H$

N	2.14768100	1.20808900	-0.42073700
N	0.17182300	2.11996500	-0.42213500
C	0.83318000	0.95186200	-0.01910800
C	2.25132400	2.41895500	-1.11312400
C	1.02666400	2.98544700	-1.11231200
H	0.68614800	3.93600200	-1.48910500
C	3.25730100	0.34904900	-0.17155500
C	3.83267800	-0.35261100	-1.24653900
C	4.92828000	-1.18096400	-0.98459300
H	5.38031000	-1.73219200	-1.80432400
C	5.43360000	-1.31258200	0.30742300
H	6.28315300	-1.96200000	0.49518300
C	4.84320300	-0.61460000	1.35951300
H	5.23379100	-0.71792000	2.36784600
C	3.74916500	0.22771000	1.14013000
C	3.27085600	-0.23371800	-2.64060700
H	3.72562000	-0.97360900	-3.30359600
H	3.44725000	0.75754200	-3.07285700
H	2.18926700	-0.38836100	-2.63797700
C	3.09590300	0.95878100	2.28164000
H	2.90595500	2.00597900	2.02810400
H	2.12397100	0.51148800	2.51736000
H	3.72216500	0.92213400	3.17629500
C	-1.17592500	2.42851200	-0.07628600
C	-2.15446900	2.52944400	-1.08476700
C	-3.47947900	2.75436200	-0.69802900
H	-4.24770800	2.81211200	-1.46417900
C	-3.82382300	2.89007600	0.64583300
H	-4.86065700	3.04341200	0.92876800
C	-2.82969100	2.85602400	1.62287100
H	-3.08596400	3.01385300	2.66658900

C	-1.49018700	2.65540300	1.27824400
C	-1.79584600	2.41344600	-2.54428200
H	-2.67663900	2.16451900	-3.14140300
H	-1.39182400	3.35656400	-2.93209100
H	-1.03547000	1.64976600	-2.71227700
C	-0.39729100	2.78855200	2.30589500
H	-0.80844200	2.78732500	3.31792800
H	0.33699500	1.98786800	2.21449300
H	0.14090200	3.73360500	2.15980500
C	-0.49229800	-0.97030400	-2.02481300
H	-0.03644800	-0.24936600	-2.68692600
C	-1.75192700	-0.84299500	-1.36566100
H	-2.38714500	0.02824600	-1.38734300
C	-2.05312400	-2.05680700	-0.68138800
C	-0.95564800	-2.92266400	-0.87426400
H	-0.84798900	-3.91319800	-0.45445900
C	0.01236300	-2.25844200	-1.68270500
H	0.96919700	-2.66757000	-1.98052600
C	-3.37715900	-2.40159900	-0.01656000
C	-4.14251400	-1.13762700	0.41455800
H	-4.35553600	-0.48498000	-0.43672700
H	-3.58582800	-0.55320900	1.15137500
H	-5.10100900	-1.41881500	0.86380000
C	-4.22889600	-3.15741500	-1.06324700
H	-5.20737700	-3.42355900	-0.64680500
H	-4.38975700	-2.53779800	-1.95091300
H	-3.72817100	-4.07694000	-1.38110400
C	-3.17020300	-3.31058600	1.20729900
H	-4.13739700	-3.60033800	1.63208800
H	-2.63877700	-4.22916100	0.94082800
H	-2.59807100	-2.79935400	1.98462100
Ti	-0.03290400	-0.94931000	0.21756100
C	1.37632600	-2.29210300	1.15320300
C	-0.90219100	-0.55522400	2.28917800
C	0.41893500	-2.16216500	2.21193200
H	1.37003800	-3.22789800	0.60367000
H	2.36376900	-1.85639600	1.28340300
H	-1.50987800	-1.17636800	2.94958400
H	-0.45691900	0.25617300	2.85693700
H	-1.59645400	-0.10869700	1.55050000
H	-0.31312400	-2.95695300	2.32106200
H	0.75424600	-1.76914500	3.16898100
H	3.19651100	2.77436700	-1.49010800

$^{Ti}PR_H$

N	2.08451600	1.43312400	0.15632200
N	0.07395200	2.23028100	0.25607800
C	0.75036500	1.01172000	0.11650400
C	2.20072900	2.81880500	0.27059100
C	0.94943100	3.31919600	0.32402000
H	0.58862400	4.32963800	0.42564800
C	3.21100200	0.57488300	-0.01319800
C	3.58501400	0.19793300	-1.31287600
C	4.67384900	-0.66654300	-1.45499000
H	4.97552000	-0.97844100	-2.45070900
C	5.36649500	-1.13102500	-0.33752400
H	6.20561100	-1.80842100	-0.46468500
C	4.98645900	-0.73010200	0.94171900
H	5.52811500	-1.09410500	1.81006200
C	3.90119500	0.13318800	1.12469600
C	2.81826300	0.70526800	-2.50584100
H	3.26366000	0.34644800	-3.43683100
H	2.80133800	1.80042300	-2.52878900
H	1.77454300	0.37390100	-2.45885900
C	3.47455500	0.57888700	2.49998900
H	3.78067400	1.61302800	2.69806400
H	2.38678700	0.54824800	2.60367100
H	3.92003900	-0.05467500	3.27094000
C	-1.34017200	2.37143600	0.33911200
C	-2.03313000	2.94136000	-0.74282100
C	-3.42313700	3.06371000	-0.64545000
H	-3.97613900	3.48450100	-1.48034100
C	-4.10010300	2.63756200	0.49534600
H	-5.18037600	2.73002800	0.55216500
C	-3.38935400	2.09895000	1.56798300
H	-3.91458100	1.78390200	2.46490600
C	-1.99944700	1.96552500	1.51332200
C	-1.30242100	3.39393200	-1.98115700
H	-1.98201600	3.43957800	-2.83579100
H	-0.86744100	4.39220400	-1.85028700
H	-0.47840600	2.71891200	-2.22154500
C	-1.22555000	1.42605400	2.68736900
H	-1.88357900	1.26204700	3.54387500
H	-0.72775000	0.48110800	2.44639200
H	-0.43170900	2.11966400	2.98409700
C	-1.11306300	-0.30545200	-2.27196800
H	-0.87620700	0.60327500	-2.80386900
C	-2.08176000	-0.45646500	-1.23295400

H	-2.69320500	0.33630800	-0.82953800
C	-2.13966600	-1.83412000	-0.84115300
C	-1.19517200	-2.51809400	-1.64760800
H	-0.94495400	-3.56709700	-1.57630300
C	-0.56244600	-1.58736500	-2.50852800
H	0.24764500	-1.81589800	-3.18978700
C	-3.17658200	-2.45235800	0.08360700
C	-3.46800600	-1.52804000	1.27893100
H	-3.82666400	-0.54889200	0.95444400
H	-2.56969400	-1.37120400	1.88575300
H	-4.23359200	-1.97021000	1.92620300
C	-4.48066000	-2.64472800	-0.72294800
H	-5.26550800	-3.08914900	-0.09931400
H	-4.84483900	-1.68544200	-1.10339400
H	-4.30966800	-3.30283600	-1.58055200
C	-2.70826500	-3.81760600	0.61133800
H	-3.47237700	-4.25437700	1.26330500
H	-2.52752900	-4.52316400	-0.20501900
H	-1.78310300	-3.72028400	1.18557000
Ti	-0.03077700	-0.91193000	-0.27829400
C	1.47470900	-2.41603500	-0.07769400
C	0.56413300	-2.18675900	2.28957000
C	1.13252100	-3.14615000	1.23251800
H	1.64341600	-3.12617100	-0.89320600
H	2.40708600	-1.85062500	0.05238400
H	0.25006000	-2.70068100	3.20452400
H	1.28570400	-1.41293700	2.56398300
H	-0.35421100	-1.69202000	1.91491200
H	0.38658000	-3.92585500	1.03808300
H	2.00332800	-3.65932000	1.67033600
H	3.16393000	3.30183500	0.30574100

Ti$_B$

Ti	3.34482500	-0.07687700	-0.24762200
F	-4.06710300	-1.47969100	1.44437800
F	-4.29952100	-1.34964600	-1.68109400
F	-3.01567400	0.22313400	5.69716900
F	-1.83833600	-0.89292100	-2.78631700
F	-1.64848000	-2.87888400	1.53722600
F	-2.70310300	2.57929700	0.50515600
F	-4.62172800	-1.26964000	4.06259100
F	-0.27366500	1.33647400	2.05872900
F	-4.88557000	3.87426300	-0.28189600
F	-6.45562500	-0.02515500	-2.44145200

F	-0.95625100	-5.17144500	0.43925700
F	-6.80618100	2.60125100	-1.77075600
F	-1.19612100	-3.25491000	-3.87313900
F	-0.83316200	1.50675000	4.63361700
F	-0.71623500	-5.42203600	-2.27858800
N	0.64831000	0.03403900	-0.53522000
N	0.95029500	2.12718100	-0.94601600
C	1.62537700	0.97101100	-0.76898400
C	-0.63807500	0.57479100	-0.50700700
C	-0.40610400	1.90059100	-0.82830200
H	-1.10629000	2.70710700	-0.94491600
C	1.20941100	-1.29856900	-0.51400900
C	1.59454700	-1.89550700	0.72042000
C	2.46649400	-2.99598500	0.64945500
H	2.79456300	-3.45600900	1.57548700
C	2.87734900	-3.52922700	-0.57607500
H	3.54372800	-4.38471700	-0.59551600
C	2.42298400	-2.97126600	-1.76704700
H	2.72950100	-3.39539300	-2.71787700
C	1.59287400	-1.83700500	-1.77027500
C	1.06669600	-1.44182300	2.04962500
H	1.71741900	-1.77849700	2.85868900
H	0.08065600	-1.88479200	2.20400900
H	0.94327900	-0.36530200	2.10356000
C	1.17952700	-1.22743100	-3.08247700
H	0.43792500	-0.44204100	-2.96526700
H	2.04354700	-0.81723100	-3.61362600
H	0.74601600	-1.99873700	-3.72253800
C	1.55829600	3.40303400	-1.22915400
C	1.49708000	4.40356200	-0.24404600
C	2.13429900	5.61653800	-0.52822300
H	2.11191100	6.40783800	0.21417400
C	2.78482700	5.81863400	-1.74408300
H	3.27003500	6.76826300	-1.94482700
C	2.79686500	4.81580500	-2.71148600
H	3.27660400	4.99031700	-3.66938600
C	2.17533100	3.58460000	-2.47695000
C	0.78298700	4.19650400	1.06833100
H	1.01031100	5.01226500	1.75668700
H	-0.30391500	4.16217600	0.94098700
H	1.06227000	3.25596200	1.54717800
C	2.14370800	2.52064000	-3.54631800
H	2.40544100	2.95018300	-4.51510400
H	2.84992100	1.71117200	-3.34055900

H	1.15035000	2.07063900	-3.63390800
C	-2.15207400	-0.09961000	1.61090900
C	-1.39124700	0.66270600	2.48869700
C	-1.64496700	0.77792500	3.85249500
C	-2.74735700	0.12832000	4.39415600
C	-3.56173000	-0.63413200	3.55754800
C	-3.25423600	-0.72396200	2.20480400
C	-1.79444800	-1.74583300	-0.57299800
C	-1.57844700	-2.89560400	0.18543000
C	-1.21577200	-4.12912900	-0.35906900
C	-1.09381700	-4.26245200	-1.73594100
C	-1.34981300	-3.15639500	-2.54260400
C	-1.67986300	-1.94506500	-1.95167800
C	-3.33448800	0.55560300	-0.60472400
C	-4.36268400	-0.05068800	-1.33252500
C	-5.51998400	0.61532400	-1.73442000
C	-5.70655500	1.94970900	-1.39072100
C	-4.72883600	2.59379800	-0.63970000
C	-3.59455000	1.88528400	-0.26048700
C	3.95639300	1.92623400	0.72671600
H	3.59077000	2.85073000	0.30273500
C	5.17395600	1.27463800	0.39264700
H	5.89766900	1.62948500	-0.32405200
C	5.28418500	0.08621000	1.15718100
C	4.10440300	0.00844300	1.96248600
H	3.86632400	-0.78745400	2.65396400
C	3.30780700	1.15000100	1.72461100
H	2.34557900	1.36324400	2.16912700
C	6.45324900	-0.88354300	1.20302700
C	7.56123700	-0.46391200	0.22427300
H	7.94262000	0.53419600	0.45866100
H	7.21995400	-0.46542200	-0.81482800
H	8.39895600	-1.16223000	0.29652900
C	7.01691700	-0.85446000	2.64305600
H	7.88091500	-1.52149300	2.71585100
H	7.34092900	0.15320800	2.91797700
H	6.27332100	-1.18504700	3.37402800
C	5.98463500	-2.31495300	0.87374700
H	6.82728300	-3.00899600	0.93799200
H	5.21971100	-2.65957300	1.57572800
H	5.57993000	-2.38357000	-0.14192100
C	4.48618700	-0.10617500	-1.97175200
H	4.80619400	0.88082900	-2.31341100
H	5.37254200	-0.73765200	-1.85155200

H	3.83182700	-0.54877600	-2.72910300
B	-1.99577000	-0.20731100	-0.02478000

$^{Ti}IM_B$

F	4.48770800	0.43844800	1.45258800
F	4.70946400	0.26132200	-1.65040800
F	3.09377100	-1.37333100	5.55994700
F	2.20907600	0.69770200	-2.77452000
F	2.57153200	2.40956200	1.64760000
F	1.82921500	-3.04189100	0.23913000
F	4.99381000	-0.14405400	4.02469700
F	0.13368700	-1.43805600	1.92287200
F	3.45320700	-4.93710700	-0.66050200
F	6.30008900	-1.65565900	-2.52052900
F	2.86421800	4.84277800	0.66662000
F	5.72319600	-4.28757300	-2.05810900
F	2.50342700	3.17614300	-3.73793200
F	0.66277100	-2.00224800	4.44846600
F	2.86144900	5.28685600	-2.03111300
N	-0.51194500	0.45449800	-0.28177500
N	-1.37819600	-1.35280000	-1.08461300
C	-1.70746500	-0.12173000	-0.60901600
C	0.57721300	-0.39589700	-0.50775100
C	-0.01242000	-1.51239700	-1.06600500
H	0.43508500	-2.42620600	-1.41268300
C	-0.51761300	1.88222700	-0.03048700
C	-0.47074200	2.40613000	1.27994000
C	-0.42531500	3.80282700	1.41788400
H	-0.37043000	4.22460700	2.41620200
C	-0.37327100	4.63693200	0.30651000
H	-0.28326900	5.70997300	0.43810700
C	-0.41870200	4.09519400	-0.98116500
H	-0.36700500	4.74941700	-1.84533800
C	-0.51068200	2.71794400	-1.17807400
C	-0.35040300	1.56010500	2.51524300
H	-0.87862400	2.02390300	3.35284200
H	0.70455100	1.49639600	2.79016100
H	-0.70796200	0.54526300	2.37163500
C	-0.60053200	2.16615000	-2.57867600
H	-0.09965500	1.20453900	-2.68405100
H	-1.64635600	2.03495400	-2.88260600
H	-0.13822400	2.85558900	-3.28701100
C	-2.30567300	-2.35057900	-1.55877500
C	-2.60858700	-3.44776900	-0.73721700

C	-3.57602900	-4.34962500	-1.20467100
H	-3.83556400	-5.20476300	-0.58776000
C	-4.18703800	-4.17245000	-2.44195600
H	-4.93536000	-4.88084200	-2.78236300
C	-3.81010300	-3.11063500	-3.26492600
H	-4.24920800	-3.00536000	-4.25195500
C	-2.84519000	-2.19002600	-2.84851700
C	-1.95101500	-3.69664400	0.59965500
H	-2.66589300	-3.57323500	1.42112900
H	-1.59731600	-4.73037600	0.64776700
H	-1.09583900	-3.05143000	0.79261200
C	-2.34999500	-1.11948300	-3.78711800
H	-3.04310700	-0.98163400	-4.61924400
H	-2.21094900	-0.16007500	-3.28947500
H	-1.37840900	-1.40363100	-4.20755200
C	2.28734600	-0.42684500	1.56015000
C	1.38005400	-1.08726100	2.38076300
C	1.61414000	-1.40254900	3.71566000
C	2.84226000	-1.08314600	4.28207200
C	3.80578600	-0.45081700	3.49721000
C	3.51573300	-0.15289200	2.17013400
C	2.45572000	1.39928400	-0.52052400
C	2.62718600	2.51189400	0.29949500
C	2.78535800	3.80986600	-0.18372800
C	2.77617100	4.04168900	-1.55269600
C	2.60190900	2.96467200	-2.41669100
C	2.44358000	1.69249800	-1.88656100
C	3.12640000	-1.27299600	-0.71924000
C	4.31649500	-1.00165200	-1.40240500
C	5.18704000	-1.99046400	-1.86068600
C	4.90237400	-3.33023700	-1.62290300
C	3.74857800	-3.65625700	-0.91839200
C	2.91452400	-2.63462700	-0.47855200
C	-3.21347100	-0.64151400	1.75632600
H	-2.26362000	-1.13747600	1.88900400
C	-4.29536000	-1.08190200	0.93678100
H	-4.29140500	-1.96765400	0.32023100
C	-5.38435300	-0.18093300	1.06648200
C	-4.93347900	0.86320300	1.92058300
H	-5.53742500	1.68476200	2.27287400
C	-3.60592500	0.57835600	2.34801600
H	-3.00325600	1.19073500	3.00400700
C	-6.82424200	-0.43104900	0.64868300
C	-6.93003500	-1.44944000	-0.49921300

H	-6.45126000	-2.39899300	-0.24332900
H	-6.48560200	-1.08242600	-1.42516200
H	-7.98412600	-1.66082900	-0.69803100
C	-7.50295000	-1.03786200	1.90545400
H	-8.54950900	-1.26706100	1.68406700
H	-7.00789900	-1.96420300	2.21070200
H	-7.47758300	-0.34230100	2.74941900
C	-7.56151500	0.86765400	0.27775400
H	-8.61488700	0.64949000	0.08381000
H	-7.53041800	1.60083700	1.08990800
H	-7.15586900	1.33048300	-0.62603100
B	2.12707700	-0.14022000	-0.05370100
Ti	-3.41296500	0.90372600	0.04955200
C	-3.57028300	3.37030400	-0.27159400
C	-4.46845800	0.64020100	-1.68389400
C	-4.71875300	3.34225000	0.43659200
H	-2.64793700	3.71174800	0.18668500
H	-3.57321000	3.23636700	-1.35040700
H	-5.47609600	1.05909100	-1.68468500
H	-3.83810500	1.19533300	-2.39412800
H	-4.50179300	-0.40433800	-1.99460100
H	-4.72754500	3.62596100	1.48385800
H	-5.67728500	3.10637000	-0.01450400

^{Ti}TS$_B$

F	4.54168500	0.34887100	1.39864400
F	4.69110100	0.23772300	-1.72291000
F	3.18026600	-1.51652600	5.49373500
F	2.18002000	0.75915900	-2.77398200
F	2.65459900	2.35428200	1.68148300
F	1.78533500	-3.04820400	0.15897800
F	5.08090000	-0.29864900	3.94964900
F	0.15747500	-1.44312900	1.90801400
F	3.33499600	-4.95558000	-0.84129600
F	6.21165100	-1.69305000	-2.68971600
F	2.88597100	4.81633200	0.76066400
F	5.58148700	-4.32188000	-2.28400400
F	2.42196500	3.26565900	-3.67658900
F	0.71576000	-2.06685900	4.41371600
F	2.79726400	5.33411600	-1.92292700
N	-0.48281300	0.50388700	-0.23700900
N	-1.39258600	-1.25499100	-1.09714900
C	-1.69316700	-0.03678400	-0.56654800
C	0.58421900	-0.35719700	-0.50710800

C	-0.03178600	-1.44397200	-1.09625900
H	0.39836200	-2.34779600	-1.48723500
C	-0.46882600	1.92564100	0.05075500
C	-0.40578600	2.42005500	1.36950200
C	-0.39848300	3.81297300	1.53938000
H	-0.33326000	4.21232700	2.54628600
C	-0.42348600	4.67553800	0.45057200
H	-0.37833600	5.74817100	0.60606600
C	-0.48842300	4.16194700	-0.84775900
H	-0.49154600	4.83655800	-1.69794600
C	-0.52456800	2.78555900	-1.07463200
C	-0.26010500	1.55228500	2.58712900
H	-0.81267700	1.97578400	3.43036300
H	0.79410100	1.51693100	2.86992900
H	-0.58236600	0.53095500	2.41774900
C	-0.62481700	2.26815200	-2.48915200
H	-0.13400900	1.30456700	-2.62152100
H	-1.67267000	2.15939300	-2.79507500
H	-0.15586600	2.96876600	-3.18219500
C	-2.35326500	-2.22732900	-1.55153700
C	-2.55971500	-3.37217000	-0.75795100
C	-3.54896300	-4.27039900	-1.17126400
H	-3.73554600	-5.15743600	-0.57412500
C	-4.28138500	-4.04661100	-2.33763300
H	-5.04725800	-4.75409200	-2.63856900
C	-4.01156400	-2.93634500	-3.13205500
H	-4.54974200	-2.79066200	-4.06379900
C	-3.02378000	-2.01195700	-2.76616400
C	-1.73957900	-3.64699600	0.47782600
H	-2.20102600	-4.43559700	1.07499400
H	-0.72754200	-3.97457300	0.21800600
H	-1.61250300	-2.76425100	1.10499600
C	-2.64749000	-0.90315900	-3.71718900
H	-3.52982300	-0.49792300	-4.22012400
H	-2.11381200	-0.09028100	-3.22707100
H	-1.98628200	-1.29556800	-4.49800000
C	2.32616400	-0.47171700	1.52912600
C	1.41845400	-1.12870500	2.35162300
C	1.66818300	-1.47412100	3.67606200
C	2.91312800	-1.19452000	4.22653800
C	3.87707500	-0.56805000	3.43741400
C	3.57064600	-0.23694400	2.12194400
C	2.48608700	1.40122500	-0.50920400
C	2.66958300	2.49308300	0.33592200

C	2.79343400	3.80654200	-0.11517400
C	2.74237300	4.07533300	-1.47624100
C	2.56234300	3.01927400	-2.36439800
C	2.43571600	1.73095600	-1.86569200
C	3.10047400	-1.28412800	-0.78834100
C	4.27699200	-1.02222600	-1.49760700
C	5.10961100	-2.01866600	-2.00725400
C	4.79781700	-3.35737200	-1.79864400
C	3.65609300	-3.67525800	-1.07097800
C	2.86095100	-2.64646900	-0.57917800
C	-3.16945800	-0.43431300	1.98497900
H	-2.18861600	-0.83486400	2.19525700
C	-4.15582000	-1.03632200	1.16764000
H	-4.04773300	-1.97588600	0.64772500
C	-5.32304700	-0.21059200	1.15075000
C	-5.00978400	0.93548700	1.92399800
H	-5.66875400	1.77397000	2.10093600
C	-3.69006200	0.80747000	2.43298900
H	-3.17107300	1.52787600	3.05001100
C	-6.69727300	-0.62839400	0.65242800
C	-6.61622100	-1.60485000	-0.53463700
H	-6.01950600	-2.48964400	-0.29882600
H	-6.19414100	-1.14753400	-1.43224700
H	-7.62179500	-1.95010700	-0.78927300
C	-7.35013900	-1.36991500	1.84877900
H	-8.35391800	-1.70628900	1.57251400
H	-6.76357200	-2.24712300	2.13660400
H	-7.43677800	-0.71563000	2.72103500
C	-7.57297100	0.58191800	0.28960500
H	-8.57092700	0.24244500	0.00055600
H	-7.69673800	1.26218400	1.13736200
H	-7.16448600	1.14824800	-0.55134900
B	2.14468500	-0.14453600	-0.07388200
Ti	-3.42362900	0.92539500	0.12789800
C	-3.65037300	3.14776400	0.37443100
C	-4.52652400	1.02820400	-1.61818000
C	-4.61209600	3.01516300	-0.62394500
H	-3.96379800	3.30224300	1.39844800
H	-2.67371000	3.53861200	0.10534600
H	-5.55572800	1.09712100	-1.95486200
H	-3.82900000	1.29662600	-2.41439600
H	-4.34666500	-0.05619600	-1.36316700
H	-5.66206800	2.93098400	-0.36698200
H	-4.40158000	3.35453500	-1.63179700

$^{Ti}PR_B$

F	4.55215600	0.23829200	1.40463100
F	4.70399400	0.18220500	-1.73176100
F	3.11445000	-1.64743000	5.46432400
F	2.21694300	0.79583000	-2.76578300
F	2.72039100	2.29595600	1.71857200
F	1.71014700	-3.04775800	0.10868800
F	5.05950900	-0.46720400	3.94663400
F	0.11178400	-1.42344800	1.86807700
F	3.20075500	-4.98252900	-0.92591100
F	6.16545800	-1.77530000	-2.73154700
F	3.01092200	4.77023100	0.84476400
F	5.46175400	-4.39084600	-2.36557700
F	2.51636600	3.31388200	-3.62157600
F	0.63689500	-2.10047200	4.36533400
F	2.93338500	5.34012700	-1.82849300
N	-0.46139500	0.56549100	-0.23058000
N	-1.41611800	-1.17742000	-1.08466500
C	-1.69285500	0.04397800	-0.53623200
C	0.57972700	-0.31251700	-0.51808200
C	-0.06395400	-1.38566400	-1.11270900
H	0.34856500	-2.28544800	-1.53117200
C	-0.39439100	1.98377800	0.07303900
C	-0.34344100	2.45706800	1.39607900
C	-0.33489200	3.84675200	1.58642100
H	-0.28104000	4.23130500	2.59983600
C	-0.35917300	4.72513300	0.51053300
H	-0.32250500	5.79573600	0.68167900
C	-0.41201800	4.22891700	-0.79374200
H	-0.41450600	4.91410300	-1.63563400
C	-0.44161500	2.85502000	-1.03870900
C	-0.23655700	1.56910200	2.60381800
H	-0.80173900	1.98920500	3.44037300
H	0.80987100	1.50697900	2.91006100
H	-0.57597600	0.55743700	2.41062100
C	-0.54455000	2.35943100	-2.46088000
H	-0.09206000	1.37875800	-2.60343000
H	-1.59208300	2.30034500	-2.78321000
H	-0.03948700	3.04871100	-3.14001800
C	-2.39258300	-2.12675600	-1.55305400
C	-2.54658600	-3.32625100	-0.82858400
C	-3.51997100	-4.22532600	-1.27411100
H	-3.66511500	-5.15316700	-0.72992100

C	-4.28881400	-3.95292200	-2.40652500
H	-5.03729500	-4.66628400	-2.73591500
C	-4.08054200	-2.78239300	-3.12746600
H	-4.65044600	-2.59264100	-4.03218100
C	-3.11395400	-1.84984800	-2.72590700
C	-1.69704900	-3.65542800	0.37412600
H	-2.13782100	-4.48201300	0.93449800
H	-0.68601400	-3.95571400	0.08091800
H	-1.57139200	-2.80656500	1.04710500
C	-2.81674800	-0.66824000	-3.61703100
H	-3.73765100	-0.16999400	-3.93592300
H	-2.16301200	0.06412100	-3.14634800
H	-2.31528000	-1.01078100	-4.52855800
C	2.31106700	-0.51239800	1.51355800
C	1.38029500	-1.15318000	2.32231900
C	1.61224600	-1.52724300	3.64188900
C	2.86301700	-1.29674200	4.20159500
C	3.84983700	-0.68926600	3.42581500
C	3.56000100	-0.32719900	2.11469400
C	2.53517600	1.39040300	-0.49010800
C	2.74105000	2.46112200	0.37646300
C	2.89253200	3.78002300	-0.04940000
C	2.84875800	4.07501400	-1.40537400
C	2.64946300	3.03992000	-2.31380500
C	2.49507100	1.74554200	-1.83983900
C	3.07064600	-1.30658000	-0.81724600
C	4.25326500	-1.06766400	-1.52505600
C	5.05588400	-2.07981900	-2.05185900
C	4.70683000	-3.41229200	-1.86332700
C	3.55827000	-3.70865100	-1.13751500
C	2.79463800	-2.66485000	-0.62797600
C	-3.18109800	-0.46550600	2.05289900
H	-2.19569200	-0.84898900	2.27686300
C	-4.14028300	-1.08044900	1.21377400
H	-4.00081700	-2.00623100	0.67809700
C	-5.31482600	-0.27090300	1.17380800
C	-5.04846000	0.86751600	1.98563600
H	-5.72100600	1.69617700	2.15608300
C	-3.74243800	0.75633000	2.52118600
H	-3.25373200	1.47944500	3.15871200
C	-6.65286700	-0.68474500	0.58030100
C	-6.47066800	-1.60241900	-0.64034600
H	-5.90530400	-2.50477900	-0.39785200
H	-5.95290900	-1.10597400	-1.46723000

H	-7.44885400	-1.91861600	-1.01249900
C	-7.37333700	-1.48058700	1.69868000
H	-8.34592800	-1.82807300	1.33780000
H	-6.78822400	-2.35404100	1.99989700
H	-7.53865300	-0.85834100	2.58282100
C	-7.51904600	0.52654300	0.19778200
H	-8.50496500	0.18514000	-0.12791300
H	-7.67408100	1.20379600	1.04247400
H	-7.08598000	1.09662100	-0.62905600
B	2.14812000	-0.15057900	-0.08457600
Ti	-3.43250900	0.91152100	0.23378200
C	-3.60487500	2.90895000	0.45342600
C	-4.59014800	1.63621700	-1.65306800
C	-4.68760500	2.83820300	-0.64983200
H	-3.93547700	3.42835300	1.34716300
H	-2.67090200	3.33600900	0.07640700
H	-5.37895900	1.72955900	-2.40108900
H	-3.63336100	1.61206900	-2.18683300
H	-4.81057800	0.61119100	-1.25554300
H	-5.68921500	2.85701900	-0.22035700
H	-4.57193600	3.71992800	-1.28806600

Hex

C	3.07456300	-0.19041200	-0.44197400
H	3.07343000	0.41970700	-1.34226400
H	3.96018200	-0.79228900	-0.26220400
C	2.03997000	-0.19242200	0.39750700
H	2.08089600	-0.82250000	1.28732700
C	0.77502900	0.59648200	0.21509000
H	0.63961900	1.27436100	1.07094200
H	0.86016700	1.23102400	-0.67578600
C	-0.47105600	-0.29643500	0.09734600
H	-0.53661600	-0.94728600	0.98033000
H	-0.35152800	-0.96495900	-0.76512800
C	-1.77076400	0.50027600	-0.04344300
H	-1.70128600	1.15159400	-0.92489600
H	-1.87894400	1.16967300	0.82059600
C	-3.00837700	-0.39133600	-0.16062600
H	-3.92281000	0.20175600	-0.26136000
H	-2.93930900	-1.04892100	-1.03432800
H	-3.11999400	-1.02907400	0.72337600

^{Ti}IM$_{H21}$

N	-0.92503900	-2.36188500	0.29081200

N	1.18434300	-2.36626700	-0.05876300
C	0.10326700	-1.51201400	-0.01481500
C	-0.50625200	-3.68202900	0.43305900
C	0.82648800	-3.68407900	0.21006700
H	1.55341700	-4.47978400	0.20556000
C	-2.32199800	-2.03061000	0.35595900
C	-3.00370500	-1.79265700	-0.84626400
C	-4.37176300	-1.51922100	-0.77305500
H	-4.91964500	-1.31708000	-1.68817000
C	-5.02947900	-1.49043200	0.45526200
H	-6.09090500	-1.26601300	0.49451100
C	-4.32866300	-1.74467200	1.63149500
H	-4.84269700	-1.72125800	2.58763500
C	-2.95879100	-2.02940800	1.60472400
C	-2.28301800	-1.81238600	-2.16864100
H	-2.99389300	-1.74613900	-2.99529000
H	-1.69814700	-2.73010300	-2.29155300
H	-1.58957600	-0.96878900	-2.24297200
C	-2.20380900	-2.32317400	2.87646700
H	-1.95381100	-3.38739000	2.96031900
H	-1.26566400	-1.76486300	2.92294300
H	-2.80570800	-2.05539100	3.74778400
C	2.50817200	-2.06409400	-0.53909000
C	2.73196900	-2.20257900	-1.92182500
C	3.99827500	-1.88936400	-2.41913500
H	4.18060700	-1.96399500	-3.48681400
C	5.02234000	-1.49323200	-1.56014900
H	6.00120300	-1.24714000	-1.95989600
C	4.79687600	-1.43868600	-0.18888100
H	5.60358800	-1.16534500	0.48478800
C	3.53820700	-1.73405400	0.35304500
C	1.65505800	-2.71921700	-2.84343100
H	1.90268800	-2.50383000	-3.88530400
H	1.54749100	-3.80588200	-2.74345100
H	0.67901900	-2.28373800	-2.62226400
C	3.35420400	-1.69352400	1.84707800
H	3.16771100	-0.67308400	2.19248800
H	2.51228000	-2.30480600	2.17439200
H	4.25910200	-2.05397400	2.34454200
C	1.15240200	0.70635900	-2.07723000
H	1.07010500	-0.22790700	-2.60587500
C	2.20851500	1.08689000	-1.21089700
H	3.07270800	0.49179400	-0.96936200
C	1.93737600	2.38432900	-0.71229600

C	0.69690100	2.79553700	-1.27373500
H	0.20854600	3.74004600	-1.09406400
C	0.21736200	1.76481800	-2.12299800
H	-0.70278800	1.77777500	-2.68597900
C	2.86751200	3.23530400	0.13420900
C	3.86498700	2.35232200	0.90356900
H	4.50484500	1.78430100	0.22115100
H	3.34165500	1.64156600	1.54736800
H	4.51349800	2.97177400	1.53201100
C	3.65657200	4.16047600	-0.81956900
H	4.34993400	4.79609600	-0.25616400
H	4.23574300	3.57309600	-1.53938700
H	2.97772300	4.80862100	-1.38214900
C	2.07101500	4.10140800	1.12643700
H	2.75288000	4.71553800	1.72494700
H	1.38852500	4.77745600	0.60264600
H	1.47633000	3.48535100	1.80299500
Ti	0.20153300	0.88558800	0.12854600
C	-1.91805200	1.23489800	0.10290100
C	0.54568500	0.30313700	2.18737400
C	-1.23991900	2.25466000	0.89152500
H	-2.35964200	0.43275200	0.69383300
H	-0.52393500	0.08638200	2.36747100
H	1.04097800	-0.66879800	2.28222800
H	-1.25829700	3.27518600	0.51526500
H	-1.19741400	-4.47515300	0.66585500
C	1.03624400	1.21802000	3.31207300
H	2.10307600	1.44400300	3.17904200
H	0.52182800	2.18532500	3.25783600
C	0.83805500	0.63184900	4.71776100
H	-0.22450800	0.44215500	4.91061600
H	1.36419300	-0.32542600	4.81846500
H	1.20798100	1.30307400	5.50305000
H	-1.30624700	2.21335100	1.97815300
C	-2.83666800	1.64992900	-1.03080900
H	-2.40351500	2.50447200	-1.56625400
H	-2.94223800	0.84534100	-1.77120700
C	-4.23930000	2.04703900	-0.54429900
H	-4.15697100	2.93661300	0.09391000
H	-4.63250100	1.24715000	0.09544500
C	-5.22816400	2.31116200	-1.68230100
H	-4.83959500	3.11040500	-2.32849300
H	-5.29090300	1.41490400	-2.31615900
C	-6.62785200	2.68462500	-1.18819500

H	-7.31986000	2.86331100	-2.01822200
H	-7.04959200	1.88543400	-0.56726000
H	-6.59876700	3.59277300	-0.57566500

$^{Ti}TS_{H21}$

N	-0.07866100	-2.61497800	0.27702600
N	1.96912900	-1.99929400	-0.03658900
C	0.67555700	-1.46128100	0.00303700
C	0.71577300	-3.76291200	0.38556100
C	1.98907200	-3.37774600	0.18041500
H	2.91218900	-3.93459100	0.17353200
C	-1.49652900	-2.75564100	0.15658300
C	-2.07942600	-2.71774800	-1.12446700
C	-3.46067700	-2.90840100	-1.22661000
H	-3.92550600	-2.87149900	-2.20771300
C	-4.23505100	-3.16150800	-0.09572300
H	-5.30628800	-3.30914600	-0.19282800
C	-3.63100200	-3.24423300	1.15702600
H	-4.23076100	-3.45792100	2.03705600
C	-2.25359100	-3.05168100	1.30494400
C	-1.23737100	-2.48845300	-2.35019500
H	-1.82961300	-2.62118100	-3.25887200
H	-0.39232700	-3.18475400	-2.38013500
H	-0.80762200	-1.47982600	-2.34105900
C	-1.60514500	-3.13994100	2.66256500
H	-0.88055400	-3.95914300	2.71699300
H	-1.05419200	-2.22347200	2.89654500
H	-2.35640900	-3.30136600	3.43929500
C	3.18063300	-1.29561300	-0.31684200
C	3.69142600	-1.33206200	-1.62361300
C	4.86707800	-0.62236200	-1.89294800
H	5.26503400	-0.62099500	-2.90359900
C	5.52122400	0.07958800	-0.88407500
H	6.42678400	0.63495400	-1.10888900
C	5.02517900	0.05547500	0.41937000
H	5.55266900	0.57812200	1.21191300
C	3.85340000	-0.64206100	0.72842400
C	3.01237400	-2.13521100	-2.70441200
H	3.25499700	-1.73858300	-3.69365300
H	3.34050400	-3.18161800	-2.67836400
H	1.92804600	-2.13904900	-2.58280400
C	3.33607800	-0.69856900	2.14018400
H	2.39799300	-0.15023200	2.24212800
H	3.12989300	-1.72966400	2.44469000

H	4.06136500	-0.26943000	2.83556700
C	0.98681900	0.74257200	-2.20421300
H	1.27499600	-0.11549100	-2.78828800
C	1.76374700	1.38119200	-1.18605800
H	2.75768300	1.10549100	-0.88421100
C	1.04092700	2.51956400	-0.70300100
C	-0.17277000	2.56424300	-1.43254500
H	-0.96030500	3.28944400	-1.29156800
C	-0.21122200	1.47951100	-2.34323300
H	-1.03954500	1.23272600	-2.99528700
C	1.59648400	3.62297900	0.18365800
C	2.66294000	3.08313400	1.15209900
H	3.49733200	2.62270500	0.61624600
H	2.24469400	2.33314300	1.82839900
H	3.06526600	3.89903700	1.76234200
C	2.26066600	4.67102900	-0.73877400
H	2.69172900	5.49167700	-0.15311900
H	3.05968100	4.21106400	-1.32843300
H	1.52902900	5.09262600	-1.43505000
C	0.47861900	4.30505000	0.99031400
H	0.88850600	5.11444700	1.60438900
H	-0.27981000	4.74217900	0.33329400
H	-0.01855500	3.59053800	1.65183300
Ti	-0.12079100	0.54433100	-0.12932100
C	-2.38540500	0.60581500	-0.19015200
C	-0.15868400	0.40346300	2.09655100
C	-2.23990900	0.37879700	1.18225800
H	-2.47868300	-0.27960200	-0.82118100
H	0.15748100	-0.58982300	2.43067000
H	0.71916400	0.93528900	1.64286600
H	-2.52379300	1.17698100	1.85862000
H	0.28314200	-4.73181400	0.57433200
C	-0.58919100	1.21675600	3.31221000
H	-0.93421800	2.21311000	3.00538600
H	-1.45094100	0.72505800	3.78529000
C	0.51995400	1.37219700	4.36192900
H	0.88098900	0.39308200	4.69645200
H	1.37650500	1.91731700	3.95080300
H	0.16620500	1.92117600	5.24219600
H	-2.30436500	-0.61606500	1.59583300
C	-3.13499600	1.83096400	-0.67619400
H	-2.85169600	2.70472600	-0.07413900
H	-2.86657200	2.06830500	-1.71189300
C	-4.65960500	1.64474800	-0.59668500

H	-4.94159600	1.43814700	0.44467800
H	-4.94059700	0.74934000	-1.16888500
C	-5.44604800	2.85213100	-1.11404400
H	-5.15810500	3.74333700	-0.53965600
H	-5.15483800	3.05446700	-2.15383700
C	-6.96234400	2.65903900	-1.03474900
H	-7.50154300	3.53459800	-1.41085800
H	-7.27971300	1.79218200	-1.62551800
H	-7.28350600	2.48768100	-0.00105800

$^{Ti}PR_{H21}$

N	-0.63632200	-2.53649600	0.31747700
N	1.51840100	-2.36079000	0.17158200
C	0.36101400	-1.57252300	0.10717300
C	-0.10799300	-3.82064800	0.46802800
C	1.23215500	-3.71511400	0.36676300
H	2.01330700	-4.45491800	0.43110900
C	-2.04358600	-2.30382200	0.27367200
C	-2.67698600	-2.17769700	-0.97335200
C	-4.05642600	-1.95095200	-0.98759300
H	-4.56371200	-1.83870900	-1.94128400
C	-4.77915300	-1.86735100	0.20156800
H	-5.84941500	-1.68669500	0.17189200
C	-4.13109300	-2.00749100	1.42772200
H	-4.69492700	-1.93513500	2.35328300
C	-2.75116700	-2.22675100	1.48344500
C	-1.88182500	-2.27299300	-2.24754300
H	-2.53147300	-2.18106100	-3.12121000
H	-1.34842600	-3.22798800	-2.30952800
H	-1.11974300	-1.48563300	-2.28097900
C	-2.02715200	-2.35188500	2.79932000
H	-1.65607700	-3.37002600	2.96281200
H	-1.15354600	-1.69355600	2.82720500
H	-2.68594900	-2.09453300	3.63216300
C	2.84710500	-1.86328800	0.05029900
C	3.57001800	-2.13367100	-1.12419100
C	4.87008800	-1.62835800	-1.22947700
H	5.43679200	-1.81144700	-2.13794000
C	5.43255900	-0.88238100	-0.19555700
H	6.43917800	-0.48814400	-0.29620900
C	4.70626800	-0.64840800	0.97226900
H	5.15199900	-0.08412900	1.78626200
C	3.40761200	-1.14294300	1.11993100
C	2.95528500	-2.93184600	-2.24535800

H	3.47037200	-2.73420200	-3.18874900
H	3.01711700	-4.00976000	-2.05303000
H	1.89637800	-2.69209200	-2.36397500
C	2.63607500	-0.93890300	2.39660900
H	1.70977300	-0.38292900	2.22452800
H	2.33801400	-1.90029800	2.82902700
H	3.23454500	-0.39761300	3.13266500
C	1.15599400	0.29111400	-2.52288500
H	1.27852300	-0.66075500	-3.01666700
C	2.08351900	0.91391200	-1.63283700
H	3.02834200	0.49443900	-1.32128400
C	1.57325400	2.19465500	-1.24287600
C	0.32486600	2.34228600	-1.90110700
H	-0.35089600	3.17840900	-1.79133300
C	0.06727000	1.18181800	-2.67198000
H	-0.83963400	0.99316900	-3.23206100
C	2.34266400	3.26317600	-0.48261400
C	3.13578400	2.64495600	0.68213300
H	3.83273400	1.87943900	0.33370300
H	2.46625600	2.17805700	1.41159600
H	3.71368700	3.41539400	1.20475300
C	3.33868800	3.92428600	-1.46173300
H	3.91868400	4.70820300	-0.96023300
H	4.03649300	3.18251100	-1.86203500
H	2.80746600	4.37526900	-2.30549500
C	1.39649500	4.34046100	0.06991800
H	1.96291700	5.09569300	0.62503600
H	0.85989600	4.85353500	-0.73386300
H	0.65563000	3.90424300	0.74457200
Ti	0.19025200	0.47642900	-0.38551000
C	-1.79660100	1.09239800	0.03946600
C	-0.49527800	1.52898800	2.18416900
C	-1.60891600	2.02069400	1.24984900
H	-2.11094900	0.11136200	0.43608700
H	-0.74835600	0.53505000	2.57057100
H	0.45245200	1.42442500	1.60767300
H	-1.36702100	3.03601500	0.90667100
H	-0.74562300	-4.67509500	0.62782500
C	-0.18145400	2.46501800	3.35359700
H	0.09440200	3.45213900	2.96209200
H	-1.10179800	2.61124700	3.93374700
C	0.93095100	1.94332600	4.26516800
H	0.67805400	0.95803200	4.67315900
H	1.87347400	1.84138100	3.71678200

H	1.10797900	2.61933100	5.10751900
H	-2.52906400	2.11507100	1.84991500
C	-2.85746400	1.54962200	-0.95312200
H	-2.54974800	2.49348900	-1.42702200
H	-2.91883500	0.81184500	-1.76467200
C	-4.26891200	1.72864000	-0.36891600
H	-4.28774200	2.59160400	0.31035100
H	-4.51336800	0.84997200	0.24112700
C	-5.34742000	1.90721500	-1.44012600
H	-5.10488000	2.77888800	-2.06340500
H	-5.32487700	1.03834400	-2.11276100
C	-6.75401600	2.06507400	-0.85801900
H	-7.50975300	2.17886900	-1.64253500
H	-7.02731200	1.19120100	-0.25484300
H	-6.81475000	2.94397200	-0.20607300

^{Ti}IM$_{H12}$

N	2.89457400	0.31955900	0.35727500
N	1.76451100	2.13582300	0.35609500
C	1.59937400	0.77723500	0.19352200
C	3.79854800	1.34874400	0.61021400
C	3.08760900	2.49452000	0.61095000
H	3.37313800	3.52201300	0.76584000
C	3.35181000	-1.04235700	0.41995600
C	3.93672100	-1.63186800	-0.71017100
C	4.37669100	-2.95795900	-0.60137600
H	4.81568000	-3.43865200	-1.47103300
C	4.26078300	-3.65408900	0.59728000
H	4.59492900	-4.68523900	0.66034900
C	3.75056700	-3.01719200	1.72797700
H	3.70380300	-3.54466200	2.67600600
C	3.31318200	-1.69203300	1.66657400
C	4.12217700	-0.89391700	-2.01303200
H	3.46747300	-1.29862900	-2.79037200
H	5.15372700	-1.00338500	-2.36242000
H	3.90751900	0.17077600	-1.91635800
C	2.89872500	-0.96147800	2.91824100
H	3.74699800	-0.39378700	3.32073600
H	2.08969700	-0.25792300	2.72941000
H	2.57178800	-1.66432700	3.68753000
C	0.76319100	3.16376500	0.31382100
C	0.52857400	3.82333300	-0.89902600
C	-0.40262100	4.86641900	-0.90194100
H	-0.60850700	5.38985900	-1.83087100

C	-1.06803000	5.22870900	0.26773300
H	-1.79168900	6.03780000	0.24938100
C	-0.81550000	4.55225400	1.45958600
H	-1.34594900	4.82917100	2.36553300
C	0.10906300	3.50534900	1.50425500
C	1.21814300	3.38130900	-2.16344300
H	0.99709800	4.06455600	-2.98647900
H	2.30461100	3.33007100	-2.03971500
H	0.88173100	2.37950700	-2.45018700
C	0.37405300	2.73820800	2.77338500
H	-0.19453800	3.15705900	3.60648100
H	0.08782700	1.68753300	2.65571500
H	1.43630800	2.75129900	3.03935000
C	0.78835800	-0.50573200	-2.45798400
H	1.61663200	0.17068900	-2.59791000
C	-0.55434900	-0.26560500	-2.84851100
H	-0.92201400	0.64061100	-3.30900000
C	-1.33572000	-1.41081200	-2.54542900
C	-0.46996200	-2.34134200	-1.91914500
H	-0.75600800	-3.31614100	-1.55086500
C	0.83746900	-1.80614300	-1.87578700
H	1.69686800	-2.29506600	-1.44562000
C	-2.76405900	-1.70480200	-2.97514800
C	-3.36291000	-0.51906800	-3.74788700
H	-2.79873500	-0.32197400	-4.66548100
H	-3.36255200	0.39322600	-3.14770300
H	-4.39826500	-0.73679900	-4.02997600
C	-2.73067800	-2.92693600	-3.92210900
H	-3.73767100	-3.15005600	-4.29308300
H	-2.08141400	-2.73708100	-4.78276000
H	-2.35558900	-3.81630100	-3.40721600
C	-3.64926200	-2.04160100	-1.76320900
H	-4.65708000	-2.32104900	-2.09148400
H	-3.23301000	-2.87695100	-1.19324000
H	-3.72593700	-1.18689200	-1.09222900
Ti	-0.32543300	-0.40924300	-0.39794400
C	-0.00538900	-1.37348500	1.52204300
C	-1.68059400	1.18396600	-0.20346100
C	-1.27739200	-1.77851400	0.98572800
H	0.83200700	-2.06537100	1.51147100
H	-0.00808700	-0.71633300	2.39205200
H	-1.39889200	1.37451500	0.85182200
H	-1.21531100	1.99409900	-0.78023000
H	-1.33376800	-2.78594200	0.57276300

H	4.84613700	1.15107500	0.76762600
C	-2.54049400	-1.38504000	1.72850200
H	-2.47279500	-0.33636200	2.04838800
H	-3.41756100	-1.45043400	1.07360900
C	-2.79913400	-2.25375000	2.97068400
H	-1.92744200	-2.19500400	3.63622400
H	-2.87802100	-3.30671900	2.66431300
C	-4.06137700	-1.85062000	3.73813300
H	-3.97760000	-0.79726000	4.03982800
H	-4.92707500	-1.90363800	3.06373300
C	-4.32002800	-2.71790500	4.97266800
H	-3.48256700	-2.65826000	5.67744300
H	-5.22624900	-2.40839100	5.50426800
H	-4.43959400	-3.77133700	4.69459800
C	-3.19613300	1.30655400	-0.33276200
H	-3.48319300	1.21564700	-1.38589000
H	-3.69199700	0.48268100	0.18814500
C	-3.75612100	2.62900400	0.21109800
H	-3.52440100	2.73850600	1.27697900
H	-3.31058600	3.48589900	-0.30495200
H	-4.84565100	2.68632300	0.09510800

$^{Ti}TS_{H12}$

N	1.75434800	-1.66520600	-1.25982300
N	-0.12157000	-1.19784500	-2.27918700
C	0.61144300	-0.85461400	-1.12999300
C	1.64297600	-2.53181900	-2.35462900
C	0.48507300	-2.24377900	-2.98259200
H	0.05768400	-2.63507700	-3.89160500
C	2.90449700	-1.59879400	-0.42251900
C	3.29911800	-2.72458300	0.33116100
C	4.40698900	-2.59952600	1.17601300
H	4.70538700	-3.45489800	1.77591800
C	5.11974400	-1.40586700	1.26438600
H	5.96870300	-1.32562900	1.93630300
C	4.75349700	-0.32492800	0.46409500
H	5.33179900	0.59442000	0.49191900
C	3.66370500	-0.40953800	-0.40491900
C	2.58275700	-4.04850200	0.23680900
H	2.78596400	-4.65772400	1.12113500
H	2.91325500	-4.62296300	-0.63729800
H	1.50357200	-3.92539400	0.14597800
C	3.34431000	0.71556900	-1.34888800
H	3.42738400	0.38212200	-2.39003700

H	2.31570100	1.05531200	-1.21712000
H	4.02421000	1.55788100	-1.19974500
C	-1.30322900	-0.53655800	-2.71784200
C	-2.54103400	-1.20475100	-2.65271100
C	-3.68740300	-0.51760600	-3.06653500
H	-4.65007600	-1.01811200	-3.00960600
C	-3.60787700	0.78762000	-3.54744700
H	-4.50811500	1.30844300	-3.85875600
C	-2.36911300	1.42048100	-3.63514900
H	-2.30080900	2.43018700	-4.03035700
C	-1.19960800	0.77070200	-3.23221100
C	-2.63667900	-2.63488800	-2.18654900
H	-3.64769800	-2.86324700	-1.83904200
H	-2.40489400	-3.33370000	-2.99980100
H	-1.93333700	-2.84202400	-1.38062700
C	0.14217200	1.43866200	-3.35905800
H	0.06100600	2.37435400	-3.91721600
H	0.56755400	1.65387700	-2.37476600
H	0.85925100	0.78635300	-3.86766000
C	-0.62514600	-2.51314800	0.97225700
H	-0.41691900	-3.19052800	0.15824600
C	-1.87020200	-1.86864100	1.24479300
H	-2.76369900	-1.95402300	0.64194000
C	-1.77387400	-1.15356300	2.48210400
C	-0.45013000	-1.30240500	2.93193900
H	-0.03165200	-0.85851500	3.82396800
C	0.26869300	-2.11231800	2.00866600
H	1.31702100	-2.36404700	2.08621700
C	-2.91610900	-0.52739200	3.26284100
C	-4.09452700	-0.16619800	2.34332500
H	-4.50796200	-1.05834300	1.86299400
H	-3.79840000	0.53206000	1.55940200
H	-4.89807400	0.29871200	2.92408700
C	-3.40788600	-1.57485400	4.28975700
H	-4.24579100	-1.17922600	4.87576300
H	-3.74299400	-2.48530400	3.78306200
H	-2.60438800	-1.84968000	4.97957600
C	-2.43958400	0.72244400	4.02411500
H	-3.27275100	1.18364800	4.56533300
H	-1.66919600	0.46788300	4.75802900
H	-2.01660600	1.46397400	3.34259200
Ti	-0.25780100	-0.29106900	0.68486800
C	1.23751000	1.00134200	1.63947400
C	-1.42190300	1.52031800	0.07858400

C	0.32834200	2.03254800	1.27809900
H	1.26285300	0.70640900	2.68276900
H	2.20205800	0.95415700	1.13857400
H	-1.16675000	1.70833700	-0.96323400
H	-2.02054600	0.57185500	0.06972600
H	-0.31638100	2.40846700	2.06884500
H	2.42716300	-3.22561200	-2.60861300
C	0.81469000	3.08746700	0.30107200
H	1.29527100	2.58319000	-0.54190800
H	-0.00680200	3.67836100	-0.11392100
C	1.82697500	4.04079400	0.95278100
H	2.65978800	3.45751200	1.36346700
H	1.35577300	4.54820100	1.80645900
C	2.36456400	5.08571500	-0.02916200
H	2.83030900	4.57053600	-0.88040500
H	1.52568600	5.66081100	-0.44387300
C	3.37625800	6.04016400	0.60940900
H	4.23812700	5.49133200	1.00533200
H	3.74994900	6.77386800	-0.11214200
H	2.92568700	6.59067700	1.44294100
C	-2.36176500	2.60338800	0.59734800
H	-2.66813300	2.37775800	1.62356500
H	-1.83923800	3.56543100	0.64657300
C	-3.60948600	2.77305900	-0.27993100
H	-3.33118100	3.04968400	-1.30144000
H	-4.17865700	1.83934700	-0.34727400
H	-4.27448400	3.54775100	0.11835000

$^{Ti}PR_{B12}$

N	-0.45999200	-2.50120000	0.38046000
N	1.67390000	-2.19658100	0.59416300
C	0.52222000	-1.52231600	0.16665900
C	0.07770000	-3.69253800	0.87383700
C	1.40711000	-3.50685400	0.99936300
H	2.18959200	-4.16105000	1.34811300
C	-1.83956700	-2.38277100	0.03579400
C	-2.21578500	-2.51285300	-1.31217000
C	-3.56797000	-2.36803600	-1.63320800
H	-3.87618400	-2.44910000	-2.67153700
C	-4.51503900	-2.11570400	-0.64099100
H	-5.56079400	-1.99525900	-0.90711500
C	-4.12274300	-2.01805600	0.69186800
H	-4.86179900	-1.82303200	1.46341900
C	-2.77715200	-2.15138700	1.05337400

C	-1.17712200	-2.76568800	-2.37102900
H	-1.64156200	-2.89795000	-3.35113200
H	-0.58572200	-3.65836700	-2.14008600
H	-0.47078500	-1.92699000	-2.41744000
C	-2.34512100	-2.04548400	2.49375400
H	-2.16057200	-3.03471800	2.92936700
H	-1.41361700	-1.48195600	2.58880200
H	-3.11429800	-1.55444000	3.09499600
C	2.98324200	-1.63546300	0.61487500
C	3.91142400	-2.03823300	-0.35910300
C	5.18780700	-1.46600000	-0.32901100
H	5.91352000	-1.75114600	-1.08526600
C	5.52679600	-0.52830000	0.64418600
H	6.51820200	-0.08563900	0.64849500
C	4.59589800	-0.16052600	1.61625800
H	4.86603800	0.55835200	2.38426600
C	3.31266600	-0.71354000	1.62297100
C	3.53759100	-3.05038400	-1.41162900
H	4.19907500	-2.97025000	-2.27789800
H	3.61518400	-4.07498700	-1.02831400
H	2.50538300	-2.91234400	-1.74093200
C	2.30884700	-0.35079500	2.68386100
H	2.72491700	0.37311200	3.38772900
H	1.40119900	0.07287700	2.24488100
H	1.99230600	-1.23829700	3.24295900
C	1.87021500	0.03634900	-2.48075200
H	2.18491300	-0.95370500	-2.77191300
C	2.48135800	0.86899000	-1.49454000
H	3.34038800	0.60301600	-0.89860000
C	1.79152400	2.12486200	-1.44750700
C	0.75903000	2.04890800	-2.41676400
H	0.01475800	2.80890100	-2.60653900
C	0.79831100	0.77499300	-3.03520700
H	0.08893700	0.41348300	-3.76926900
C	2.22693100	3.35510000	-0.66716400
C	2.77867100	2.95978500	0.71433800
H	3.62767300	2.27709100	0.63151300
H	2.01069600	2.46572900	1.31799300
H	3.11190000	3.84858400	1.26159200
C	3.34644000	4.06072700	-1.46444600
H	3.69854500	4.95592800	-0.93819800
H	4.19809700	3.38907800	-1.60990300
H	2.98368200	4.36232700	-2.45189400
C	1.05501000	4.33196000	-0.47599900

H	1.37935900	5.20606900	0.09893500
H	0.67273100	4.69107100	-1.43624600
H	0.22819900	3.85618100	0.05872200
Ti	0.36376900	0.40255300	-0.70720000
C	-1.68480200	0.93114000	-0.89818600
C	-1.06906600	1.56524200	1.47350100
C	-2.04950300	1.82033000	0.30840000
H	-2.05416700	1.35956000	-1.83364500
H	-2.17174000	-0.04749400	-0.78887700
H	-1.19499800	0.54030700	1.83937900
H	-0.03004000	1.65706700	1.07840700
H	-1.91996200	2.87018900	0.00586900
H	-0.54597400	-4.54716500	1.08039100
C	-3.50402500	1.63357000	0.79037700
H	-3.58199600	0.64187300	1.25617300
H	-3.74024700	2.36620900	1.57447900
C	-4.55904400	1.73226400	-0.31162400
H	-4.34012400	0.98802800	-1.08510100
H	-4.49583900	2.71552500	-0.80023200
C	-5.98284300	1.50744200	0.20486700
H	-6.02400700	0.53682900	0.71786300
H	-6.22509600	2.26309500	0.96452300
C	-7.03314800	1.53908100	-0.90761000
H	-6.82989300	0.76648400	-1.65831400
H	-8.04281000	1.37042700	-0.51847500
H	-7.03175600	2.50551100	-1.42438500
C	-1.15391900	2.54014700	2.65221400
H	-1.04457800	3.56584400	2.27598900
H	-2.15468700	2.48012500	3.09413400
C	-0.10661500	2.27337800	3.73522000
H	-0.19628000	1.25410100	4.12803200
H	0.90881600	2.38718200	3.34193700
H	-0.21680900	2.96598700	4.57573400

$^{Ti}IM_{B21}$

F	4.79102300	1.32921900	1.33373900
F	5.09940800	0.63068200	-1.69535200
F	3.70894300	-0.03549500	5.69940600
F	2.57704800	0.41400100	-2.84654900
F	2.58867200	2.92774400	1.19167600
F	2.78834300	-2.75405500	0.75298700
F	5.35767400	1.27844600	3.95692400
F	0.87304200	-1.24853100	2.16708000
F	4.72533600	-4.46614900	0.16424200

F	7.00688300	-1.10336400	-2.24828500
F	2.41777200	5.15698000	-0.21246400
F	6.87804600	-3.68118600	-1.34610700
F	2.39456800	2.68781000	-4.24017200
F	1.46428000	-1.29429000	4.74118800
F	2.33275800	5.10206300	-2.94351500
N	-0.12532400	0.11289400	-0.27023700
N	-0.56393400	-1.89929900	-0.92188100
C	-1.16877900	-0.73318300	-0.55800400
C	1.12323700	-0.51833000	-0.40901100
C	0.80156000	-1.78038600	-0.85234800
H	1.43706600	-2.61460200	-1.08761900
C	-0.35379500	1.54750900	-0.25788300
C	-0.36868900	2.29940300	0.92887400
C	-0.57088900	3.68534300	0.82117400
H	-0.56214200	4.28102400	1.72851600
C	-0.73095700	4.30176300	-0.41359900
H	-0.84776000	5.37861900	-0.47438900
C	-0.71046400	3.53404600	-1.57922100
H	-0.80863800	4.01486000	-2.54717800
C	-0.52452500	2.15110600	-1.52639400
C	-0.10747900	1.72828100	2.29363300
H	-0.75847300	2.19093300	3.04143600
H	0.92302300	1.95017400	2.57777500
H	-0.22881100	0.65184500	2.32717100
C	-0.48213500	1.35871000	-2.81115500
H	0.25153200	0.55387800	-2.77916800
H	-1.45151600	0.90315600	-3.04786300
H	-0.21630100	2.00938600	-3.64593900
C	-1.24478900	-3.12020700	-1.26411700
C	-1.41321600	-4.09267800	-0.26490200
C	-2.16387000	-5.22773800	-0.59744800
H	-2.32461600	-5.99386900	0.15486400
C	-2.66344500	-5.40108400	-1.88709400
H	-3.23404700	-6.29204000	-2.12892900
C	-2.39292900	-4.45980900	-2.88111300
H	-2.73731000	-4.62699900	-3.89659600
C	-1.66887000	-3.29986600	-2.59007400
C	-0.73327300	-3.97741500	1.07604100
H	-1.23255200	-4.59542900	1.82607200
H	0.30319200	-4.32591400	1.00118500
H	-0.67275000	-2.95301500	1.43954700
C	-1.34891700	-2.29124600	-3.66335300
H	-1.60376700	-2.68612100	-4.64829200

H	-1.90074800	-1.35472300	-3.52878400
H	-0.28655900	-2.03096800	-3.66292500
C	2.78966300	0.09563300	1.61447100
C	2.01815100	-0.59342000	2.54346900
C	2.28981900	-0.64205900	3.90705900
C	3.42359900	-0.00437900	4.39569300
C	4.25715800	0.67029300	3.50484900
C	3.93497600	0.69152200	2.15208200
C	2.66754100	1.54150800	-0.75815100
C	2.62572700	2.79520200	-0.15367500
C	2.53409100	3.99119700	-0.86363500
C	2.48352800	3.96810400	-2.25072300
C	2.52318800	2.74083700	-2.90500900
C	2.61243300	1.57841300	-2.15310400
C	3.78355600	-0.97066800	-0.49856700
C	4.92222000	-0.61960900	-1.23157500
C	5.95594700	-1.50821000	-1.52796300
C	5.89899000	-2.81822000	-1.06704600
C	4.80444800	-3.21361500	-0.30599900
C	3.80157400	-2.29012000	-0.03257200
C	-2.49090500	-0.88708500	2.06282300
H	-1.43755400	-1.02664600	2.24994800
C	-3.42196100	-1.90765900	1.72503900
H	-3.18350300	-2.95448800	1.60726800
C	-4.72502500	-1.34194800	1.59953600
C	-4.55879800	0.05679400	1.76749100
H	-5.35035200	0.78792300	1.71049000
C	-3.19377700	0.33457800	2.07192800
H	-2.76639300	1.30878500	2.25694600
C	-6.04332400	-2.09152700	1.60414200
C	-5.93655900	-3.46468200	0.91974600
H	-5.16616400	-4.08605300	1.38580300
H	-5.71094500	-3.38798400	-0.14390400
H	-6.88573400	-3.99768400	1.02115300
C	-6.38036800	-2.32478400	3.10006400
H	-7.33881900	-2.84564600	3.18575700
H	-5.61544500	-2.93642300	3.58742900
H	-6.45663800	-1.37740600	3.64153500
C	-7.16332000	-1.24457900	0.97618100
H	-8.09273700	-1.81920300	0.93982400
H	-7.36373400	-0.34696800	1.56921700
H	-6.91698800	-0.93247200	-0.04063100
B	2.59757800	0.07767500	-0.01951500
Ti	-3.24309000	-0.48401800	-0.11644200

C	-3.71955300	2.38849700	-0.25628900
C	-4.23929100	-1.59213500	-1.44015000
C	-3.91486200	1.47073500	-1.24171500
H	-2.70596500	2.72340900	-0.05848800
H	-3.40981400	-1.18499100	-2.08110700
H	-3.91790200	-2.60675400	-1.16213600
H	-4.92120900	1.18283700	-1.54064600
C	-5.52879500	-1.59549700	-2.24555700
H	-6.31103000	-2.09942700	-1.67387900
H	-5.87718500	-0.56810600	-2.40630700
C	-5.36435000	-2.29578200	-3.60329900
H	-5.05399200	-3.33554400	-3.46811300
H	-6.30762600	-2.28756200	-4.15658200
H	-4.60832200	-1.79616000	-4.21795700
H	-3.10473700	1.25973900	-1.93921500
C	-4.78282400	3.07737100	0.51706000
H	-5.76512900	2.62801700	0.33756500
H	-4.56419000	3.02059500	1.59112400
C	-4.80390300	4.57647600	0.11806600
H	-5.02882600	4.65995100	-0.95171900
H	-3.80338500	5.00105000	0.26444600
C	-5.83181100	5.36922000	0.93303800
H	-6.82468800	4.92232500	0.79556800
H	-5.59361800	5.27506900	2.00039600
C	-5.86832800	6.84654000	0.53691000
H	-6.60577600	7.39243800	1.13146300
H	-4.89478600	7.32294000	0.69336000
H	-6.13473700	6.96724600	-0.51828200

$^{Ti}TS_{B21}$

F	4.83115400	1.28370300	1.22168900
F	5.04864200	0.55496400	-1.80991300
F	3.82910800	0.02650200	5.63870300
F	2.49425200	0.34805800	-2.88592300
F	2.61475400	2.89859600	1.12639800
F	2.79425500	-2.77857800	0.75782400
F	5.45920700	1.27492400	3.83164600
F	0.89327600	-1.21301900	2.19862800
F	4.70307600	-4.51167000	0.13641700
F	6.92962400	-1.19928700	-2.39465600
F	2.39455400	5.11451200	-0.29203900
F	6.81335900	-3.76242700	-1.45013100
F	2.26457800	2.60896500	-4.29470500
F	1.54102700	-1.21154600	4.75859800

F	2.22973800	5.03361800	-3.01899700
N	-0.11845000	0.12303400	-0.26990900
N	-0.63769500	-1.90914900	-0.77820900
C	-1.19232000	-0.70101900	-0.47622800
C	1.10643700	-0.54926300	-0.39947900
C	0.73456300	-1.82331600	-0.76189800
H	1.33963500	-2.68561000	-0.97462000
C	-0.34160900	1.55592300	-0.27615400
C	-0.37590800	2.31074800	0.90898800
C	-0.62920600	3.68614300	0.79535100
H	-0.64372600	4.28713300	1.69900300
C	-0.81654500	4.28922000	-0.44236200
H	-0.97459300	5.36050800	-0.50732900
C	-0.78089000	3.51782700	-1.60492900
H	-0.91210600	3.98928700	-2.57366200
C	-0.55511500	2.14178500	-1.54549400
C	-0.09449900	1.74744300	2.27299800
H	-0.73031100	2.22189400	3.02610800
H	0.94276000	1.96147800	2.53875000
H	-0.22294800	0.67148300	2.31730400
C	-0.55736500	1.33364500	-2.82069000
H	0.13546000	0.49341800	-2.78690100
H	-1.55726700	0.93510500	-3.03622100
H	-0.26866400	1.95988900	-3.66663000
C	-1.35973700	-3.12588100	-1.04547300
C	-1.43132900	-4.09792000	-0.03250900
C	-2.20059300	-5.23905900	-0.29047400
H	-2.28398200	-6.00237800	0.47695100
C	-2.82763300	-5.41657300	-1.52311700
H	-3.41852800	-6.30852600	-1.70501800
C	-2.65675700	-4.47831200	-2.53956900
H	-3.08816500	-4.65474700	-3.51975200
C	-1.89813000	-3.32155700	-2.32709700
C	-0.65388900	-3.96596300	1.25309300
H	-1.05074900	-4.63591200	2.01870900
H	0.39719500	-4.23054900	1.09266000
H	-0.64337700	-2.94986200	1.64406800
C	-1.57255800	-2.39352800	-3.46999000
H	-2.26890900	-2.52894400	-4.29935100
H	-1.56771500	-1.34176600	-3.17991500
H	-0.56686200	-2.60832600	-3.84958500
C	2.82116200	0.08324800	1.57271000
C	2.05783900	-0.56941600	2.53402100
C	2.35912700	-0.59264700	3.89224400

C	3.51488300	0.03440600	4.34129300
C	4.33945800	0.67500900	3.41743300
C	3.98681500	0.67282700	2.07225100
C	2.64989100	1.49531200	-0.81256900
C	2.61870400	2.75429100	-0.21827500
C	2.49818400	3.94362300	-0.93557500
C	2.40833500	3.90736500	-2.32032400
C	2.43672000	2.67437100	-2.96439700
C	2.55275500	1.51888200	-2.20546800
C	3.76149000	-1.02108100	-0.55097200
C	4.87923100	-0.68759200	-1.32308400
C	5.89852700	-1.58673100	-1.63700000
C	5.84802500	-2.88940300	-1.15476800
C	4.77482800	-3.26689100	-0.35516700
C	3.78601300	-2.33320400	-0.06606900
C	-2.50961900	-0.90695800	2.10958000
H	-1.45614600	-1.04012600	2.30331100
C	-3.39758000	-1.88672000	1.60525900
H	-3.13048300	-2.89825900	1.34196600
C	-4.71529200	-1.33178400	1.53091700
C	-4.60197200	0.01888000	1.92745400
H	-5.41234500	0.72783000	1.96903100
C	-3.24938900	0.29186200	2.27327000
H	-2.85623300	1.23545000	2.62124300
C	-6.00424300	-2.10876000	1.32873600
C	-5.84743000	-3.25730900	0.31673400
H	-4.99811000	-3.90469200	0.55192700
H	-5.72475200	-2.89516500	-0.70577100
H	-6.74598100	-3.88015800	0.32814100
C	-6.33861100	-2.72107100	2.71352100
H	-7.28550200	-3.26654300	2.65878800
H	-5.56040900	-3.41976800	3.03394100
H	-6.43471700	-1.94321400	3.47622100
C	-7.15247600	-1.17735900	0.90633700
H	-8.05999300	-1.75969300	0.72657500
H	-7.38461100	-0.44621100	1.68611300
H	-6.91610800	-0.62967400	-0.01090500
B	2.59411600	0.03904600	-0.05695800
Ti	-3.20429700	-0.18672700	0.00205900
C	-3.75465200	2.03419300	-0.23212100
C	-4.22228000	-0.72879100	-1.67400800
C	-4.34465500	1.51228600	-1.37912600
H	-2.70597500	2.31729000	-0.31657400
H	-3.27078600	-0.71818700	-2.22844500

H	-4.15635300	-1.59268800	-0.94104100
H	-5.42710400	1.49425200	-1.45689900
C	-5.41313100	-0.91919700	-2.58605800
H	-6.32725600	-0.93596100	-1.98514800
H	-5.48533800	-0.04581000	-3.24345000
C	-5.33241300	-2.18246900	-3.45633300
H	-5.21843600	-3.08709400	-2.85375200
H	-6.24364400	-2.28149800	-4.05171300
H	-4.48482500	-2.12756300	-4.14380700
H	-3.80566400	1.50166800	-2.32022000
C	-4.55830500	2.85492800	0.75247500
H	-5.59459500	2.49920800	0.78307700
H	-4.15190000	2.77616500	1.76473500
C	-4.55133100	4.33960700	0.34072200
H	-4.99389700	4.43971000	-0.65890500
H	-3.51333200	4.68113400	0.25647000
C	-5.30681000	5.22440700	1.33704700
H	-6.34528800	4.87721500	1.41784700
H	-4.86320900	5.10285300	2.33425900
C	-5.28492100	6.70229500	0.94077900
H	-5.82870200	7.31367400	1.66603200
H	-4.25864000	7.08130800	0.88787200
H	-5.74804900	6.85620800	-0.03963200

^{Ti}PR$_{B21}$

F	4.92462300	0.78782900	1.24489500
F	5.02867800	0.38136400	-1.86494300
F	3.86744600	-0.86119600	5.51776500
F	2.45631500	0.55493800	-2.87934500
F	2.86099400	2.58709400	1.39928200
F	2.51165300	-2.98810200	0.39528400
F	5.58225300	0.42929100	3.82212500
F	0.79286800	-1.44979100	2.02742400
F	4.22529200	-4.81355500	-0.47038400
F	6.71552900	-1.46836700	-2.69425200
F	2.87714000	4.97015200	0.25207100
F	6.36837400	-4.09760500	-2.02950700
F	2.44605500	2.98208700	-4.01133400
F	1.46465100	-1.78565700	4.55644400
F	2.68513400	5.22732100	-2.46207100
N	-0.13849200	0.23789000	-0.21312900
N	-0.82205100	-1.69277700	-0.91015700
C	-1.27913800	-0.49336500	-0.44538800
C	1.02171900	-0.50012800	-0.47500400

C	0.54547900	-1.69815200	-0.96742800
H	1.08126100	-2.55739500	-1.32737900
C	-0.21391100	1.67949200	-0.04374200
C	-0.15988200	2.28693500	1.21847500
C	-0.23998800	3.68766400	1.27046400
H	-0.17268900	4.17461600	2.23835800
C	-0.36894700	4.45009000	0.11820800
H	-0.40059800	5.53252100	0.18168700
C	-0.44049400	3.82018600	-1.12487900
H	-0.52708100	4.41277800	-2.03019000
C	-0.37302900	2.43013200	-1.23016200
C	0.03350600	1.53851100	2.50708000
H	-0.58519300	1.97086100	3.29928000
H	1.07608400	1.62406600	2.82128900
H	-0.19116500	0.48148400	2.41397600
C	-0.49891700	1.78130800	-2.58666100
H	0.08560500	0.86561500	-2.67286100
H	-1.54681600	1.53573100	-2.80717500
H	-0.15393100	2.46189300	-3.36716400
C	-1.66334900	-2.80052400	-1.27472000
C	-1.67933500	-3.93409700	-0.44180200
C	-2.54919100	-4.97306800	-0.79249200
H	-2.59208900	-5.85736300	-0.16429200
C	-3.34058500	-4.89293300	-1.93765400
H	-4.00987800	-5.70936100	-2.18915100
C	-3.24485400	-3.78951800	-2.78304300
H	-3.81231300	-3.76333300	-3.70770600
C	-2.38149200	-2.72925700	-2.48183400
C	-0.77093100	-4.05877300	0.75563200
H	-1.13589300	-4.82891300	1.43854200
H	0.24131000	-4.34269000	0.44829200
H	-0.65952500	-3.12412400	1.30476800
C	-2.11920500	-1.64157400	-3.49507900
H	-2.89620400	-1.62176500	-4.26108900
H	-2.03130400	-0.64831900	-3.05265800
H	-1.16717500	-1.83226900	-4.00379400
C	2.81552900	-0.25544800	1.50722000
C	2.01095000	-0.94326500	2.40748700
C	2.32528000	-1.14667800	3.74735400
C	3.53842200	-0.67923400	4.23700600
C	4.40631600	-0.01580700	3.37040000
C	4.03766700	0.16571600	2.04183800
C	2.74925700	1.43187000	-0.69358400
C	2.84871400	2.60703900	0.04696400

C	2.85125700	3.87849700	-0.52444900
C	2.74664200	4.01436800	-1.90231300
C	2.63604600	2.87055700	-2.68674600
C	2.63945900	1.62777100	-2.07078200
C	3.61615800	-1.19431600	-0.74821100
C	4.74689200	-0.88515600	-1.51196900
C	5.66679500	-1.83733300	-1.95220200
C	5.49894300	-3.17488000	-1.61296100
C	4.40998500	-3.53464900	-0.82678900
C	3.52391600	-2.54858900	-0.40817200
C	-2.53867500	-1.02780900	2.18736700
H	-1.48379400	-1.19350200	2.34809100
C	-3.44708000	-1.94898800	1.60969900
H	-3.19315900	-2.92797800	1.23650100
C	-4.75203500	-1.37569700	1.60393500
C	-4.62325500	-0.06982700	2.14986400
H	-5.41899400	0.65242600	2.26183700
C	-3.26959000	0.14899300	2.50679800
H	-2.86723200	1.05403200	2.93757900
C	-6.03908600	-2.11346200	1.27796300
C	-5.80432400	-3.19441000	0.20855300
H	-5.09944400	-3.95596100	0.54995300
H	-5.40699800	-2.78327200	-0.72429800
H	-6.74615700	-3.69857300	-0.02431100
C	-6.48038400	-2.80191800	2.59427500
H	-7.40213400	-3.36728600	2.42699000
H	-5.71402600	-3.49492600	2.95236100
H	-6.66916200	-2.06506700	3.38016800
C	-7.15289900	-1.15186500	0.83197400
H	-8.07812600	-1.70892300	0.66320700
H	-7.36400600	-0.39485100	1.59265700
H	-6.90737400	-0.63856700	-0.10111400
B	2.56360700	-0.08887300	-0.11154500
Ti	-3.24469300	-0.08913300	0.21174600
C	-3.58479500	1.85287400	0.11710400
C	-4.72392600	0.16357000	-1.59551000
C	-4.76401800	1.52264400	-0.83054500
H	-2.65984500	1.96952200	-0.48699200
H	-3.77520200	0.04981400	-2.14474700
H	-4.84495200	-0.73354100	-0.93175000
H	-5.71064600	1.60253200	-0.28978400
C	-5.88677800	0.06233900	-2.60073900
H	-6.82278100	0.24759800	-2.06282100
H	-5.76990200	0.89028300	-3.30887400

C	-5.96357400	-1.26471200	-3.35135100
H	-6.10465100	-2.10871300	-2.67095100
H	-6.80751300	-1.25265300	-4.04542000
H	-5.05737600	-1.44611600	-3.93383300
H	-4.76200400	2.28102900	-1.62064700
C	-3.77957300	3.02565400	1.04425100
H	-4.64570500	2.84257400	1.69337100
H	-2.89804500	3.09977100	1.68745100
C	-3.96041500	4.36603800	0.30923800
H	-4.91543700	4.36933100	-0.23291800
H	-3.16815500	4.46699600	-0.44180900
C	-3.90590800	5.56427400	1.26082200
H	-4.68870200	5.46122100	2.02370100
H	-2.94829600	5.54350500	1.79719700
C	-4.06055200	6.90116100	0.53372000
H	-4.00997200	7.74061700	1.23249700
H	-3.26833400	7.03825300	-0.21061000
H	-5.02120400	6.96018000	0.01096500

^{Ti}IM$_{B12}$

F	4.99738900	1.26179600	1.34448600
F	5.13094400	1.09323800	-1.75945900
F	4.18551100	-0.79548200	5.49253300
F	2.55686800	1.02511500	-2.78446400
F	2.73428300	2.80018200	1.62437100
F	3.02608600	-2.70214100	0.22922600
F	5.72233200	0.79939100	3.88832200
F	1.15317700	-1.47707100	1.98046100
F	4.94504100	-4.24775300	-0.75085400
F	7.02458500	-0.48114500	-2.70555400
F	2.41044300	5.23246800	0.64423000
F	6.98709900	-3.17386500	-2.23665300
F	2.21924100	3.49982600	-3.74662900
F	1.89119200	-1.91530500	4.47826200
F	2.15598700	5.64480300	-2.04704400
N	0.01654800	0.22728600	-0.15972100
N	-0.44635900	-1.67628900	-1.07070100
C	-1.03475500	-0.58475500	-0.50352600
C	1.25849900	-0.35187200	-0.46513200
C	0.92066800	-1.53988500	-1.07150900
H	1.54760000	-2.31668500	-1.46954800
C	-0.24470400	1.63252500	0.09422600
C	-0.27749900	2.16720200	1.39322000
C	-0.59322700	3.52906600	1.52878400

H	-0.60916200	3.96008100	2.52505200
C	-0.82306500	4.33423800	0.42094300
H	-1.02679100	5.39205200	0.54931700
C	-0.76008400	3.78531800	-0.86070800
H	-0.91556400	4.41595100	-1.73030700
C	-0.48661400	2.42908200	-1.04852800
C	0.09588500	1.39957600	2.62993000
H	-0.52100600	1.70285400	3.48082300
H	1.13303000	1.62765900	2.88426100
H	0.02659700	0.32627700	2.49646400
C	-0.50748900	1.85997600	-2.44584900
H	0.17598900	1.02149000	-2.57318000
H	-1.51581200	1.51720700	-2.71302600
H	-0.22244500	2.62483400	-3.17048200
C	-1.13355700	-2.84955100	-1.54589500
C	-1.18376900	-3.97773400	-0.70969000
C	-1.93683700	-5.07205300	-1.15377000
H	-2.00514700	-5.95510300	-0.52592700
C	-2.56139100	-5.05155200	-2.39978300
H	-3.13769300	-5.91031500	-2.72879300
C	-2.41172300	-3.95166700	-3.24453200
H	-2.85342600	-3.96284100	-4.23588900
C	-1.67963200	-2.83118300	-2.83886100
C	-0.38736300	-4.05457200	0.56862200
H	-0.77548300	-4.83538700	1.22659600
H	0.65812800	-4.29775900	0.34787900
H	-0.35877000	-3.11280400	1.11367200
C	-1.44744600	-1.67563900	-3.77831900
H	-2.07747200	-1.76469800	-4.66513500
H	-1.64130700	-0.70767600	-3.31093300
H	-0.40339300	-1.65414200	-4.11024300
C	3.02572700	-0.03376800	1.53943300
C	2.31319000	-0.86943100	2.39103300
C	2.66411400	-1.12856200	3.71223500
C	3.82291700	-0.56237000	4.22902800
C	4.59979500	0.25534000	3.40939300
C	4.19728200	0.48677500	2.09860900
C	2.73960300	1.77949100	-0.54106200
C	2.69303700	2.90584900	0.27634300
C	2.51950700	4.20233400	-0.20575900
C	2.38606700	4.41630100	-1.57100700
C	2.43114100	3.32440600	-2.43210700
C	2.60116100	2.05331500	-1.90305200
C	3.91406600	-0.71470400	-0.77185000

C	5.00022700	-0.22032700	-1.50150300
C	6.02555800	-1.02323500	-2.00156900
C	6.01528800	-2.39220200	-1.76128700
C	4.97652300	-2.93347500	-1.01202200
C	3.98009800	-2.09158700	-0.53068300
C	-2.19463900	-1.22865400	2.08623700
H	-1.12862300	-1.36282900	2.18268400
C	-3.11369500	-2.17667500	1.55273500
H	-2.84687300	-3.14935200	1.16689900
C	-4.43794200	-1.65978100	1.63857600
C	-4.30632900	-0.34251400	2.15083100
H	-5.12573300	0.32401700	2.36822600
C	-2.93283600	-0.08039100	2.43133300
H	-2.52727100	0.84203500	2.82196100
C	-5.72217200	-2.46347800	1.53669400
C	-5.63826200	-3.57723000	0.47898100
H	-4.77406000	-4.22769800	0.64106900
H	-5.58006700	-3.18445400	-0.53595200
H	-6.53168400	-4.20451900	0.53973600
C	-5.89874000	-3.12889700	2.92671300
H	-6.83008300	-3.70284200	2.94688900
H	-5.07276200	-3.81186700	3.14566100
H	-5.94220300	-2.37973900	3.72270500
C	-6.93393500	-1.55454200	1.27247500
H	-7.83930000	-2.15947600	1.17402400
H	-7.10096800	-0.85985900	2.10165600
H	-6.81660700	-0.97116200	0.35690900
B	2.74180800	0.21001600	-0.06305400
Ti	-3.06793300	-0.35650500	0.10150000
C	-3.70075600	1.82012800	-0.48325200
C	-4.12121600	-1.14805800	-1.39701900
C	-4.60487300	2.07832000	0.49975500
H	-2.66121800	2.12524000	-0.35808400
H	-4.04339100	1.61718400	-1.49585500
H	-3.30823300	-0.64209800	-1.98211300
H	-3.82460200	-2.20499000	-1.34245100
H	-4.22408300	2.43797200	1.45504600
C	-5.44253700	-0.96061400	-2.12238700
H	-6.24031800	-1.45119300	-1.55979800
H	-5.69977800	0.10433900	-2.15911800
C	-5.40757300	-1.52609300	-3.55072800
H	-5.17500400	-2.59487500	-3.53914000
H	-6.37414000	-1.39087400	-4.04436100
H	-4.64624300	-1.02347700	-4.15603000

C	-6.08437800	2.05369400	0.34019400
H	-6.55812000	1.58111700	1.20750500
H	-6.36886300	1.48284500	-0.54846900
C	-6.61969000	3.50439800	0.22271500
H	-6.16194000	3.98597300	-0.64939900
H	-6.30821700	4.08230400	1.10183600
C	-8.14731000	3.53639800	0.09725800
H	-8.44930900	2.94449900	-0.77638700
H	-8.59050700	3.04569700	0.97323400
C	-8.68880400	4.96177200	-0.02961000
H	-9.77850400	4.95895100	-0.11856600
H	-8.42683900	5.56449200	0.84619700
H	-8.28284000	5.46244600	-0.91476200

$^{Ti}TS_{B12}$

F	4.96583000	0.62462700	1.28025700
F	4.87600500	1.12481900	-1.77957500
F	4.31414600	-2.24137300	4.94958800
F	2.25636900	1.42879500	-2.58386500
F	2.82181600	2.15931400	2.08159700
F	2.65290200	-2.90194000	-0.50232000
F	5.83479800	-0.41180600	3.60095200
F	1.00881000	-2.00182200	1.63125200
F	4.37910700	-4.28784900	-1.96137500
F	6.57813500	-0.29534800	-3.21032500
F	2.57589800	4.75957600	1.69126900
F	6.38134300	-3.01881700	-3.34454500
F	1.99760500	4.06768700	-2.94547300
F	1.88736500	-3.00434800	3.91080900
F	2.17113400	5.77666000	-0.81414200
N	-0.15732300	0.19695000	0.02747400
N	-0.83848700	-1.45428800	-1.18400400
C	-1.29068700	-0.47127500	-0.35407500
C	1.00828800	-0.36947400	-0.50857900
C	0.53014100	-1.38399200	-1.30606700
H	1.06144000	-2.08236600	-1.92644800
C	-0.31006500	1.52003200	0.60067400
C	-0.19470000	1.75637900	1.98071500
C	-0.40264100	3.06802100	2.43331700
H	-0.30550000	3.27250800	3.49479900
C	-0.69511000	4.10169600	1.55126900
H	-0.82458200	5.11281800	1.92251500
C	-0.79214200	3.84287400	0.18305900
H	-0.98686500	4.65514700	-0.50990900

C	-0.60729100	2.55305700	-0.31846000
C	0.18584400	0.70500600	2.98434500
H	-0.36755700	0.84422100	3.91744200
H	1.24854300	0.79931900	3.21694100
H	0.02363700	-0.30121900	2.61436600
C	-0.71461600	2.30874400	-1.80505600
H	-0.05609200	1.51240700	-2.14916800
H	-1.73666700	2.03964000	-2.09600200
H	-0.44821500	3.21222400	-2.35652500
C	-1.64815700	-2.46130200	-1.81896600
C	-1.58235300	-3.77871600	-1.33166600
C	-2.43055900	-4.72461800	-1.92093000
H	-2.41142400	-5.74692300	-1.55633000
C	-3.27337100	-4.37549300	-2.97487300
H	-3.92304600	-5.12343800	-3.41788900
C	-3.25041800	-3.08150900	-3.49207600
H	-3.85558900	-2.83157100	-4.35791300
C	-2.41874900	-2.10206300	-2.93702600
C	-0.60719100	-4.18808000	-0.25594300
H	-0.91541200	-5.12738500	0.20799800
H	0.39231600	-4.33967900	-0.67792100
H	-0.48570200	-3.43444800	0.52112400
C	-2.26424900	-0.76020300	-3.60591200
H	-3.08920100	-0.56773100	-4.29356700
H	-2.19532400	0.06394700	-2.89585900
H	-1.33634100	-0.73994400	-4.18921900
C	2.93558500	-0.58984900	1.35476100
C	2.23210800	-1.55461100	2.06514200
C	2.65573200	-2.10972300	3.26850600
C	3.88112400	-1.72417700	3.79771000
C	4.65021300	-0.78646800	3.10948700
C	4.17401600	-0.25884800	1.91423900
C	2.62408200	1.64923800	-0.24929100
C	2.69968600	2.56548100	0.79713600
C	2.57068900	3.94319600	0.62846600
C	2.35984800	4.46396300	-0.64093500
C	2.28457700	3.59231500	-1.72260600
C	2.41649300	2.22884200	-1.50230300
C	3.60456900	-0.79045200	-1.11734500
C	4.66934000	-0.20384500	-1.80928400
C	5.59585100	-0.92754100	-2.56019500
C	5.50447800	-2.31293000	-2.62762300
C	4.48576000	-2.95214500	-1.92962400
C	3.58932000	-2.18768300	-1.19143900

C	-2.26416900	-1.78235400	2.09359300
H	-1.19731500	-1.94809400	2.08811400
C	-3.21869900	-2.45675100	1.29545400
H	-3.00357100	-3.22954100	0.57455700
C	-4.52753500	-1.98877600	1.63321000
C	-4.34989400	-0.95725800	2.58285300
H	-5.14152600	-0.37101000	3.02695600
C	-2.96233700	-0.82145700	2.86735300
H	-2.51976100	-0.12166100	3.56160700
C	-5.83095400	-2.66696700	1.25521200
C	-5.82536200	-3.18290900	-0.19489800
H	-4.96506200	-3.82607600	-0.39853800
H	-5.82024100	-2.37489900	-0.92800200
H	-6.72649200	-3.77527200	-0.37540700
C	-5.94417700	-3.89173800	2.20118800
H	-6.88921600	-4.41248100	2.01949300
H	-5.12715900	-4.59886700	2.03176300
H	-5.91918300	-3.58423900	3.25037600
C	-7.03353800	-1.74275200	1.50660300
H	-7.95503300	-2.22632000	1.17197900
H	-7.15050900	-1.52178400	2.57158500
H	-6.94022700	-0.79140600	0.97543800
B	2.55370300	0.01338400	-0.12812900
Ti	-3.16264000	-0.20263100	0.63462200
C	-3.63732100	1.75087000	1.52802000
C	-4.49834400	0.13155500	-0.89808500
C	-4.54671700	1.92497300	0.46882300
H	-4.02587600	1.60331200	2.52662400
H	-2.69521500	2.29320500	1.47578800
H	-3.70607400	0.52869600	-1.55032200
H	-4.26529900	-0.97192800	-0.74355400
H	-5.59659900	1.72537700	0.66825900
C	-5.86594500	0.21349200	-1.54778700
H	-6.59538200	-0.30294600	-0.92040700
H	-6.18292600	1.25912100	-1.59189300
C	-5.88983000	-0.36930700	-2.96877000
H	-5.55186500	-1.40908100	-2.98369400
H	-6.90517500	-0.33549700	-3.37217500
H	-5.24174100	0.20612700	-3.63581600
C	-4.23991900	2.92852800	-0.61217100
H	-4.84198600	2.76559400	-1.50924500
H	-3.18827900	2.85544700	-0.89258900
C	-4.49490100	4.35544100	-0.08264700
H	-3.84756700	4.53006700	0.78452100

H	-5.53026700	4.44632900	0.26963400
C	-4.21701900	5.41394100	-1.15555300
H	-3.19373800	5.28210100	-1.53139300
H	-4.88248100	5.24740100	-2.01212400
C	-4.39444500	6.83916600	-0.62853200
H	-4.19138600	7.57565100	-1.41060600
H	-5.41598100	7.00469200	-0.27077200
H	-3.71244300	7.03994400	0.20463700

^{Ti}PR$_{B12}$

F	4.98112800	0.64797100	1.25384600
F	4.91669300	0.96903700	-1.84946600
F	4.24513700	-1.98020100	5.08163300
F	2.29766900	1.26146300	-2.67461200
F	2.85473200	2.26223800	1.93961100
F	2.63211800	-2.93759200	-0.33040800
F	5.81006500	-0.26182000	3.64103600
F	0.97659900	-1.88869300	1.72040000
F	4.34304800	-4.43692800	-1.69062300
F	6.60441100	-0.56282800	-3.17816900
F	2.65853400	4.84022300	1.39180300
F	6.36925200	-3.28563600	-3.14186700
F	2.08351900	3.87831500	-3.19724100
F	1.81521100	-2.76462700	4.06429800
F	2.27832600	5.71010700	-1.17061700
N	-0.13050000	0.22178500	-0.00090700
N	-0.81969600	-1.52081700	-1.08085000
C	-1.26830300	-0.48067400	-0.31775600
C	1.02382800	-0.38684600	-0.50068100
C	0.54021200	-1.45752300	-1.22705500
H	1.06806700	-2.18724400	-1.81340500
C	-0.25034000	1.58344500	0.48982600
C	-0.16668500	1.89031200	1.85579800
C	-0.36883300	3.22615300	2.23315500
H	-0.29300400	3.48567100	3.28440100
C	-0.63326300	4.21354700	1.29229200
H	-0.76154200	5.24415200	1.60593500
C	-0.71387400	3.88050000	-0.06001300
H	-0.90216100	4.65266800	-0.79900300
C	-0.53472400	2.56296800	-0.48652000
C	0.16203700	0.88375400	2.92260400
H	-0.40804600	1.08860000	3.83337400
H	1.22188700	0.95686700	3.17652100
H	-0.02478000	-0.13649000	2.60291200

C	-0.67545800	2.23353000	-1.95212100
H	-0.04147500	1.40419700	-2.26376100
H	-1.71276700	1.97533700	-2.19977000
H	-0.40274400	3.09651700	-2.56236900
C	-1.65581800	-2.54409000	-1.64986500
C	-1.57121000	-3.84507400	-1.11847500
C	-2.42510800	-4.81420100	-1.65582600
H	-2.39099900	-5.82355800	-1.25790100
C	-3.29675800	-4.50549700	-2.69992800
H	-3.94993900	-5.27274100	-3.10284200
C	-3.30391500	-3.22802300	-3.25348600
H	-3.93569900	-3.01212100	-4.10950000
C	-2.46566100	-2.22271900	-2.75358500
C	-0.57884600	-4.20721900	-0.04158300
H	-0.85507700	-5.15079500	0.43333500
H	0.42596700	-4.32877400	-0.45979200
H	-0.48625600	-3.44060500	0.72752000
C	-2.34229500	-0.90845400	-3.48536200
H	-3.26852200	-0.65339800	-4.00538000
H	-2.06089300	-0.08098900	-2.83504300
H	-1.55814100	-0.98392000	-4.24762000
C	2.92909000	-0.52501000	1.38029300
C	2.20253000	-1.43354600	2.13987200
C	2.60524200	-1.92240600	3.37851200
C	3.83185800	-1.52606500	3.89655300
C	4.62360000	-0.64471600	3.16106200
C	4.16789300	-0.18153600	1.93162300
C	2.66930900	1.61971100	-0.35807700
C	2.75089900	2.59458800	0.63320100
C	2.64315400	3.96111900	0.38102400
C	2.44487400	4.40751200	-0.91857100
C	2.35965400	3.47404700	-1.94673600
C	2.47124600	2.12474000	-1.64395100
C	3.61612600	-0.88227300	-1.07234100
C	4.69161100	-0.35526600	-1.79539300
C	5.61068200	-1.13839400	-2.49426300
C	5.50008300	-2.52395200	-2.47472000
C	4.46972700	-3.10360600	-1.74274400
C	3.58221000	-2.28093600	-1.05857100
C	-2.32478000	-1.70497900	2.18807800
H	-1.25991500	-1.88051300	2.23335300
C	-3.26638600	-2.44936300	1.43545600
H	-3.03766400	-3.28296700	0.79045800
C	-4.57084200	-1.91790600	1.66401100

C	-4.41020600	-0.80988800	2.53824000
H	-5.19793000	-0.15662100	2.88617600
C	-3.03603200	-0.67323900	2.85787200
H	-2.60348200	0.09283300	3.48520700
C	-5.87383800	-2.55649000	1.21601200
C	-5.67772600	-3.33961400	-0.09414200
H	-4.99088600	-4.17910800	0.03861300
H	-5.27791200	-2.71880400	-0.90033700
H	-6.63378700	-3.74983200	-0.43079300
C	-6.26826900	-3.55242500	2.33617200
H	-7.19239400	-4.07079700	2.06316800
H	-5.48719300	-4.30269400	2.48754800
H	-6.43401200	-3.03479600	3.28525400
C	-7.00218100	-1.52076500	1.06693000
H	-7.93387600	-2.02572500	0.79878000
H	-7.18226200	-0.98217800	2.00161100
H	-6.79698300	-0.78284100	0.28589900
B	2.57380600	-0.00262000	-0.14013500
Ti	-3.17161500	-0.28739800	0.57720600
C	-3.56142700	1.59244500	1.09805900
C	-4.67959700	0.49522300	-1.01076300
C	-4.63458400	1.71284000	-0.00934900
H	-3.86845300	1.97619300	2.06458700
H	-2.61415700	2.05085800	0.78545600
H	-3.72051200	0.37545300	-1.55016300
H	-4.91238700	-0.47399400	-0.49291000
H	-5.61649500	1.72051900	0.47573900
C	-5.80019200	0.62520000	-2.06221900
H	-6.68745100	1.02970300	-1.56232700
H	-5.48453300	1.37534400	-2.79176800
C	-6.15456900	-0.67889200	-2.77596500
H	-6.63351600	-1.38966400	-2.09818600
H	-6.85252300	-0.48017200	-3.59299800
H	-5.27226600	-1.16431000	-3.19932100
C	-4.42623200	3.02499200	-0.79999000
H	-5.21941700	3.13164600	-1.54619600
H	-3.47882200	2.95141300	-1.34831800
C	-4.40123700	4.27533700	0.08227100
H	-3.60345800	4.18783800	0.82763700
H	-5.34570800	4.35222500	0.63825600
C	-4.17608000	5.55395000	-0.73105100
H	-3.25470300	5.44410800	-1.31856200
H	-4.98910500	5.67568400	-1.45823500
C	-4.07613800	6.79990400	0.15072300

H	-3.91233200	7.69905500	-0.44931200
H	-4.99240900	6.94851600	0.73179300
H	-3.24421500	6.71465700	0.85889900

V_H

N	0.27830000	1.90840500	0.04115500
N	2.31717600	1.23004500	-0.19857000
C	1.02551200	0.75938100	-0.02882400
C	1.08478800	3.04516300	-0.05244100
C	2.36563000	2.62548800	-0.19257100
H	3.28346800	3.17776600	-0.30425000
C	-1.14339400	1.91394100	0.22066500
C	-1.65593900	1.58607500	1.48415600
C	-3.04404500	1.51981000	1.62349100
H	-3.46485500	1.24204100	2.58490300
C	-3.88461500	1.78167600	0.54400000
H	-4.95935200	1.69295700	0.66184000
C	-3.34868500	2.12990700	-0.69219100
H	-4.00575100	2.32299600	-1.53481800
C	-1.96460600	2.20507400	-0.87652300
C	-0.73333900	1.29795000	2.63867800
H	-1.29992200	1.16973600	3.56405000
H	-0.01319600	2.11060800	2.78609300
H	-0.15636700	0.38697500	2.44777400
C	-1.38105900	2.58578000	-2.21422100
H	-1.06452700	3.63590300	-2.23284600
H	-0.50113800	1.98370400	-2.45621800
H	-2.11830000	2.44881400	-3.00869300
C	3.35627300	0.26853100	-0.34270900
C	4.44842400	0.26329200	0.54433500
C	5.36747600	-0.78571600	0.44501700
H	6.21204200	-0.80633900	1.12762800
C	5.19544900	-1.81731600	-0.47538900
H	5.90933900	-2.63339700	-0.51766300
C	4.09952800	-1.80362000	-1.33436200
H	3.96649000	-2.59822200	-2.06203400
C	3.16713000	-0.75842900	-1.29559400
C	4.59789100	1.31354200	1.61544900
H	5.31884300	0.98707200	2.36813700
H	4.95245600	2.27036600	1.21497500
H	3.64285800	1.50426800	2.11333500
C	2.01185700	-0.75222600	-2.27743400
H	2.30590000	-1.29702100	-3.17944500
H	1.10852000	-1.32051800	-1.94386200

H	1.71029500	0.25810900	-2.55422900
C	1.62001500	-2.13402200	1.72635300
H	1.63763500	-3.21381700	1.49505000
H	2.65102500	-1.81519500	1.93110400
H	1.01523100	-1.99580600	2.62851300
H	0.66728700	4.03780000	-0.00872800
V	0.78817000	-1.23915800	0.07389900
N	-0.86087500	-1.44182100	-0.00375900
C	-2.22514200	-1.50696800	-0.12153300
C	-3.02565700	-1.81262300	1.00856100
C	-2.83732300	-1.26526600	-1.37803300
C	-4.41307900	-1.84195000	0.86597100
C	-4.22822600	-1.30169400	-1.47552500
C	-5.02076200	-1.58177200	-0.36274800
H	-5.02531400	-2.07395500	1.73428200
H	-4.69393800	-1.10789600	-2.43898700
H	-6.10274400	-1.60746700	-0.45424100
C	-2.36822400	-2.09727300	2.33065300
H	-1.61572800	-2.88685900	2.23524500
H	-1.83817000	-1.21699800	2.70619500
H	-3.10639900	-2.40120300	3.07855800
C	-1.97595800	-0.98430500	-2.57669100
H	-1.28339800	-0.16268800	-2.37470600
H	-1.36010400	-1.85622600	-2.83170600
H	-2.58182800	-0.72585000	-3.44996400

^{V}IM$_{H}$

N	0.34540800	1.77188400	0.54930000
N	2.37862000	1.10274400	0.39942700
C	1.08757400	0.67119200	0.20635100
C	1.14777000	2.84644400	0.92718700
C	2.42980000	2.42352100	0.83694700
H	3.36588800	2.91787400	1.03857600
C	-1.08844500	1.83147000	0.60429500
C	-1.72743100	1.34938000	1.75305000
C	-3.12269100	1.40875200	1.79180700
H	-3.64548200	1.01615500	2.65821000
C	-3.84227400	1.94364700	0.72569900
H	-4.92645500	1.95392200	0.75749900
C	-3.17634300	2.43758800	-0.39300400
H	-3.74091500	2.84511300	-1.22610700
C	-1.78117500	2.39371500	-0.47337400
C	-0.92541600	0.77528700	2.89186200
H	-1.57571100	0.50509600	3.72677700

H	-0.17819000	1.48852700	3.25685900
H	-0.38662700	-0.12206600	2.57184600
C	-1.04199500	2.92594400	-1.67499300
H	-0.48203100	3.83685300	-1.43252900
H	-0.31798000	2.19653300	-2.05002300
H	-1.73835400	3.16480100	-2.48175800
C	3.54564000	0.29486200	0.19279700
C	3.94578400	-0.57294000	1.22100000
C	5.05568400	-1.38984100	0.98633000
H	5.37687400	-2.07942200	1.76102200
C	5.74899700	-1.32117000	-0.22121700
H	6.60298700	-1.96963500	-0.39171900
C	5.36350900	-0.41002600	-1.20184900
H	5.92203200	-0.33971400	-2.13051600
C	4.25966900	0.42793200	-1.00731300
C	3.23315600	-0.58073500	2.54878800
H	3.66246000	-1.33482400	3.21165800
H	3.31841700	0.39517200	3.04019200
H	2.16994900	-0.79558200	2.43270400
C	3.89432800	1.47347100	-2.03166100
H	4.25976700	1.19021600	-3.02163400
H	2.81607300	1.62756100	-2.09333000
H	4.34848100	2.43854400	-1.77551400
C	1.09621500	-0.67526800	-2.31687800
C	1.12809600	-2.69176000	0.90456400
C	0.73401500	-2.05295000	-2.24816600
H	0.44744500	0.03842700	-2.81583500
H	2.14835900	-0.41719100	-2.33974500
H	0.66266200	-3.62425300	0.55245200
H	2.21824700	-2.80487600	0.84071700
H	0.83580600	-2.55568500	1.95263200
H	-0.17795300	-2.42498300	-2.70517800
H	1.52954700	-2.79538900	-2.16393700
H	0.72277100	3.79095900	1.22459100
V	0.38495800	-1.25817400	-0.37301200
N	-1.26726400	-1.16618400	-0.30451000
C	-2.63732700	-1.22654300	-0.36282600
C	-3.36082800	-1.77740400	0.72664300
C	-3.33219500	-0.73692100	-1.49792900
C	-4.75532100	-1.76846100	0.68774000
C	-4.72713400	-0.74775800	-1.49480600
C	-5.44363800	-1.24582500	-0.40710500
H	-5.30725300	-2.18402300	1.52732000
H	-5.25687900	-0.35833200	-2.36083900

H	-6.52966800	-1.24363800	-0.42016800
C	-2.62094800	-2.36839400	1.89498500
H	-1.88757000	-3.10869400	1.56007200
H	-2.05808300	-1.60596000	2.43985900
H	-3.31276200	-2.84823500	2.59309500
C	-2.56468100	-0.22448100	-2.68413200
H	-1.76989400	0.45107500	-2.36710000
H	-2.07931200	-1.04327600	-3.22704500
H	-3.22241000	0.30146400	-3.38212700

$^{V}TS_{H}$

N	-0.12279500	1.94218300	0.15934900
N	-2.20178300	1.39838200	-0.04862900
C	-0.93568400	0.83030100	0.03417800
C	-0.85944900	3.12971000	0.15453500
C	-2.16202500	2.79086500	0.02939500
H	-3.05718400	3.38992600	-0.01030700
C	1.29633600	1.89688200	0.33993700
C	2.12985500	2.19850600	-0.74357100
C	3.51281400	2.11782100	-0.54746100
H	4.17797800	2.31480900	-1.38267400
C	4.03632500	1.76354900	0.69141200
H	5.11000700	1.67602000	0.82003000
C	3.18370200	1.49441400	1.76085800
H	3.59487500	1.21398800	2.72605800
C	1.79793400	1.55787400	1.60535000
C	1.55783700	2.61028800	-2.07656600
H	2.28912600	2.45854200	-2.87398700
H	1.27650800	3.67079200	-2.08081900
H	0.65786100	2.04043100	-2.32000800
C	0.85854000	1.25839300	2.74350500
H	0.16557300	2.08966100	2.91698700
H	0.25274200	0.37614900	2.51086100
H	1.41275200	1.07612100	3.66754800
C	-3.37353900	0.59218300	-0.16421800
C	-3.77282800	0.16138900	-1.44064200
C	-4.86026300	-0.71419700	-1.52614700
H	-5.18237400	-1.06566200	-2.50212300
C	-5.53102100	-1.13023900	-0.37622400
H	-6.36902300	-1.81615800	-0.45768800
C	-5.14300100	-0.65514200	0.87563100
H	-5.68215200	-0.96427500	1.76634500
C	-4.06043300	0.22224200	1.00306400
C	-3.06634200	0.66064100	-2.67558200

H	-3.39166100	0.10779900	-3.56012100
H	-3.27893200	1.72301400	-2.84293200
H	-1.98188700	0.56050000	-2.57976900
C	-3.66046800	0.78757100	2.34147500
H	-4.07316800	0.18844600	3.15678700
H	-2.57476400	0.82563600	2.44770200
H	-4.03427300	1.81188300	2.45734000
C	-1.36400600	-1.84386900	1.75938600
C	-1.56388800	-2.56087100	-1.10916800
C	-1.85963000	-2.85218700	0.82490100
H	-0.50203200	-2.14218300	2.35340000
H	-2.11151300	-1.28946000	2.31616500
H	-0.74183900	-3.18903100	-1.46652300
H	-2.52182900	-2.96886000	-1.42935300
H	-1.54127000	-1.53429500	-1.64462000
H	-1.39556500	-3.83562400	0.83741800
H	-2.93939200	-2.89941400	0.71714900
H	-0.37847600	4.08970400	0.24623900
V	-0.56076800	-1.15496900	-0.04874200
N	1.10586200	-1.24035400	-0.11582100
C	2.46023900	-1.35789500	-0.28799000
C	3.03768800	-1.11983600	-1.56200700
C	3.28985000	-1.70869000	0.80744000
C	4.42445700	-1.17809100	-1.70175500
C	4.67114600	-1.76449500	0.62256900
C	5.24618100	-1.48867200	-0.61840300
H	4.86413400	-0.98055400	-2.67675100
H	5.30442500	-2.02535200	1.46738500
H	6.32439500	-1.52878800	-0.74306000
C	2.14437600	-0.83042600	-2.73608100
H	1.59652900	-1.73060000	-3.04414300
H	1.38831200	-0.08546100	-2.47781400
H	2.71917500	-0.47517600	-3.59651600
C	2.66287100	-2.01978900	2.13790700
H	2.05054100	-1.18516900	2.48847800
H	1.99130100	-2.88369100	2.06394500
H	3.42432700	-2.24023600	2.89175800

$^{V}PR_{H}$

N	0.17258600	1.98266900	0.05184300
N	2.23600100	1.37594900	-0.13467000
C	0.95563600	0.84884700	-0.07385400
C	0.94161000	3.14625400	0.06849700
C	2.23488400	2.77049600	-0.04731800

H	3.14766100	3.34235100	-0.08539500
C	-1.25639900	1.97665700	0.14706400
C	-1.84504800	1.84777400	1.41053800
C	-3.23999800	1.80264700	1.47777800
H	-3.72016300	1.67672800	2.44347500
C	-4.01201000	1.89201900	0.32166200
H	-5.09235400	1.81843800	0.38621300
C	-3.39988500	2.04030200	-0.92036300
H	-4.00382800	2.09638700	-1.82080600
C	-2.00732600	2.09120500	-1.02838100
C	-0.98648800	1.75520200	2.64556300
H	-1.59853100	1.58457200	3.53419600
H	-0.40790100	2.67310000	2.80066400
H	-0.26564600	0.93578400	2.55859900
C	-1.31847900	2.25620700	-2.35855200
H	-0.78757100	3.21336000	-2.41969500
H	-0.57375700	1.46904600	-2.51200600
H	-2.03904400	2.21653400	-3.17865200
C	3.41564100	0.57057300	-0.16954200
C	4.10052600	0.34018200	1.03558200
C	5.25744500	-0.44284200	0.99309100
H	5.80198500	-0.63538700	1.91275300
C	5.70237400	-0.98879300	-0.21060600
H	6.59818600	-1.60199800	-0.22752300
C	4.99941700	-0.75580300	-1.39045200
H	5.34770500	-1.18507900	-2.32512700
C	3.84440900	0.03421500	-1.39300600
C	3.58125300	0.90514700	2.33317300
H	4.16204700	0.53007400	3.17885000
H	3.62760400	1.99924700	2.34660000
H	2.53130600	0.63514900	2.48488300
C	3.07708900	0.28857100	-2.66480300
H	3.69155900	0.06237200	-3.53947500
H	2.18389900	-0.34527800	-2.71280400
H	2.74003900	1.32642300	-2.72976900
C	1.63612900	-2.72260100	-0.78369700
C	1.42497300	-2.37561700	1.71030700
C	2.35896200	-2.84841500	0.57464300
H	0.97581000	-3.58420400	-0.95326800
H	2.34007100	-2.66534900	-1.62207400
H	0.54642900	-3.01749900	1.80778800
H	1.93423000	-2.34087200	2.68055200
H	1.08458700	-1.30477300	1.61215300
H	2.68933300	-3.87208800	0.80672100

H	3.26132600	-2.23164900	0.58090500
H	0.48682000	4.11962900	0.15505100
V	0.42719000	-1.10894200	-0.25660600
N	-1.24298400	-1.14970400	-0.17521400
C	-2.59870000	-1.34350900	-0.15073700
C	-3.26860600	-1.53773600	1.08435400
C	-3.34022000	-1.32282900	-1.36001400
C	-4.65969300	-1.63698300	1.09296700
C	-4.72996500	-1.42594200	-1.30523700
C	-5.39802900	-1.56660900	-0.08848300
H	-5.16982400	-1.77170100	2.04400400
H	-5.29525300	-1.39821300	-2.23391300
H	-6.48116700	-1.64109200	-0.06325000
C	-2.46990900	-1.64722400	2.35292900
H	-1.87256300	-2.56764800	2.36454600
H	-1.76303600	-0.81936400	2.44531400
H	-3.12123600	-1.65634200	3.23170700
C	-2.61516000	-1.20144600	-2.67117600
H	-2.03918100	-0.27378900	-2.72388200
H	-1.89665800	-2.02075000	-2.79826800
H	-3.31408400	-1.22256600	-3.51240100

V_B

F	4.32217800	-0.40903000	1.51780600
F	4.61395200	0.64751000	-1.35624100
F	2.66212600	-3.26227200	4.86319200
F	2.22183100	1.54614500	-2.28349800
F	2.50333400	1.52096800	2.46877500
F	1.62201300	-3.02163500	-0.78618200
F	4.71825400	-1.84199200	3.75228200
F	-0.22417400	-1.82703200	1.45796900
F	3.21765100	-4.52636300	-2.28622000
F	6.17603000	-0.88196000	-2.83436700
F	2.86686400	4.12849600	2.44996700
F	5.52824600	-3.48873600	-3.34223100
F	2.63345000	4.19241600	-2.26917200
F	0.19075900	-3.21142700	3.66884500
F	2.94257500	5.52846500	0.09579600
N	-0.47832400	0.80952000	-0.44351700
N	-1.64536500	-0.74637200	-1.37310600
C	-1.75559800	0.48926200	-0.83990800
C	0.43603000	-0.20769900	-0.64460600
C	-0.33954400	-1.16689500	-1.30206300
H	-0.04966600	-2.13466800	-1.67062500

C	-0.54539400	2.12444700	0.16654300
C	-0.94449400	2.17929300	1.53442800
C	-1.69980300	3.29584900	1.94216500
H	-2.07411600	3.33154500	2.95976900
C	-1.96218100	4.35788800	1.05831200
H	-2.56536400	5.19790900	1.38591600
C	-1.43909800	4.33288000	-0.23505600
H	-1.63808300	5.15657300	-0.91299800
C	-0.73569200	3.21063100	-0.72387900
C	-0.61626000	1.08137400	2.49790200
H	-1.18029200	1.19909200	3.42274800
H	0.44920600	1.12573700	2.73446300
H	-0.80993000	0.09588800	2.07991300
C	-0.36567500	3.15787500	-2.18008300
H	0.08707200	2.21205700	-2.46316100
H	-1.24680900	3.33121900	-2.80431300
H	0.35184200	3.95034000	-2.40772600
C	-2.75034200	-1.46922300	-1.95713800
C	-3.40537600	-2.43047500	-1.17705300
C	-4.49128800	-3.08978300	-1.76485400
H	-5.02985300	-3.83219200	-1.18510000
C	-4.88065100	-2.80488400	-3.07125700
H	-5.72066800	-3.33307000	-3.51078600
C	-4.19785300	-1.84668100	-3.82112500
H	-4.50324200	-1.63333200	-4.84046100
C	-3.11462400	-1.15244700	-3.27506000
C	-2.96068200	-2.75455900	0.22461400
H	-3.76691400	-3.23933100	0.77770400
H	-2.09668200	-3.42718900	0.22323200
H	-2.66137500	-1.86022700	0.77218400
C	-2.36645500	-0.10955200	-4.06661000
H	-2.72869700	-0.07293800	-5.09527900
H	-2.48788800	0.88815800	-3.63057600
H	-1.29217200	-0.31952900	-4.09432100
C	2.04317700	-1.03412400	1.37108700
C	1.04468200	-1.79016000	1.97343300
C	1.21821400	-2.53065900	3.14007100
C	2.46772500	-2.55904800	3.74681400
C	3.51303400	-1.83242700	3.17790900
C	3.28215900	-1.10268800	2.01781900
C	2.35632300	1.37467800	0.07861100
C	2.54507700	2.10779100	1.25103900
C	2.74016800	3.49002000	1.28223700
C	2.77992200	4.20492200	0.09177300

C	2.63298700	3.51563200	-1.10980300
C	2.41960500	2.14483300	-1.08624600
C	2.97524400	-1.07055000	-1.05368000
C	4.18520500	-0.60910300	-1.58154300
C	5.04164700	-1.39614900	-2.35049200
C	4.72064100	-2.72472900	-2.60637200
C	3.54588700	-3.24635700	-2.07487200
C	2.72764500	-2.42112800	-1.31227300
B	1.97328300	-0.22139900	-0.05815100
C	-3.94950900	2.69495900	-1.54797300
H	-4.75446300	3.29111100	-1.09839600
H	-3.28839800	3.33605700	-2.13481400
H	-4.39190300	1.92318800	-2.17712800
V	-2.94860700	1.85598900	-0.01637700
N	-4.11767700	0.86277500	0.53624000
C	-5.01322100	-0.08071300	0.96901900
C	-6.05182100	-0.50752100	0.09477000
C	-4.86241200	-0.63612600	2.27046400
C	-6.90757800	-1.51122900	0.54156300
C	-5.75787000	-1.62383900	2.67020400
C	-6.76747400	-2.06603800	1.81378500
H	-7.69831200	-1.85696400	-0.11634000
H	-5.65978900	-2.05503800	3.66129500
H	-7.45240300	-2.84078600	2.14300000
C	-6.21131200	0.08568000	-1.27415700
H	-5.31883200	-0.08813400	-1.88193800
H	-7.06017500	-0.36469400	-1.79093700
H	-6.37903100	1.16527200	-1.22034000
C	-3.74812400	-0.20129500	3.17732000
H	-3.63197800	0.88621200	3.18386200
H	-3.92966100	-0.53233800	4.20156900
H	-2.79640400	-0.63575200	2.85452900

$^{V}IM_{B}$

F	4.34071100	-0.47315100	1.55449900
F	4.68647200	-0.07940800	-1.54233900
F	2.40943000	-2.67744100	5.23154500
F	2.33488500	0.98334000	-2.59075800
F	2.78600200	1.73469700	2.08306300
F	1.21076800	-3.13832900	-0.38762000
F	4.58335100	-1.57270500	3.99355700
F	-0.28647100	-1.59798200	1.55306000
F	2.54298200	-5.07761600	-1.61680600
F	5.97936200	-2.03719500	-2.74870500

F	3.42438600	4.24873700	1.60386000
F	4.95595500	-4.57058700	-2.82236000
F	3.01546100	3.53224400	-3.04576600
F	-0.02663300	-2.65306600	3.95216200
F	3.57855800	5.20285000	-0.95376800
N	-0.43051700	0.82539700	-0.32100200
N	-1.61710600	-0.64483400	-1.37534900
C	-1.69839500	0.55800500	-0.75135100
C	0.46089800	-0.20391600	-0.61471300
C	-0.32438200	-1.09506000	-1.33665500
H	-0.05525800	-2.03372700	-1.78576500
C	-0.23442900	2.16381900	0.20822000
C	-0.16744800	2.40056600	1.59868100
C	0.04453700	3.71949000	2.02135400
H	0.13182700	3.91691100	3.08486300
C	0.16230700	4.76298600	1.10632300
H	0.35065600	5.77216900	1.45704200
C	0.05209700	4.51181300	-0.26100400
H	0.16107000	5.32405100	-0.97252200
C	-0.13917700	3.21247100	-0.74406800
C	-0.27658300	1.31244500	2.62706200
H	-0.83472400	1.66595900	3.49857100
H	0.72285200	1.03226900	2.96526300
H	-0.75322000	0.42034600	2.23351200
C	-0.17439900	2.96988200	-2.23496500
H	0.10914200	1.95185300	-2.49718600
H	-1.16768900	3.16703400	-2.65197800
H	0.52370400	3.64085900	-2.73835400
C	-2.71701600	-1.31922200	-2.02061600
C	-3.33027500	-2.38362900	-1.34622200
C	-4.41609600	-3.00011500	-1.97912700
H	-4.92051000	-3.81927400	-1.47691500
C	-4.84199000	-2.58228800	-3.23739300
H	-5.68445800	-3.07396700	-3.71319400
C	-4.17858700	-1.54954100	-3.90124600
H	-4.49263100	-1.25250200	-4.89683100
C	-3.08940000	-0.90185900	-3.30949800
C	-2.81189300	-2.88295100	-0.02406200
H	-3.58084200	-3.44916000	0.50390200
H	-1.94771200	-3.54084600	-0.16963000
H	-2.47582400	-2.06988600	0.61702700
C	-2.30088500	0.14049900	-4.06188300
H	-2.81839200	0.43078500	-4.97811100
H	-2.12227200	1.03910200	-3.46841000

H	-1.31754300	-0.25174600	-4.34528800
C	2.02325500	-0.93753000	1.44051800
C	0.96816100	-1.55576800	2.10158100
C	1.06228800	-2.12546400	3.36722000
C	2.29003000	-2.13613400	4.01762000
C	3.39454000	-1.56614700	3.38488400
C	3.24163600	-0.99899900	2.12412500
C	2.60370300	1.19049100	-0.24164200
C	2.89957800	2.08676500	0.78220600
C	3.24124000	3.42133600	0.56598300
C	3.31289800	3.91248200	-0.73013200
C	3.03670300	3.05511400	-1.79082100
C	2.68491500	1.74002800	-1.52353800
C	2.83152800	-1.47074100	-0.95181000
C	4.08056100	-1.28046000	-1.55188000
C	4.79650300	-2.29718900	-2.18372800
C	4.28367800	-3.58885900	-2.21941500
C	3.05862400	-3.84146700	-1.61149800
C	2.38530600	-2.79542000	-0.99102500
B	2.00602800	-0.33232300	-0.09013300
C	-3.28787900	3.79198700	1.01996900
C	-4.01613800	2.36263100	-1.79616900
C	-4.17998500	3.06289600	1.74771500
H	-2.27208200	3.95866500	1.36946800
H	-3.62667900	4.40297600	0.18457300
H	-4.96290200	2.84800800	-1.53516100
H	-3.32785400	3.07729700	-2.26727600
H	-4.20958600	1.51642500	-2.45563000
H	-3.88477500	2.56293600	2.66488000
H	-5.23469700	3.02244800	1.49522500
V	-3.10537600	1.81094900	-0.12222500
N	-4.14783600	0.70177700	0.47932600
C	-4.91651500	-0.31740700	0.95320200
C	-6.05310900	-0.72560200	0.19310300
C	-4.55650600	-0.96277400	2.17414400
C	-6.80485300	-1.79217100	0.67209300
C	-5.35972400	-2.01352200	2.61033000
C	-6.46564300	-2.43003500	1.86814300
H	-7.66939800	-2.12500700	0.10718700
H	-5.11128100	-2.51624800	3.53961600
H	-7.07222200	-3.25530900	2.22727600
C	-6.39974300	-0.04515300	-1.09831500
H	-5.62135600	-0.22060700	-1.84632300
H	-7.34037300	-0.42959800	-1.49615200

H	-6.49917100	1.03652200	-0.96891500
C	-3.34109000	-0.56436600	2.96244300
H	-3.08670600	0.48753100	2.82550100
H	-3.49861700	-0.74469600	4.02833800
H	-2.46282400	-1.14917200	2.66493700

$^{V}TS_{B}$

F	4.41440000	-0.37794700	1.48126500
F	4.67133400	0.17625100	-1.59246300
F	2.68372400	-2.80562300	5.11678500
F	2.24458700	1.13851200	-2.55327300
F	2.75942700	1.73675700	2.13739100
F	1.40409500	-3.12091300	-0.48840300
F	4.77276900	-1.55909800	3.86810700
F	-0.16007200	-1.70307000	1.55734200
F	2.81254600	-4.93037300	-1.82721300
F	6.04296800	-1.65508200	-2.90908400
F	3.21098800	4.30489400	1.74616100
F	5.16208000	-4.23728200	-3.06490000
F	2.75174200	3.74131200	-2.91994500
F	0.21565500	-2.83940900	3.90641500
F	3.24112900	5.36528600	-0.77489200
N	-0.44876800	0.75727100	-0.25732800
N	-1.58108000	-0.70807500	-1.36654100
C	-1.70200000	0.46116000	-0.69683300
C	0.48469400	-0.22448500	-0.59932000
C	-0.26927800	-1.12056500	-1.34093000
H	0.02648800	-2.04270300	-1.80718100
C	-0.32635400	2.08994800	0.30613700
C	-0.25574900	2.29541100	1.70194400
C	-0.14267000	3.61544100	2.15681800
H	-0.05598400	3.79144900	3.22402000
C	-0.13141200	4.68909600	1.27045000
H	-0.02211100	5.70091700	1.64651200
C	-0.24566000	4.46679300	-0.10256400
H	-0.21488200	5.30454400	-0.79220500
C	-0.33285000	3.16862400	-0.61869200
C	-0.26521500	1.17361100	2.69843900
H	-0.81353700	1.46854900	3.59699500
H	0.76027800	0.94240800	2.99315400
H	-0.70322100	0.26741500	2.29175500
C	-0.35987100	2.96363400	-2.11696900
H	-0.03163700	1.96691500	-2.40604200
H	-1.35907400	3.12827400	-2.53623300

H	0.30772100	3.67827100	-2.60147500
C	-2.67446300	-1.39367600	-2.00636500
C	-3.25139400	-2.48754500	-1.34633800
C	-4.33201400	-3.11613900	-1.97609500
H	-4.80823200	-3.95817800	-1.48440100
C	-4.79018900	-2.68056600	-3.21699800
H	-5.62422300	-3.18667300	-3.69254000
C	-4.17227500	-1.60877100	-3.86227400
H	-4.51466000	-1.29361300	-4.84293600
C	-3.09414600	-0.94354200	-3.26968100
C	-2.70983200	-2.99314500	-0.03578500
H	-3.46451300	-3.57805500	0.49252500
H	-1.83524800	-3.63355400	-0.19572200
H	-2.38396100	-2.18038200	0.61204900
C	-2.38075800	0.17682200	-3.98653300
H	-2.72772000	0.25315900	-5.01846800
H	-2.54219100	1.15002300	-3.51075300
H	-1.29882800	0.01345700	-4.00368400
C	2.11809400	-0.94443200	1.40750800
C	1.10681800	-1.62925100	2.07256000
C	1.26170300	-2.24133700	3.31269200
C	2.50702100	-2.22349400	3.92873100
C	3.56822900	-1.58213600	3.29084500
C	3.35571600	-0.97578300	2.05761800
C	2.54943500	1.27143500	-0.20271400
C	2.81297400	2.14500200	0.84942500
C	3.05846300	3.50774500	0.68005600
C	3.06786500	4.05199700	-0.59640400
C	2.82957900	3.21828900	-1.68476200
C	2.56911700	1.87387700	-1.46331900
C	2.91410900	-1.34301200	-1.01831900
C	4.13491800	-1.05726400	-1.63779400
C	4.89102000	-2.00457600	-2.32787400
C	4.45083900	-3.32094200	-2.40565600
C	3.25770400	-3.66776700	-1.78109200
C	2.54203200	-2.68811300	-1.10217200
B	2.04053000	-0.28886000	-0.09936700
C	-3.46811900	3.25367400	1.37362000
C	-4.19810500	2.64602600	-1.45854900
C	-4.43772400	3.68129000	0.45935300
H	-3.78749200	2.71513000	2.26151100
H	-2.52809400	3.79810000	1.43076900
H	-5.24219100	2.79707100	-1.71868500
H	-3.53520100	3.32376100	-2.00275000

H	-3.97652900	1.57217300	-1.76155300
H	-5.47346600	3.38702200	0.58713200
H	-4.26578200	4.57610800	-0.13006000
V	-3.15754400	1.67299400	-0.04758800
N	-4.16091400	0.54773200	0.54069300
C	-4.91251300	-0.50134500	1.00651500
C	-6.01670500	-0.93924100	0.22759700
C	-4.54286000	-1.13496800	2.22226200
C	-6.73687600	-2.03722900	0.68676500
C	-5.30820800	-2.22472000	2.63675700
C	-6.38815700	-2.67670500	1.87899400
H	-7.58284800	-2.39300700	0.10754500
H	-5.04838100	-2.72649200	3.56353400
H	-6.96675100	-3.52822300	2.22206200
C	-6.37334000	-0.24352700	-1.05533300
H	-5.55773800	-0.31649800	-1.78160600
H	-7.25886100	-0.69380200	-1.50684600
H	-6.58624900	0.81772400	-0.88549400
C	-3.34465900	-0.69324900	3.01600100
H	-3.16741200	0.38128800	2.92965200
H	-3.47406000	-0.93287500	4.07379400
H	-2.43516900	-1.20478300	2.67925800

$^{V}PR_{B}$

F	4.83765500	0.32775100	0.52817000
F	4.14734600	0.01268800	-2.46652500
F	4.67698300	-1.24321100	4.95621100
F	1.43424700	0.45678300	-2.85275400
F	3.07346400	2.37836200	1.17521900
F	1.85840800	-3.11261900	0.31761600
F	6.06908100	-0.16025100	2.86764100
F	0.77238700	-1.34034600	2.35587900
F	3.11545100	-5.09779200	-0.91927600
F	5.37925600	-1.98718800	-3.66357900
F	3.05797000	4.75823400	0.03061900
F	4.90371600	-4.57907300	-2.93516600
F	1.46515400	2.87726700	-3.99728300
F	2.01426300	-1.82036300	4.64975000
F	2.28293300	5.06455300	-2.57297800
N	-0.43959300	0.52560900	0.17858700
N	-1.59877100	-1.26696300	-0.17277100
C	-1.72289300	0.03810800	0.18631500
C	0.50196300	-0.43488200	-0.15176700
C	-0.28574200	-1.56657900	-0.38413700

H	0.02009300	-2.56767000	-0.62935700
C	-0.35580400	1.96623900	0.33107200
C	0.01542800	2.53727700	1.56730200
C	0.05170200	3.93572700	1.64455300
H	0.35822500	4.39741300	2.57760000
C	-0.25971100	4.73139200	0.54302500
H	-0.18484200	5.81112300	0.61763700
C	-0.65950000	4.14457500	-0.65657600
H	-0.89485100	4.76595700	-1.51462800
C	-0.72982100	2.75194500	-0.79013100
C	0.41315900	1.71433000	2.75911000
H	0.12677800	2.21922900	3.68544400
H	1.49952300	1.59850500	2.76697600
H	-0.01461500	0.71304100	2.74389400
C	-1.16129400	2.14681700	-2.10804900
H	-0.72855800	1.16165400	-2.27708000
H	-2.25211900	2.05225200	-2.17032800
H	-0.84757500	2.78691700	-2.93430100
C	-2.69166000	-2.20506300	-0.23963800
C	-3.07476400	-2.84282600	0.94986400
C	-4.15108100	-3.73122500	0.87589300
H	-4.47363600	-4.24564100	1.77556900
C	-4.80590600	-3.96047400	-0.33382700
H	-5.64517500	-4.64727700	-0.36996500
C	-4.38251200	-3.32588900	-1.49867900
H	-4.88884000	-3.51906300	-2.43879700
C	-3.30206100	-2.43733000	-1.47769600
C	-2.35206500	-2.58143200	2.24728900
H	-2.74320500	-3.22099800	3.04051900
H	-1.27726200	-2.76520400	2.16155200
H	-2.46473200	-1.53898900	2.56495900
C	-2.79695800	-1.80329000	-2.74829100
H	-3.59731900	-1.72989000	-3.48760000
H	-2.38821400	-0.80456400	-2.58062600
H	-1.99569100	-2.40569300	-3.19129900
C	2.74888000	-0.44530900	1.32995200
C	2.10093200	-1.02344400	2.41648200
C	2.71492800	-1.28887500	3.63773400
C	4.06480100	-0.99776600	3.79626400
C	4.77031000	-0.44150000	2.73012000
C	4.10867000	-0.19205100	1.53320900
C	2.33287700	1.26494800	-0.80948400
C	2.73707600	2.41346000	-0.13328600
C	2.74341200	3.68492900	-0.70664700

C	2.34411900	3.84658400	-2.02626800
C	1.93704400	2.72904900	-2.74886800
C	1.93223400	1.48855500	-2.12816000
C	2.86453200	-1.43000500	-1.05060100
C	3.81478000	-1.22537400	-2.05667600
C	4.49574400	-2.25968300	-2.69839000
C	4.26275500	-3.57916600	-2.32800300
C	3.35206300	-3.83907400	-1.30965500
C	2.70219600	-2.77299800	-0.69810800
B	2.12959500	-0.23764000	-0.17666500
C	-3.22975100	1.34769800	2.65760400
C	-3.63307600	3.55634100	1.22963300
C	-3.94468000	2.71476000	2.51682100
H	-3.81593900	0.59833200	3.17760000
H	-2.21375200	1.42711700	3.05577500
H	-4.26171600	4.44951400	1.23820200
H	-2.59254400	3.89058000	1.17248300
H	-3.95632600	3.10424100	0.25094200
H	-5.02379600	2.58138400	2.59609900
H	-3.60515500	3.35452900	3.33788000
V	-3.06799300	1.36944200	0.71193400
N	-4.41559000	0.58662700	0.25822200
C	-5.51477400	-0.07294200	-0.22106200
C	-5.84822700	0.07472100	-1.59524300
C	-6.28204500	-0.88401800	0.65561000
C	-6.94195900	-0.63623900	-2.07955000
C	-7.36579500	-1.57346800	0.11674700
C	-7.69140900	-1.45810900	-1.23488200
H	-7.21524200	-0.54049700	-3.12560300
H	-7.96307400	-2.20584100	0.76570500
H	-8.54053800	-2.00490800	-1.63201100
C	-5.05302400	0.99142900	-2.48186900
H	-3.99315200	0.72481500	-2.48212900
H	-5.41243900	0.94906000	-3.51158800
H	-5.13114700	2.03279300	-2.14573500
C	-5.94241700	-0.97937200	2.11597800
H	-6.09029900	-0.01583600	2.61703700
H	-6.57740000	-1.71427200	2.61377300
H	-4.90081700	-1.27415000	2.25987300

$^{V}IM_{H21}$

N	-2.04082900	-0.26385800	-1.65100600
N	-0.01753400	0.30967900	-2.10216000
C	-0.79494000	-0.16119700	-1.07059000

C	-2.03019700	0.13795900	-2.98514800
C	-0.75949400	0.50367700	-3.26672100
H	-0.30291400	0.86939000	-4.17158500
C	-3.19780300	-0.84161700	-1.02631400
C	-4.21871500	-0.00612100	-0.54915500
C	-5.30685400	-0.60878800	0.09344600
H	-6.09835200	0.02055000	0.48977500
C	-5.38337300	-1.99252000	0.22433500
H	-6.23061500	-2.44238900	0.73285700
C	-4.38707500	-2.80332600	-0.31710300
H	-4.46453800	-3.88388100	-0.24544100
C	-3.28222400	-2.24323600	-0.96409500
C	-4.19028800	1.48667900	-0.75621700
H	-4.68115700	2.00337000	0.07208200
H	-4.72598100	1.75655200	-1.67478200
H	-3.17428400	1.86855300	-0.84454600
C	-2.23839900	-3.11534600	-1.61245300
H	-2.10214100	-2.84693800	-2.66539000
H	-1.26762500	-3.01206600	-1.12594100
H	-2.53154800	-4.16604300	-1.56062800
C	1.41876700	0.39817400	-2.10601700
C	2.03478800	1.65377600	-2.04734200
C	3.43332000	1.69081400	-2.05793700
H	3.93426000	2.65127500	-1.98295500
C	4.18068000	0.52033700	-2.14330700
H	5.26406400	0.56569000	-2.11612900
C	3.53932500	-0.71173300	-2.24444600
H	4.12230600	-1.62441600	-2.31509400
C	2.14535400	-0.79381400	-2.23835600
C	1.22720000	2.92510200	-1.99437200
H	0.78509000	3.15930100	-2.97057400
H	0.40615500	2.85071500	-1.28012200
H	1.85762600	3.76954200	-1.70632500
C	1.43841100	-2.11711800	-2.36783000
H	2.14675000	-2.91135700	-2.61434100
H	0.93930900	-2.38197600	-1.43151100
H	0.67030000	-2.08753400	-3.14778800
C	-1.43440000	1.09472100	1.37019800
C	-0.71097300	-2.52459800	1.47719400
C	-0.84653300	0.51432600	2.53257300
H	-2.45218600	0.76745400	1.15511300
H	-1.79678600	-2.62548200	1.31250200
H	-0.21404100	-3.20629600	0.76821600
H	-0.03872000	1.02336400	3.05191300

C	-0.36189100	-2.94095400	2.91296400
H	0.70064600	-2.75351300	3.11149100
H	-0.90914900	-2.30746700	3.62253100
C	-0.67661800	-4.41236900	3.21652200
H	-0.10606600	-5.07628000	2.55638900
H	-0.43166800	-4.67691800	4.25227700
H	-1.74047300	-4.62425600	3.05689500
H	-1.43848600	-0.14697300	3.16597300
C	-1.14991100	2.51128200	0.93449100
H	-0.09267900	2.73975200	1.09720800
H	-1.32435200	2.62190700	-0.14492500
C	-1.99684300	3.55815600	1.67515100
H	-1.80373400	3.47119600	2.75230700
H	-3.06281100	3.32796600	1.53815600
C	-1.72129100	4.99172900	1.21322500
H	-0.65400800	5.21385600	1.34851900
H	-1.91121500	5.06613200	0.13317600
C	-2.56060900	6.03557500	1.95356200
H	-2.34325900	7.05092400	1.60586600
H	-3.63174200	5.85472600	1.80730600
H	-2.36641400	6.00283200	3.03146600
V	-0.18174100	-0.60790300	0.92439800
N	1.46930300	-0.41116700	0.93285300
C	2.83083700	-0.29092500	1.07100400
C	3.65228200	-1.44834400	1.00507600
C	3.42916100	0.97972000	1.27352100
C	5.03840600	-1.30460700	1.08158300
C	4.81861000	1.07710400	1.34529300
C	5.62937500	-0.05184900	1.23723100
H	5.66114000	-2.19407800	1.02385400
H	5.26876200	2.05633800	1.48917900
H	6.70994100	0.04153800	1.29396700
C	2.56736000	2.20162900	1.41254100
H	1.87854200	2.28699800	0.57065200
H	1.95192800	2.15176100	2.31710200
H	3.17519500	3.10959200	1.46227000
C	3.03704500	-2.81292900	0.85365800
H	2.63960400	-2.96670100	-0.15371100
H	3.77755500	-3.59505900	1.04388900
H	2.19906600	-2.94552300	1.54153900
H	-2.92276700	0.11685100	-3.58864000

$^{V}TS_{H21}$

N	-1.59305900	-1.85666800	-1.39940700

N	0.47123300	-1.48888900	-1.90280400
C	-0.48236300	-1.14909500	-0.96158000
C	-1.32771100	-2.59637100	-2.55248400
C	-0.03337800	-2.36666300	-2.86550700
H	0.58042400	-2.72820300	-3.67414100
C	-2.84734700	-1.81515300	-0.71879100
C	-3.81016900	-0.88379900	-1.13791300
C	-5.00696800	-0.81131000	-0.41848000
H	-5.76242700	-0.09073200	-0.71754500
C	-5.22979200	-1.64367900	0.67811200
H	-6.16072800	-1.56903700	1.23230800
C	-4.26908700	-2.57966000	1.05854500
H	-4.45534800	-3.24127400	1.89973100
C	-3.06225900	-2.68992500	0.35785300
C	-3.53982100	0.01257700	-2.31835700
H	-4.34404700	0.74081100	-2.44596900
H	-3.45172600	-0.56572000	-3.24448600
H	-2.59788000	0.55126800	-2.18445700
C	-2.03564700	-3.73362300	0.71737500
H	-1.97065300	-4.50158000	-0.06217200
H	-1.04196900	-3.28863000	0.81769000
H	-2.29490300	-4.22624700	1.65764500
C	1.81934900	-1.00789800	-1.91652700
C	2.06491700	0.28045900	-2.41158300
C	3.38337400	0.73871300	-2.41092200
H	3.59433900	1.74169600	-2.76960800
C	4.41834000	-0.06422900	-1.93421400
H	5.43286400	0.31912100	-1.90917300
C	4.14913600	-1.34484600	-1.46292600
H	4.95419400	-1.96323000	-1.07806400
C	2.84207100	-1.84375200	-1.45306500
C	0.92711300	1.13269300	-2.90640900
H	0.37591700	0.63456200	-3.71213300
H	0.21498300	1.31683800	-2.09640900
H	1.29129700	2.09244200	-3.28081600
C	2.54603700	-3.24259000	-0.97405400
H	3.36569700	-3.62218500	-0.35953700
H	1.62661500	-3.27783800	-0.38447700
H	2.41278700	-3.93286400	-1.81613200
C	-1.82762400	1.47122800	0.37966000
C	-1.25004400	-0.37467600	2.51440300
C	-1.73772600	1.36961700	1.85811400
H	-2.77896500	1.08452500	0.00872800
H	-2.26386700	-0.76737600	2.64698000

H	-0.76522500	-1.31886600	1.93152300
H	-1.02787700	2.05738000	2.31990400
C	-0.54649800	-0.20334300	3.85419700
H	0.45274400	0.20745400	3.68345600
H	-1.10244300	0.53778500	4.44080000
C	-0.45773400	-1.51563800	4.64033200
H	0.12076700	-2.26441300	4.08954300
H	0.02992400	-1.36109400	5.60786000
H	-1.45400500	-1.93202500	4.82729600
H	-2.66080500	1.30623300	2.43072200
C	-1.48399000	2.81390900	-0.24201600
H	-0.52427500	3.16799500	0.15030000
H	-1.33836000	2.68740900	-1.32389700
C	-2.54792100	3.90066600	-0.01730200
H	-2.68702100	4.05227600	1.06240400
H	-3.51486200	3.54316100	-0.40090800
C	-2.20062100	5.23730500	-0.67826000
H	-1.23383400	5.58874700	-0.29270300
H	-2.05663800	5.07913500	-1.75601400
C	-3.26394600	6.31555100	-0.45601700
H	-2.98955300	7.26051900	-0.93655000
H	-4.23249400	6.00312700	-0.86325600
H	-3.40568200	6.51359500	0.61261900
V	-0.40070600	-0.01243600	0.73199700
N	1.19405900	0.47782200	0.85779100
C	2.53505400	0.68087300	1.06591600
C	3.32319100	-0.33180100	1.67551600
C	3.14577100	1.90183700	0.67847300
C	4.69187200	-0.12185600	1.84824700
C	4.51616800	2.07230100	0.87362700
C	5.29650700	1.06831600	1.44673900
H	5.29032100	-0.90692800	2.30468200
H	4.97748400	3.00798900	0.56646000
H	6.36367100	1.21523900	1.58536400
C	2.30787600	2.99352200	0.07471800
H	1.68523200	2.60720400	-0.73426700
H	1.62204700	3.41978500	0.81682200
H	2.93308000	3.80354100	-0.31236300
C	2.67599600	-1.60603600	2.14096600
H	1.96035300	-1.97382200	1.40214000
H	3.42188800	-2.38218700	2.33636700
H	2.11095800	-1.44726600	3.06771800
H	-2.08414300	-3.19988500	-3.02729800

$^{V}PR_{H21}$

N	-1.06306200	-2.04361100	-1.39943500
N	1.04594200	-1.62264700	-1.60458100
C	-0.07338000	-1.20244900	-0.90223100
C	-0.57166900	-2.92167400	-2.36727500
C	0.74706900	-2.65835900	-2.49154100
H	1.50571200	-3.09135800	-3.12265400
C	-2.40531100	-2.07072600	-0.91455500
C	-3.36532000	-1.25128800	-1.52555700
C	-4.66982100	-1.28582900	-1.02015200
H	-5.42902800	-0.65405300	-1.47147700
C	-4.99946500	-2.12097000	0.04657100
H	-6.01703900	-2.13718800	0.42540000
C	-4.02940100	-2.93727900	0.62801100
H	-4.28965900	-3.58842400	1.45745500
C	-2.71251600	-2.92357600	0.15736400
C	-2.98659000	-0.37317700	-2.68974000
H	-3.82355500	0.26394400	-2.98449400
H	-2.69125200	-0.97455800	-3.55657400
H	-2.13584000	0.26336400	-2.43133400
C	-1.64249700	-3.78171600	0.78210100
H	-1.23046500	-4.49592900	0.06121500
H	-0.80349600	-3.16951000	1.12937900
H	-2.03833000	-4.34033700	1.63299300
C	2.35655200	-1.04715900	-1.52945700
C	2.61874100	0.10925700	-2.28275600
C	3.91262300	0.62909300	-2.25023700
H	4.13672400	1.52477000	-2.82178800
C	4.90479100	0.03020600	-1.47364500
H	5.89862900	0.46478900	-1.43660600
C	4.61855900	-1.10736700	-0.73028200
H	5.38376700	-1.55619200	-0.10471000
C	3.33778100	-1.67326700	-0.75197600
C	1.52059900	0.77948000	-3.06355600
H	1.00855300	0.07699000	-3.72974400
H	0.77212200	1.17903100	-2.37040400
H	1.91708200	1.60054700	-3.66596800
C	3.05585100	-2.92488000	0.04067700
H	3.52810400	-2.86145700	1.02506300
H	1.98628200	-3.08691100	0.18092900
H	3.46026900	-3.81135800	-0.46283100
C	-1.91821800	1.52061100	0.12130600
C	-2.32411400	-0.06541400	2.10292700
C	-2.14978000	1.40132000	1.65668900

H	-2.76917000	1.03548100	-0.37323700
H	-3.27252000	-0.44457000	1.70851400
H	-1.59264200	-0.78386600	1.64346300
H	-1.31215400	1.85811600	2.20923700
C	-2.24944100	-0.28136600	3.61379900
H	-1.31045700	0.14083100	3.99350000
H	-3.05678800	0.29324000	4.08512900
C	-2.35306300	-1.75580600	4.00860300
H	-1.51983500	-2.33119100	3.58967400
H	-2.33241500	-1.88265700	5.09510700
H	-3.28173700	-2.19861300	3.63290200
H	-3.04361800	1.95458500	1.97817200
C	-1.74270900	2.94873700	-0.38094800
H	-0.91900500	3.42698800	0.16545500
H	-1.42023700	2.91673900	-1.43049200
C	-2.99891800	3.83042200	-0.28516300
H	-3.32874700	3.89977700	0.76092000
H	-3.82122300	3.34454700	-0.83116000
C	-2.79134600	5.24471000	-0.83483900
H	-1.97059200	5.72603200	-0.28609000
H	-2.45841600	5.17879500	-1.87973300
C	-4.04625200	6.11684400	-0.74999200
H	-3.87000200	7.12058500	-1.15084100
H	-4.87346900	5.67233400	-1.31545500
H	-4.38004600	6.22662600	0.28824600
V	-0.31763400	0.30355300	0.43821400
N	1.16033600	1.10574500	0.50536900
C	2.39780200	1.08731000	1.10437600
C	2.68377800	0.07250500	2.05613200
C	3.38013400	2.06234200	0.80291600
C	3.94704900	0.00476100	2.64071000
C	4.63178600	1.96544900	1.41160300
C	4.92887800	0.94143100	2.31421500
H	4.16046300	-0.78229200	3.36081300
H	5.39022800	2.70654200	1.16956400
H	5.91298400	0.88351200	2.76971400
C	3.04311100	3.18125500	-0.14427100
H	2.63450200	2.79280100	-1.08036900
H	2.27325000	3.83601600	0.28138400
H	3.92503000	3.78873100	-0.36830100
C	1.57696800	-0.88036700	2.40527000
H	1.16357600	-1.35991900	1.50016400
H	1.90370000	-1.68336600	3.07197200
H	0.75237300	-0.34604500	2.90265800

H	-1.21210500	-3.63244400	-2.86372700

$^{V}IM_{H12}$

N	-0.78507000	2.38392200	-0.02751400
N	1.33233100	2.28660800	0.31920900
C	0.23255200	1.48170400	0.13554900
C	-0.33027500	3.69933500	0.03840800
C	1.00340500	3.63878600	0.26269000
H	1.74656300	4.40918200	0.38734300
C	-2.17018500	2.04330600	-0.19791700
C	-2.94740900	1.83502500	0.94765700
C	-4.28763000	1.48436800	0.76579900
H	-4.90593900	1.28749300	1.63582000
C	-4.82396100	1.36083000	-0.51358800
H	-5.85624600	1.05244900	-0.63831700
C	-4.03094400	1.59868200	-1.63316700
H	-4.45057000	1.49271100	-2.62886700
C	-2.68531700	1.95169900	-1.49567300
C	-2.34609100	1.98442900	2.32135300
H	-3.07848500	1.74203400	3.09411300
H	-1.99207800	3.00712200	2.49442900
H	-1.48386100	1.32272100	2.44746200
C	-1.81411300	2.23130000	-2.69360200
H	-1.54660100	3.29303000	-2.75403900
H	-0.87892400	1.66755700	-2.64256300
H	-2.32973700	1.96332600	-3.61851000
C	2.65827000	1.78782400	0.54452000
C	2.99941300	1.36920600	1.84177400
C	4.26786200	0.81519800	2.03479500
H	4.54744400	0.46830300	3.02481500
C	5.17008500	0.71286000	0.97663900
H	6.14770600	0.27007400	1.14026500
C	4.82828000	1.19087600	-0.28529900
H	5.54053800	1.12759100	-1.10253700
C	3.56725000	1.74967800	-0.52408700
C	2.05572200	1.57590800	2.99885200
H	2.41514600	1.05420000	3.88785900
H	1.97400700	2.64277100	3.23871300
H	1.05131100	1.21436100	2.77737100
C	3.24309300	2.33669400	-1.87551200
H	3.70370900	1.74866200	-2.67360700
H	2.16962800	2.38469300	-2.05971100
H	3.63761200	3.35740800	-1.95151800
C	0.97612600	-0.40679000	-1.82283000

C	0.79770500	-1.39456100	1.95351900
C	1.21251700	-1.70235500	-1.27183900
H	0.18965300	-0.26746500	-2.55617200
H	1.80964400	0.27567600	-1.92935100
H	1.84182000	-1.11316400	2.15427600
H	0.17514300	-0.87529100	2.70304300
H	0.65801700	-2.55849800	-1.65322900
H	-1.00075200	4.53519900	-0.07591400
C	2.60868100	-2.00545400	-0.74486500
H	2.93999900	-1.20954900	-0.06276700
H	2.59503900	-2.92536500	-0.15004400
C	3.66263500	-2.13428900	-1.85612700
H	3.70732100	-1.18939700	-2.41356800
H	3.34214700	-2.89827700	-2.57754500
C	5.05532500	-2.47884100	-1.32164800
H	5.33199300	-1.74611600	-0.55290500
H	5.01809600	-3.45226500	-0.81445900
C	6.12790600	-2.50713600	-2.41279600
H	6.21245200	-1.53093100	-2.90512400
H	7.11271700	-2.76242600	-2.00728300
H	5.88523100	-3.24343500	-3.18729200
C	0.61992600	-2.91081700	2.12687600
H	-0.39514000	-3.20292600	1.83445400
H	1.28744600	-3.44091500	1.43690000
C	0.89503300	-3.40143400	3.55499100
H	1.91439500	-3.14341200	3.86621600
H	0.20560700	-2.93164700	4.26666600
H	0.78036800	-4.48874700	3.64404300
V	0.12696000	-0.64290300	0.14188400
N	-1.45634400	-1.08630900	-0.05657900
C	-2.75144300	-1.51145000	-0.21822600
C	-3.28352400	-1.69845700	-1.51966600
C	-3.56810700	-1.74660900	0.91854400
C	-4.62012800	-2.07029500	-1.66143400
C	-4.90231900	-2.10955600	0.72985300
C	-5.43743300	-2.26509000	-0.54824000
H	-5.02416600	-2.20467800	-2.66193200
H	-5.52858400	-2.27554400	1.60314500
H	-6.47806900	-2.54867600	-0.67555500
C	-2.99760900	-1.61358900	2.30432600
H	-2.31083300	-2.43649600	2.52960200
H	-2.41847800	-0.69522100	2.41169100
H	-3.79182700	-1.62191900	3.05655000
C	-2.40541400	-1.48648100	-2.72042500

H	-1.99909400	-0.47259000	-2.72713600
H	-1.54568800	-2.16466000	-2.71074800
H	-2.96149200	-1.64677500	-3.64846200

$^{V}TS_{H12}$

N	-1.19876700	-2.33785000	0.43106100
N	0.90642800	-2.57742000	0.01984200
C	-0.07423600	-1.59919900	0.11822000
C	-0.92038400	-3.70445000	0.51516700
C	0.39875400	-3.85565200	0.26288900
H	1.02574800	-4.73179300	0.22913500
C	-2.48549700	-1.77047600	0.69553800
C	-3.44358300	-1.76278700	-0.32420100
C	-4.68343000	-1.17656900	-0.05130700
H	-5.43183200	-1.12994900	-0.83637500
C	-4.95146200	-0.63021000	1.20048500
H	-5.90619300	-0.15008600	1.38710200
C	-3.98458000	-0.66745700	2.20364700
H	-4.19345400	-0.23181300	3.17623500
C	-2.73286900	-1.24110800	1.96931400
C	-3.13513300	-2.36904000	-1.66913200
H	-3.87636800	-2.06445200	-2.41146600
H	-3.13381700	-3.46498000	-1.62531300
H	-2.14545000	-2.06133300	-2.01826400
C	-1.66859000	-1.29082100	3.03547000
H	-1.37115900	-2.32302000	3.25302600
H	-0.76730500	-0.76194600	2.70732100
H	-2.02494200	-0.83474400	3.96220000
C	2.27575200	-2.27189000	-0.24547200
C	2.70396300	-2.15762600	-1.57740900
C	4.04008100	-1.80885800	-1.80741400
H	4.39269100	-1.70917400	-2.82996600
C	4.91677700	-1.60405500	-0.74307800
H	5.94973600	-1.33181000	-0.93826400
C	4.47430100	-1.75128000	0.57120100
H	5.16163600	-1.59733000	1.39784400
C	3.14470000	-2.08926200	0.84255600
C	1.75931100	-2.44303500	-2.71707900
H	2.20132100	-2.14651500	-3.67138300
H	1.52482300	-3.51256600	-2.77021400
H	0.81269400	-1.91145600	-2.58881200
C	2.64763900	-2.27081100	2.25288600
H	3.39409700	-1.93198300	2.97524600
H	1.72301500	-1.71076400	2.41205300

H	2.42767400	-3.32394000	2.46107700
C	1.47012400	0.95833700	1.31179500
C	1.43067300	1.19184300	-1.58496800
C	1.91853500	1.82715500	0.19868400
H	0.84162100	1.46228700	2.04570100
H	2.24414400	0.34488400	1.76713300
H	2.42110700	0.83243000	-1.87958000
H	0.79081500	0.23130500	-1.90847700
H	1.42625400	2.80105600	0.17140000
H	-1.68887900	-4.42273300	0.74964800
C	3.42246100	1.92716400	-0.02378100
H	3.83422700	0.91539900	-0.11183400
H	3.65000500	2.44594900	-0.96457200
C	4.12728300	2.65532000	1.12602700
H	3.88103700	2.15586900	2.07072800
H	3.73670100	3.67919200	1.20850600
C	5.64815500	2.69798100	0.94934500
H	6.02515600	1.66980700	0.86048000
H	5.89168300	3.19348900	-0.00028200
C	6.36338200	3.41099300	2.09901600
H	6.16071700	2.91561700	3.05527100
H	7.44832300	3.42472500	1.95287700
H	6.02533800	4.44940500	2.18877400
C	1.01092000	2.39007400	-2.42652000
H	0.05627800	2.77569000	-2.05804500
H	1.75680500	3.18308400	-2.28779000
C	0.89710000	2.04680000	-3.91550600
H	1.84224800	1.65135100	-4.30479300
H	0.12195600	1.29121700	-4.08077900
H	0.63410600	2.93113300	-4.50446700
V	0.19538900	0.38013500	-0.21729400
N	-1.33737300	1.05259900	-0.28669500
C	-2.55715300	1.67087900	-0.19571400
C	-2.96636900	2.29655800	1.01056400
C	-3.41877500	1.69125900	-1.32416400
C	-4.23435300	2.87325900	1.08243400
C	-4.67950600	2.27715500	-1.20846500
C	-5.09935700	2.85765200	-0.01171200
H	-4.54667000	3.34076100	2.01335200
H	-5.34014900	2.27827000	-2.07245000
H	-6.08583900	3.30589400	0.06335600
C	-2.95356800	1.10159600	-2.62716800
H	-2.17853100	1.72716300	-3.08789600
H	-2.50304700	0.11938000	-2.47480800

H	-3.78015700	1.01244300	-3.33840600
C	-2.02715200	2.33589100	2.18315500
H	-1.65293800	1.33756700	2.41895600
H	-1.14606700	2.95210500	1.96425600
H	-2.51860800	2.74854700	3.06905200

$^{V}PR_{B12}$

N	-1.41893900	2.31300300	0.17134300
N	0.70071200	2.65714600	-0.06962900
C	-0.24314000	1.63848400	-0.10903300
C	-1.20223600	3.67565600	0.37515000
C	0.12382800	3.89474000	0.22831200
H	0.71315700	4.79474000	0.29346200
C	-2.70430600	1.68762800	0.25703000
C	-3.10895500	1.15323100	1.48652000
C	-4.34393700	0.50130700	1.53978700
H	-4.66824100	0.05525700	2.47515200
C	-5.14092200	0.39360700	0.40246200
H	-6.07880600	-0.14930900	0.44914400
C	-4.72202900	0.95098500	-0.80314700
H	-5.33995700	0.85331800	-1.69048000
C	-3.49531900	1.61472900	-0.89512700
C	-2.21791500	1.27080700	2.69556900
H	-2.63781800	0.72775200	3.54549300
H	-2.08061000	2.31659900	2.99390200
H	-1.22310800	0.86742700	2.48282800
C	-3.01908800	2.22996000	-2.18587200
H	-2.96494000	3.32262100	-2.11434300
H	-2.01405200	1.87709800	-2.43683900
H	-3.69132900	1.97803000	-3.00925800
C	2.10053200	2.43156900	-0.24329800
C	2.91586100	2.34536600	0.89781300
C	4.27503100	2.07372900	0.71470500
H	4.92030300	1.98909800	1.58414800
C	4.80136900	1.90397600	-0.56516200
H	5.85783100	1.68763700	-0.69075700
C	3.97609400	2.00750200	-1.68259800
H	4.38869800	1.87360500	-2.67812500
C	2.60924500	2.27045500	-1.54145500
C	2.33387500	2.53440000	2.27578100
H	3.02762500	2.17889400	3.04141000
H	2.12126200	3.59014900	2.47923400
H	1.38900300	1.99528000	2.38369300
C	1.70337000	2.34023200	-2.74345100

H	2.28256800	2.48587100	-3.65828900
H	1.13274700	1.40981100	-2.85736000
H	0.97476400	3.14999300	-2.65302300
C	1.90242400	-1.22123700	-1.03105300
C	1.63014300	-1.13162500	1.46292100
C	2.30457900	-1.89565100	0.30220100
H	1.92922900	-1.92474200	-1.86988300
H	2.61688300	-0.41343200	-1.26244100
H	2.03600500	-0.11424000	1.50583200
H	0.52806000	-1.03819600	1.29287100
H	1.89611400	-2.91697900	0.30623500
H	-2.01489800	4.34711000	0.59996700
C	3.82753500	-1.98073300	0.52042100
H	4.22052100	-0.95569100	0.59156200
H	4.05052400	-2.46687200	1.48030900
C	4.57163900	-2.72692700	-0.58805500
H	4.36189400	-2.24813800	-1.55185800
H	4.18016000	-3.75163600	-0.66452100
C	6.08632900	-2.77620400	-0.36762600
H	6.46970200	-1.74934900	-0.28858600
H	6.30022400	-3.25409100	0.59817100
C	6.83012300	-3.51643800	-1.48182000
H	6.65843500	-3.03897300	-2.45331500
H	7.91062500	-3.53534100	-1.30489800
H	6.48676000	-4.55404700	-1.56142900
C	1.72163300	-1.77139300	2.85345500
H	1.35037900	-2.80230600	2.79351900
H	2.77466100	-1.83739400	3.14816700
C	0.93935500	-1.00071800	3.91904600
H	1.29249900	0.03381100	3.99546000
H	-0.12787800	-0.96984100	3.67832500
H	1.04552200	-1.46286300	4.90557200
V	0.08627300	-0.32166000	-0.53909000
N	-1.39105100	-1.10651200	-0.52755700
C	-2.54340700	-1.84554700	-0.55418200
C	-3.27555400	-1.96748700	-1.76247200
C	-3.01819400	-2.47181300	0.62604700
C	-4.49155800	-2.64994000	-1.75244300
C	-4.23812900	-3.14676700	0.59103500
C	-4.98718100	-3.22818400	-0.58325700
H	-5.05564500	-2.73137400	-2.67875400
H	-4.60417600	-3.61519900	1.50168800
H	-5.93741700	-3.75375300	-0.59227700
C	-2.19720200	-2.40269600	1.88280100

H	-1.21960100	-2.88103400	1.74343400
H	-1.99849300	-1.36472700	2.16244100
H	-2.70445700	-2.89757700	2.71594900
C	-2.71844000	-1.37044000	-3.02419100
H	-2.54539200	-0.29670200	-2.91204300
H	-1.74697300	-1.81735900	-3.27061800
H	-3.39439600	-1.52756700	-3.86979000

$^{V}IM_{B21}$

F	4.88273900	0.08866600	1.20577700
F	4.92707300	0.09410800	-1.93316700
F	3.51839600	-1.71727100	5.32674300
F	2.43550300	0.72669400	-2.88193800
F	3.12113800	2.21770200	1.58458100
F	2.01812500	-3.12147900	0.06546300
F	5.45591100	-0.63144200	3.72959900
F	0.39281300	-1.36025000	1.84993800
F	3.49484800	-5.06291700	-0.96995600
F	6.37156400	-1.87087600	-2.93694300
F	3.39126500	4.68725500	0.70540500
F	5.70393700	-4.48437300	-2.49242900
F	2.74866500	3.23840400	-3.74634500
F	0.98363100	-2.05854700	4.32880000
F	3.22973000	5.26158100	-1.96712600
N	-0.19060200	0.52558200	-0.39809400
N	-1.16956100	-1.26766300	-1.11566600
C	-1.42405400	-0.00850200	-0.66048200
C	0.84493100	-0.38652200	-0.61456400
C	0.18363300	-1.49131200	-1.12298700
H	0.58057700	-2.43194800	-1.45610200
C	-0.17015100	1.95913500	-0.16683300
C	-0.07963100	2.48545900	1.14000000
C	-0.14590300	3.87642600	1.28541900
H	-0.05480100	4.30125200	2.27988700
C	-0.28526900	4.71473700	0.18074900
H	-0.31408100	5.79085900	0.31743200
C	-0.34777200	4.17479000	-1.10132400
H	-0.41134300	4.82962000	-1.96496000
C	-0.27879200	2.79067100	-1.30813200
C	0.15985900	1.62577600	2.34672300
H	-0.13673700	2.14784100	3.25653300
H	1.22627300	1.41180500	2.42682700
H	-0.35313600	0.66660500	2.29385600
C	-0.26249800	2.26205200	-2.72228100

H	0.04967600	1.22070800	-2.77949700
H	-1.24191800	2.36339900	-3.19848800
H	0.43993500	2.83951300	-3.32554700
C	-2.16120600	-2.23489900	-1.51431800
C	-2.33727500	-3.37739300	-0.71148400
C	-3.37513100	-4.24823600	-1.05483000
H	-3.55148000	-5.12080800	-0.43451700
C	-4.17568100	-4.01267100	-2.17035400
H	-4.98037700	-4.69878500	-2.41353000
C	-3.91623500	-2.92449600	-2.99698800
H	-4.49480400	-2.77968100	-3.90474200
C	-2.89176100	-2.01977600	-2.69456400
C	-1.42792800	-3.71388900	0.44318800
H	-0.54089800	-4.25100700	0.08831800
H	-1.06069000	-2.84083000	0.97795300
H	-1.94165100	-4.36863800	1.15043100
C	-2.55768000	-0.93455300	-3.68828800
H	-3.41621000	-0.28212800	-3.87755300
H	-1.71277000	-0.32121900	-3.37612700
H	-2.29147200	-1.38667900	-4.64893700
C	2.61976300	-0.57531200	1.39855400
C	1.68806500	-1.15929700	2.24916100
C	1.95491100	-1.53634000	3.56177600
C	3.23592500	-1.36252500	4.07116900
C	4.21904400	-0.80455300	3.25512800
C	3.89585700	-0.43707100	1.95337300
C	2.81178000	1.31571300	-0.61409600
C	3.06900100	2.38318100	0.24285600
C	3.21786300	3.70248900	-0.18557800
C	3.13451400	3.99932700	-1.53901900
C	2.90625000	2.96372000	-2.43997800
C	2.74165000	1.67201100	-1.96264700
C	3.33159400	-1.38663600	-0.93918500
C	4.48996900	-1.15422900	-1.68813400
C	5.28547100	-2.17050400	-2.21756800
C	4.95533400	-3.50129000	-1.98901000
C	3.83283000	-3.79086300	-1.22124600
C	3.07467700	-2.74312700	-0.71131000
B	2.42093100	-0.22306800	-0.19903300
C	-3.64019200	2.84737500	1.16397400
C	-3.58859000	1.96775600	-1.97631400
C	-4.84343200	2.90780900	0.54852700
H	-2.87622400	3.59325300	0.93403500
H	-3.06004700	2.92484100	-1.82837700

H	-2.89307300	1.28531700	-2.49603100
H	-5.65011400	2.22918100	0.81096500
C	-4.87518800	2.12527700	-2.76047300
H	-5.29653800	1.13946100	-2.96360600
H	-5.62212700	2.66500100	-2.16754400
C	-4.64296900	2.86725500	-4.08638900
H	-3.92821900	2.32990700	-4.71831300
H	-5.57858400	2.96357800	-4.64441000
H	-4.24794100	3.87366400	-3.91462000
H	-5.05638200	3.66237600	-0.20026000
C	-3.33227100	1.87780700	2.27226500
H	-4.19552800	1.23338800	2.45594800
H	-2.48514200	1.21133400	2.00248500
C	-2.92620500	2.59748500	3.57617000
H	-3.74716000	3.26562400	3.86147300
H	-2.05856900	3.23504300	3.38103300
C	-2.62754900	1.61809300	4.71487800
H	-3.53115200	1.03401700	4.93297500
H	-1.86466800	0.90008700	4.39017100
C	-2.15479600	2.33202800	5.98291700
H	-1.96125300	1.61637100	6.78608000
H	-1.22815000	2.88698100	5.80074900
H	-2.90550700	3.04371200	6.34148200
V	-3.06204900	1.10959000	-0.28098500
N	-4.10223400	-0.11729200	-0.02155700
C	-4.81776200	-1.20760800	0.40008000
C	-5.98763700	-1.58666700	-0.31590400
C	-4.36724500	-1.95052200	1.53160900
C	-6.68602400	-2.71165000	0.11165100
C	-5.10627200	-3.06946300	1.91031000
C	-6.25248900	-3.44952900	1.21343300
H	-7.57802600	-3.01454800	-0.42702700
H	-4.77594100	-3.65032200	2.76580500
H	-6.80991600	-4.32538800	1.52960000
C	-3.15606700	-1.55461900	2.32991800
H	-2.37627400	-1.11204100	1.70966400
H	-3.41928900	-0.82913500	3.10647600
H	-2.72961900	-2.42140000	2.83644000
C	-6.44133500	-0.80672300	-1.51332800
H	-5.70432300	-0.88998200	-2.31780400
H	-7.39544600	-1.18423400	-1.88552700
H	-6.55726300	0.25558100	-1.28100100

$^{V}TS_{B21}$

F	4.71994700	0.92266100	1.47014300
F	5.12121500	0.18386500	-1.59225500
F	3.07561000	0.00321300	5.77858800
F	2.64976100	0.15742900	-2.85573100
F	2.71089500	2.77139300	1.11979600
F	2.43380100	-2.90110500	0.86412700
F	5.04164600	0.96180500	4.13723200
F	0.43800200	-1.07628500	2.06246400
F	4.25127200	-4.78846900	0.43483200
F	6.90395800	-1.72158700	-1.99217500
F	2.69695300	4.96501800	-0.33905900
F	6.52393500	-4.24054700	-1.00096200
F	2.68310600	2.40225100	-4.30766900
F	0.76679800	-1.00556200	4.67029400
F	2.70249200	4.84461500	-3.07297300
N	-0.11973300	0.24613900	-0.59008400
N	-0.85113100	-1.74814700	-0.97511200
C	-1.25128400	-0.46369800	-0.84782000
C	1.01514100	-0.57545500	-0.50705000
C	0.51364500	-1.82986400	-0.79544300
H	1.01688000	-2.77887800	-0.83038300
C	-0.28286700	1.68600400	-0.68381900
C	-0.36782900	2.49917800	0.46668400
C	-0.57777200	3.87126800	0.27755500
H	-0.62300600	4.51413800	1.15069700
C	-0.70869500	4.41761200	-0.99626100
H	-0.85117500	5.48649000	-1.11635300
C	-0.63672900	3.59451000	-2.11991600
H	-0.70955900	4.02470600	-3.11399900
C	-0.41479500	2.21851900	-1.99267000
C	-0.20045200	1.97684800	1.86382900
H	-0.88524100	2.48614900	2.54746700
H	0.81802900	2.17705900	2.20178300
H	-0.36085600	0.90644900	1.92725600
C	-0.26718900	1.37619400	-3.23983500
H	0.23162700	0.42804700	-3.04868900
H	-1.23619500	1.16457300	-3.70713900
H	0.32816100	1.91197500	-3.98131100
C	-1.75366500	-2.83875800	-1.23749900
C	-2.14996800	-3.65080400	-0.16581200
C	-3.08638300	-4.65626400	-0.43716200
H	-3.42471000	-5.29008800	0.37600300
C	-3.57595100	-4.85180500	-1.72585800
H	-4.29734500	-5.64007300	-1.91607500

C	-3.12365300	-4.05725200	-2.78051400
H	-3.47256600	-4.24456700	-3.79146300
C	-2.19398100	-3.03602700	-2.55762600
C	-1.56072300	-3.48882200	1.20999200
H	-0.57992500	-3.97432600	1.26807200
H	-1.40362000	-2.44419800	1.47352600
H	-2.20805900	-3.94390600	1.96110200
C	-1.63832600	-2.22602700	-3.70330400
H	-1.99768500	-2.61569100	-4.65740800
H	-1.91678300	-1.16971300	-3.64778000
H	-0.54437800	-2.25881300	-3.71387400
C	2.56342100	-0.04062600	1.61943200
C	1.62319200	-0.55577300	2.50496500
C	1.75727200	-0.53653900	3.88944100
C	2.91900000	-0.02436900	4.45299000
C	3.91603300	0.46888400	3.61181400
C	3.72363300	0.43984600	2.23444400
C	2.74182900	1.32702200	-0.79087400
C	2.76773700	2.59683300	-0.21982000
C	2.76494500	3.77956900	-0.95985500
C	2.76222300	3.72397800	-2.34629300
C	2.76434100	2.47894000	-2.96787200
C	2.74314800	1.33173700	-2.18846400
C	3.63154200	-1.25858600	-0.39724900
C	4.82433000	-1.02950700	-1.09060900
C	5.79289400	-2.00967700	-1.30623200
C	5.60809500	-3.29083800	-0.79950100
C	4.45302700	-3.56625900	-0.07592600
C	3.51860300	-2.55459700	0.11533100
B	2.50817800	-0.10971300	-0.02547400
C	-3.69518300	2.46577500	-0.29305800
C	-4.04943200	0.44783600	-2.50581300
C	-4.64766400	2.19105500	-1.28024300
H	-2.84266400	3.06642100	-0.62018500
H	-3.25816300	0.90540200	-3.12108200
H	-3.67569000	-0.57595000	-2.13768200
H	-5.62882700	1.81701300	-1.00293800
C	-5.31823600	0.19658100	-3.28747900
H	-6.11756800	-0.09629400	-2.60217600
H	-5.62549100	1.13142300	-3.76745400
C	-5.11724000	-0.89417000	-4.35276200
H	-4.82970300	-1.84407600	-3.89202400
H	-6.04633000	-1.05137100	-4.90587500
H	-4.33998700	-0.61187900	-5.06884400

H	-4.58463700	2.69076200	-2.24084700
C	-3.99693400	2.52826900	1.18920700
H	-4.86186500	1.89947600	1.42455400
H	-3.15068900	2.13502000	1.75965400
C	-4.24630800	3.98036400	1.63857000
H	-5.12162000	4.37879600	1.10983000
H	-3.39201000	4.60128200	1.34011000
C	-4.45294400	4.08461400	3.15304700
H	-5.30092700	3.45232900	3.44674800
H	-3.57122000	3.67415000	3.66386100
C	-4.69372300	5.52282700	3.61632800
H	-4.83619700	5.56927400	4.69939800
H	-3.84476100	6.16690500	3.36334300
H	-5.58617800	5.94610100	3.14359400
V	-3.03079800	0.48844400	-0.81009300
N	-3.80350500	-0.42884800	0.28172800
C	-4.31468700	-1.31245900	1.20622800
C	-5.33078300	-2.21723900	0.79938000
C	-3.78089000	-1.32990300	2.52137800
C	-5.80216000	-3.13327300	1.73422400
C	-4.29542300	-2.26710500	3.41841600
C	-5.29387600	-3.15946900	3.03523600
H	-6.57428600	-3.83684400	1.43949100
H	-3.89446400	-2.30036600	4.42678400
H	-5.67582800	-3.88195600	3.74913700
C	-2.68711200	-0.39230400	2.94809400
H	-2.18466200	0.04456100	2.08760100
H	-3.08805400	0.42733700	3.55537500
H	-1.92777500	-0.90377900	3.54538500
C	-5.86000600	-2.19705300	-0.60550000
H	-5.06071300	-2.37819800	-1.33107100
H	-6.61711000	-2.97041200	-0.74619700
H	-6.31978800	-1.23082300	-0.83669000

$^{V}PR_{B21}$

F	4.71301900	1.02248700	1.50978400
F	5.12017300	0.60438700	-1.58306600
F	3.22432900	-0.43591600	5.72502900
F	2.65120000	0.56407500	-2.83542400
F	2.54995900	2.75172600	1.38674200
F	2.62804000	-2.86592500	0.54251500
F	5.09022200	0.81101100	4.16227500
F	0.57870900	-1.29915300	1.95516900
F	4.54521100	-4.59214600	-0.08336400

F	7.00441600	-1.14384000	-2.18051000
F	2.39647900	5.08275700	0.16003400
F	6.77453500	-3.76995100	-1.45633500
F	2.53166100	2.94654800	-4.05007600
F	0.96248200	-1.47416200	4.55503700
F	2.39836700	5.24265100	-2.57005400
N	-0.10319600	0.27086200	-0.53056200
N	-0.74528500	-1.72238700	-1.06914400
C	-1.20962900	-0.47232100	-0.82019800
C	1.06583200	-0.48976600	-0.54523900
C	0.61987900	-1.74388900	-0.93940800
H	1.17496100	-2.65158600	-1.09174600
C	-0.32838500	1.70388500	-0.45936300
C	-0.46901800	2.36086800	0.77904000
C	-0.75160600	3.73201500	0.75495900
H	-0.84607600	4.25989600	1.69833300
C	-0.90227200	4.42009300	-0.44615600
H	-1.10104800	5.48665800	-0.43894000
C	-0.78316100	3.74277700	-1.65963900
H	-0.88120500	4.28299200	-2.59604900
C	-0.49454500	2.37401900	-1.69449200
C	-0.31392300	1.66255100	2.09840300
H	-0.94377600	2.13474900	2.85640300
H	0.72271400	1.73751200	2.43206400
H	-0.56385200	0.60713000	2.03535900
C	-0.35429000	1.67449600	-3.02721400
H	0.29128100	0.79955200	-2.97279500
H	-1.32506500	1.34800800	-3.42064500
H	0.07954800	2.35336000	-3.76351800
C	-1.57814600	-2.83654000	-1.44425200
C	-1.85603800	-3.81529400	-0.48029000
C	-2.67749900	-4.88042700	-0.86868600
H	-2.92138400	-5.64607300	-0.13954200
C	-3.17226000	-4.96830400	-2.16720200
H	-3.79803400	-5.80832300	-2.45123800
C	-2.85694100	-3.98957000	-3.11035700
H	-3.22247700	-4.07784400	-4.12880300
C	-2.04981000	-2.90007400	-2.76713900
C	-1.27541800	-3.74649200	0.90695000
H	-0.23954000	-4.10340300	0.91770600
H	-1.25663600	-2.72716700	1.29028200
H	-1.85402300	-4.36501300	1.59514200
C	-1.67141500	-1.86276100	-3.79593700
H	-1.91881300	-2.21301600	-4.79978700

H	-2.19615500	-0.91352800	-3.64201400
H	-0.60120300	-1.63953500	-3.76756000
C	2.62496800	-0.08747400	1.59381200
C	1.73539400	-0.74655800	2.43521500
C	1.90061200	-0.86303100	3.81177400
C	3.04039000	-0.33658900	4.40654400
C	3.98648400	0.30304400	3.60626600
C	3.76608800	0.40238300	2.23667900
C	2.68292100	1.52242000	-0.66346600
C	2.62424400	2.72435800	0.03713300
C	2.54215500	3.97376900	-0.57752100
C	2.53747800	4.05985000	-1.96251700
C	2.61597200	2.88915400	-2.71054200
C	2.67746000	1.66968600	-2.05238500
C	3.72122600	-1.03646900	-0.54571400
C	4.89502500	-0.66915900	-1.21167400
C	5.91606400	-1.56501900	-1.52832100
C	5.80739300	-2.90037100	-1.15752400
C	4.67430200	-3.31463100	-0.46586400
C	3.68568300	-2.38283400	-0.17032300
B	2.54427900	0.00547100	-0.04785700
C	-3.67244300	2.04349200	0.06212400
C	-4.40141100	0.86295400	-2.24573900
C	-4.76677200	1.77769800	-1.01454500
H	-2.92912000	2.76074100	-0.31267500
H	-3.55077900	1.28708000	-2.80364000
H	-4.15221900	-0.21916400	-1.98881900
H	-5.68265500	1.40717600	-0.55171800
C	-5.58418700	0.64467500	-3.20410700
H	-6.43263700	0.26662300	-2.62366500
H	-5.87746800	1.62145200	-3.60224300
C	-5.23692600	-0.32150300	-4.33750900
H	-4.96993600	-1.31025700	-3.94883300
H	-6.09187800	-0.44603700	-5.00581300
H	-4.39750200	0.04809400	-4.93495400
H	-4.96124300	2.74638900	-1.48491200
C	-4.15234600	2.38262900	1.45074800
H	-4.87493200	1.62954000	1.78643400
H	-3.29160300	2.32641600	2.12656800
C	-4.76329800	3.79179500	1.55041000
H	-5.64091400	3.86070100	0.89318700
H	-4.03493700	4.52320800	1.17589400
C	-5.16890800	4.15549000	2.98213200
H	-5.88490200	3.41108500	3.35406800

H	-4.28724100	4.08542100	3.63267400
C	-5.77710200	5.55567700	3.08494500
H	-6.05599200	5.79006800	4.11585300
H	-5.06832200	6.32019100	2.74903900
H	-6.67803800	5.64191600	2.46800200
V	-3.02789900	0.32857700	-0.61516400
N	-3.69151600	-0.75527000	0.38684800
C	-4.15208800	-1.77804700	1.17990600
C	-5.03331200	-2.72722200	0.59792000
C	-3.72044600	-1.87237500	2.52727700
C	-5.46495100	-3.78398000	1.39294500
C	-4.18673200	-2.95350000	3.27678500
C	-5.04349600	-3.90157200	2.71999400
H	-6.13968300	-4.52178600	0.97039800
H	-3.86777400	-3.05106000	4.30976700
H	-5.39208400	-4.73318300	3.32369600
C	-2.75637900	-0.88370100	3.12089300
H	-2.87726900	0.10978500	2.68596700
H	-2.90091500	-0.80694300	4.20103700
H	-1.71733300	-1.19282900	2.95731200
C	-5.47530000	-2.58476000	-0.83156000
H	-4.61869000	-2.61269800	-1.51198900
H	-6.14731700	-3.39737900	-1.11261100
H	-6.00949600	-1.64019800	-0.99022400

$^{V}IM_{B12}$

F	4.72379900	0.92932900	1.49600000
F	4.74907600	2.13395000	-1.39734600
F	4.06917900	-2.64302400	4.48432900
F	2.15947100	2.24861700	-2.37825900
F	2.33940000	2.03632300	2.37102400
F	3.07478200	-2.33045300	-1.20216100
F	5.49295100	-0.45628400	3.66583500
F	1.08671500	-2.08457400	0.91786600
F	5.09275100	-3.12842600	-2.72905800
F	6.74894800	1.29470400	-2.90460400
F	1.70742000	4.59331200	2.49472500
F	6.97360200	-1.33668600	-3.61244800
F	1.54605300	4.84608100	-2.21945900
F	1.84530100	-3.41638800	3.05579900
F	1.31368900	6.06269000	0.22309700
N	-0.18612900	0.24639000	-0.32751600
N	-0.59255300	-1.24999000	-1.83355400
C	-1.19430600	-0.47211200	-0.89586200

C	1.06905200	-0.08902500	-0.84968700
C	0.76409100	-1.00777500	-1.83405900
H	1.41518100	-1.53513300	-2.50648200
C	-0.60554600	1.39310800	0.45925900
C	-0.66072000	1.33945500	1.86852300
C	-1.13343200	2.47714700	2.53755300
H	-1.17399800	2.46053900	3.62196100
C	-1.49220600	3.62961300	1.84443700
H	-1.81580800	4.51052700	2.38915900
C	-1.40947300	3.66477600	0.45167500
H	-1.66516200	4.57273700	-0.08514200
C	-0.98353000	2.54778200	-0.26914300
C	-0.17205300	0.16949000	2.66959300
H	-0.75816700	0.05868200	3.58360700
H	0.86657300	0.34906600	2.95450500
H	-0.20916100	-0.76000000	2.11110600
C	-0.95425900	2.59725200	-1.77729400
H	-0.15659200	1.98895000	-2.20214400
H	-1.90112100	2.24553700	-2.20596000
H	-0.80069500	3.62222400	-2.11929900
C	-1.27562600	-2.20737900	-2.66229700
C	-1.00423300	-3.57491200	-2.48364500
C	-1.75369900	-4.47878500	-3.24869900
H	-1.57235400	-5.54202200	-3.12657700
C	-2.70425600	-4.03630500	-4.16459500
H	-3.26946200	-4.75609700	-4.74772700
C	-2.91111800	-2.67080200	-4.35665100
H	-3.62075000	-2.32792800	-5.10334500
C	-2.19332500	-1.72860900	-3.61550700
C	0.07459100	-4.08039800	-1.55891600
H	-0.14535700	-5.10205300	-1.24134700
H	1.04328700	-4.09979800	-2.07116000
H	0.20936800	-3.46081400	-0.67465300
C	-2.38500800	-0.25271300	-3.86555300
H	-2.85370200	-0.09118900	-4.83842700
H	-3.03128200	0.22238600	-3.11529600
H	-1.43456900	0.28576900	-3.84823800
C	2.85399500	-0.46941800	1.11268400
C	2.20420100	-1.61704100	1.55103600
C	2.57248200	-2.34833500	2.67538800
C	3.69087600	-1.95972400	3.40124700
C	4.40975900	-0.83962800	2.98348700
C	3.98877400	-0.13692300	1.85901500
C	2.34896700	2.00799200	-0.02422400

C	2.22430900	2.68183000	1.18844200
C	1.89432700	4.03289700	1.29199600
C	1.69027900	4.78221800	0.14187200
C	1.81870400	4.16156400	-1.09691300
C	2.13475300	2.81172500	-1.14668700
C	3.74319700	-0.03227600	-1.27042400
C	4.75189400	0.82711800	-1.71743900
C	5.82861800	0.41275300	-2.50143200
C	5.95188400	-0.92500000	-2.85897100
C	4.99416700	-1.82955100	-2.41376200
C	3.94222000	-1.36891000	-1.63017300
B	2.51673000	0.38939200	-0.25297800
C	-4.10001200	1.72589200	0.08966300
C	-4.32979400	-1.39769000	-0.93847800
C	-5.33739500	1.14884900	0.06236100
H	-3.57844600	1.89891500	1.03336400
H	-3.74443900	2.28297600	-0.77581900
H	-4.62443900	-0.98138700	-1.91186900
H	-3.29753000	-1.86577600	-1.08738000
H	-5.71424700	0.68960600	0.97506800
C	-5.29246800	-2.43096500	-0.40378500
H	-4.95356000	-2.76456400	0.58169200
H	-6.27112100	-1.95517500	-0.26216600
C	-5.43718900	-3.63105100	-1.35484700
H	-4.47300400	-4.12539100	-1.50955400
H	-6.13648100	-4.36386400	-0.94337200
H	-5.80926500	-3.31655400	-2.33472100
C	-6.27621800	1.20560300	-1.09134700
H	-6.77232100	0.23825900	-1.22664200
H	-5.74077300	1.45037200	-2.01552900
C	-7.36034100	2.28128400	-0.82163400
H	-6.87243600	3.25322000	-0.68210900
H	-7.87913500	2.05061300	0.11722900
C	-8.37716400	2.36660700	-1.96639400
H	-7.84695900	2.57944700	-2.90350200
H	-8.85722000	1.38819800	-2.09573700
C	-9.43946800	3.43886500	-1.71649900
H	-10.15136500	3.48301000	-2.54506600
H	-10.00414400	3.23185100	-0.80148000
H	-8.98502600	4.42970600	-1.61333000
V	-3.03880200	-0.25272300	-0.07293300
N	-3.01458700	-0.93918400	1.39758000
C	-2.86983100	-1.53708300	2.62405500
C	-2.10337000	-2.72691200	2.73603900

C	-3.43699100	-0.90102600	3.76238900
C	-1.89063500	-3.24395700	4.01387600
C	-3.19788300	-1.46547600	5.01113000
C	-2.42567900	-2.62323600	5.14050100
H	-1.28659800	-4.13917600	4.12204600
H	-3.61180700	-0.98918400	5.89429000
H	-2.24091600	-3.03984000	6.12518600
C	-4.23394100	0.36663800	3.62218800
H	-3.62797700	1.17067200	3.19110800
H	-5.10547700	0.21705700	2.97591500
H	-4.59685000	0.70588700	4.59404500
C	-1.54277800	-3.42405700	1.53036500
H	-0.56180900	-3.84512300	1.75560100
H	-2.20247000	-4.24151300	1.21407800
H	-1.43011300	-2.74128500	0.68972200

$^{V}TS_{B12}$

F	-4.72795200	0.58967000	-1.61553800
F	-4.69166200	2.41104200	0.94020500
F	-4.16359600	-3.55630200	-3.76318800
F	-2.09047900	2.68650600	1.83468300
F	-2.33145200	1.42078100	-2.74497500
F	-3.10438000	-2.02375900	1.70989400
F	-5.55013000	-1.22361000	-3.42049300
F	-1.11524600	-2.27844900	-0.44516000
F	-5.11807100	-2.42649700	3.39040200
F	-6.68487500	1.96428600	2.61271200
F	-1.67798300	3.87936700	-3.44399300
F	-6.95234200	-0.44400800	3.87790100
F	-1.45583700	5.17763400	1.09203500
F	-1.92449200	-4.03122700	-2.23419300
F	-1.24395100	5.81471600	-1.56034000
N	0.19029200	0.28250700	0.27317200
N	0.61575500	-0.90111300	2.02676100
C	1.20079400	-0.31288500	0.95703300
C	-1.06127700	0.05238900	0.85854700
C	-0.74376800	-0.66443000	1.99632700
H	-1.38905900	-1.06217000	2.75771700
C	0.63870500	1.21103600	-0.75175300
C	0.68844900	0.83533100	-2.11512900
C	1.16561100	1.78606900	-3.02802600
H	1.19895500	1.52179400	-4.08002500
C	1.53017600	3.06433400	-2.61829400
H	1.85205000	3.79631500	-3.35190500

C	1.45993900	3.41809200	-1.27004700
H	1.72586500	4.42296200	-0.95764500
C	1.03238100	2.50123000	-0.30924300
C	0.17364500	-0.47683500	-2.62671800
H	0.72491900	-0.78314200	-3.51755900
H	-0.87588600	-0.35405300	-2.90189100
H	0.23054000	-1.26674200	-1.88466200
C	1.01915800	2.89459700	1.14716400
H	0.25765500	2.36645000	1.71922000
H	1.98992800	2.69347500	1.61816700
H	0.82310900	3.96339200	1.24854100
C	1.34240600	-1.65770500	3.01123300
C	1.12264200	-3.04221000	3.10309200
C	1.91908900	-3.75613100	4.00869000
H	1.77781300	-4.82878600	4.09660600
C	2.86693700	-3.11243700	4.79949700
H	3.46779400	-3.68608700	5.49783000
C	3.02495100	-1.72894800	4.72056400
H	3.73079100	-1.22407000	5.37319400
C	2.25831200	-0.97201300	3.82961900
C	0.04903000	-3.74619800	2.31228800
H	0.28711100	-4.80663500	2.20469100
H	-0.91563600	-3.67944100	2.82848400
H	-0.10371800	-3.31882700	1.32281500
C	2.38845100	0.53154700	3.79394600
H	2.89310300	0.89028500	4.69336600
H	2.96793900	0.88638400	2.93220800
H	1.41158500	1.01771200	3.73188300
C	-2.87273900	-0.72330500	-0.95759000
C	-2.24003000	-1.94571300	-1.14752400
C	-2.63485800	-2.89719400	-2.08218100
C	-3.76145200	-2.65993900	-2.85896600
C	-4.46158600	-1.46621100	-2.68434300
C	-4.01444800	-0.54311400	-1.74471000
C	-2.31731800	1.93157400	-0.40387200
C	-2.19728100	2.31429200	-1.73802100
C	-1.85617100	3.60427400	-2.14469500
C	-1.63174500	4.58891700	-1.19276600
C	-1.75126300	4.26244700	0.15463900
C	-2.07784500	2.96170700	0.50888500
C	-3.72980200	0.24663100	1.27758000
C	-4.71503600	1.20482200	1.53636500
C	-5.78818600	0.99511100	2.40220100
C	-5.93278800	-0.22959900	3.04377900

C	-4.99892300	-1.23022100	2.79812900
C	-3.94892800	-0.97422000	1.92349200
B	-2.50779100	0.40780500	0.18311600
C	4.11321500	1.45093500	-0.54275300
C	4.51579100	-0.92425800	1.14601500
C	5.19373400	0.74962500	0.07022900
H	4.03354900	1.49231800	-1.62395400
H	3.72114300	2.31760000	-0.01155000
H	4.60691200	-0.47790300	2.13806800
H	3.50229600	-1.46781200	1.16353600
H	5.80505200	0.13786800	-0.58877000
C	5.54599500	-2.00206100	0.87960300
H	5.45362700	-2.33780400	-0.15816500
H	6.54850100	-1.57884600	1.00547600
C	5.36191500	-3.18769800	1.84059800
H	4.37190500	-3.64088900	1.72427400
H	6.11253600	-3.95625000	1.64025600
H	5.46130800	-2.87347200	2.88325100
C	5.93290400	1.40101000	1.22085800
H	6.52923900	0.66869300	1.77326200
H	5.21449000	1.84205300	1.92265400
C	6.86366200	2.50264000	0.67973600
H	6.26433700	3.25458200	0.15189500
H	7.54751500	2.07121500	-0.06306500
C	7.67407100	3.17291600	1.79505300
H	6.98458000	3.58569300	2.54313900
H	8.27386300	2.41343800	2.31280300
C	8.58662800	4.28137100	1.26610100
H	9.15245400	4.74430100	2.07897800
H	9.30612500	3.88916100	0.53965800
H	8.00737700	5.06849400	0.77205500
V	2.99358500	-0.18925300	0.00593900
N	3.07727100	-1.19827400	-1.27073300
C	2.97392400	-2.04912100	-2.33789400
C	2.22447000	-3.25172900	-2.21860600
C	3.58818700	-1.66880900	-3.56531400
C	2.09047300	-4.04765100	-3.35644800
C	3.42161300	-2.50135400	-4.66560500
C	2.67633300	-3.68053000	-4.56587400
H	1.50958000	-4.96231500	-3.29100900
H	3.87378700	-2.22516600	-5.61289500
H	2.55257600	-4.31359900	-5.43850100
C	4.35670200	-0.38034800	-3.67256300
H	3.68939900	0.48178500	-3.56390900

H	5.12425800	-0.30593000	-2.89530500
H	4.85045900	-0.30238000	-4.64286800
C	1.59430900	-3.67854800	-0.92363600
H	0.57141200	-4.02210400	-1.09223900
H	2.15829900	-4.50518500	-0.47509700
H	1.55891800	-2.86461300	-0.20129100

$^{V}PR_{B12}$

F	-4.67046200	1.00884400	-1.55771700
F	-4.72454500	2.13732300	1.34769600
F	-4.04809700	-2.46831500	-4.66274200
F	-2.15517000	2.13260200	2.39979000
F	-2.21445400	2.05419400	-2.35731400
F	-3.19955700	-2.37551500	1.07959400
F	-5.42987700	-0.27316400	-3.79382100
F	-1.13950200	-2.13527400	-1.00589900
F	-5.28261600	-3.14057600	2.53855200
F	-6.78718800	1.33281200	2.78839500
F	-1.51451500	4.59464000	-2.39180000
F	-7.11970300	-1.30587600	3.42400900
F	-1.48714900	4.71991800	2.32924000
F	-1.88008200	-3.35538000	-3.21638300
F	-1.15926700	5.99580700	-0.07104000
N	0.16680500	0.16809900	0.39348000
N	0.52539100	-1.41300100	1.81127200
C	1.15341400	-0.57434200	0.95848800
C	-1.10999900	-0.19561500	0.83836700
C	-0.83528800	-1.17478800	1.77327600
H	-1.50960200	-1.74763500	2.38305000
C	0.68220600	1.29541800	-0.36489100
C	0.75134400	1.26763000	-1.78212000
C	1.24269500	2.41252000	-2.42486300
H	1.29428700	2.41367100	-3.50866200
C	1.59634100	3.54903500	-1.70841000
H	1.92858200	4.43759400	-2.23534000
C	1.50500300	3.56457100	-0.31497100
H	1.76368400	4.46313400	0.23571800
C	1.05646400	2.44780100	0.38678400
C	0.24271300	0.12665800	-2.60820000
H	0.82386600	0.03241000	-3.52702600
H	-0.79408400	0.33164300	-2.88204500
H	0.26382100	-0.81597500	-2.07074300
C	0.98716900	2.47723200	1.89216100
H	0.16546300	1.87987900	2.28519500

H	1.91464900	2.09780300	2.33808600
H	0.84881700	3.50113000	2.24315100
C	1.19375900	-2.39407100	2.62484900
C	1.00410800	-3.75332500	2.32783000
C	1.69489500	-4.68224300	3.11605600
H	1.57125900	-5.74056100	2.90909300
C	2.51814500	-4.26843700	4.16036700
H	3.03748400	-5.00610600	4.76363500
C	2.65960600	-2.91150200	4.44941300
H	3.27309400	-2.59504900	5.28748400
C	1.99215000	-1.94489500	3.69028200
C	0.07105800	-4.21047400	1.23523800
H	0.28623800	-5.24432600	0.95758700
H	-0.97352100	-4.16755800	1.56321000
H	0.13499000	-3.58769700	0.34322100
C	2.09346500	-0.48190200	4.04734600
H	2.51098100	-0.36247200	5.04897500
H	2.73534600	0.07553200	3.35481400
H	1.11422700	0.00417200	4.02523800
C	-2.85629100	-0.46266200	-1.18151600
C	-2.22847800	-1.61200900	-1.64682800
C	-2.59004300	-2.28683400	-2.80823000
C	-3.67918500	-1.83889700	-3.54463500
C	-4.37640100	-0.71525500	-3.10091000
C	-3.96172000	-0.06816100	-1.94143500
C	-2.29236200	1.95979500	0.03638000
C	-2.11778300	2.66351600	-1.15317200
C	-1.75539000	4.00931000	-1.21038900
C	-1.56925000	4.72358000	-0.03537300
C	-1.74427100	4.07229300	1.18179900
C	-2.08836800	2.72821200	1.18578500
C	-3.79143100	-0.05937400	1.19145900
C	-4.78013100	0.82377000	1.63597700
C	-5.88873000	0.42886600	2.38454800
C	-6.06658100	-0.91213000	2.70513900
C	-5.13057000	-1.83899300	2.25922900
C	-4.04449600	-1.39641200	1.51264500
B	-2.52443200	0.34073900	0.21702900
C	4.07710700	1.42111000	0.11541200
C	4.53313700	-1.07469400	1.15402700
C	5.07870000	0.30960400	0.55371700
H	4.39963800	1.98020600	-0.75634100
H	3.77013600	2.06849100	0.94134500
H	4.35588300	-0.95668100	2.22625000

H	3.56829800	-1.55412400	0.78199200
H	5.68211800	0.03888000	-0.31375100
C	5.50663200	-2.22882600	0.87193600
H	5.64820100	-2.29976500	-0.21222800
H	6.47850600	-1.97954800	1.30970100
C	4.99210300	-3.55500400	1.43422800
H	4.03093100	-3.82963100	0.98568800
H	5.70092000	-4.35979500	1.22578300
H	4.84483700	-3.50087200	2.51602200
C	5.98862000	0.89175300	1.67537700
H	6.62848700	0.08737100	2.05454100
H	5.35344100	1.21385100	2.50987200
C	6.86272500	2.05004900	1.19379000
H	6.23008800	2.88035300	0.85694600
H	7.44387700	1.72674900	0.31936600
C	7.81782900	2.54954700	2.28425200
H	7.23351200	2.86003100	3.16035700
H	8.45393000	1.71914600	2.61677100
C	8.69218500	3.71181400	1.80893100
H	9.36005600	4.05270100	2.60449400
H	9.31256800	3.41698900	0.95590200
H	8.08046300	4.56553000	1.49807600
V	2.88319500	-0.09856400	-0.02478500
N	3.12718300	-0.73326100	-1.51388900
C	3.14825000	-1.27098600	-2.76555900
C	2.47258000	-2.49498700	-3.03656500
C	3.83468300	-0.54897400	-3.78777800
C	2.49524800	-2.96916900	-4.34809500
C	3.81437400	-1.06497600	-5.07682800
C	3.14963900	-2.26451300	-5.35661000
H	1.98503400	-3.89861200	-4.58110600
H	4.32274500	-0.52997800	-5.87261600
H	3.14596400	-2.65102900	-6.37074500
C	4.53665100	0.74326700	-3.46946300
H	3.83255400	1.49019800	-3.08944500
H	5.30433300	0.59924100	-2.70155600
H	5.02198100	1.14906400	-4.35892100
C	1.74076400	-3.26245700	-1.97198200
H	0.66226000	-3.25287000	-2.15929200
H	2.06012000	-4.30929200	-1.96733900
H	1.90828200	-2.84681700	-0.97816400
